# Chiral Autoamplification Meets Dynamic Chirality Control to Suggest Nonautocatalytic Chemical Model of Prebiotic Chirality Amplification

**DOI:** 10.34133/2019/4756025

**Published:** 2019-11-04

**Authors:** Evgenii P. Talsi, Anna A. Bryliakova, Roman V. Ottenbacher, Tatyana V. Rybalova, Konstantin P. Bryliakov

**Affiliations:** ^1^Novosibirsk State University, Pirogova 2, Novosibirsk 630090, Russia; ^2^Boreskov Institute of Catalysis, Pr. Lavrentieva 5, Novosibirsk 630090, Russia; ^3^Vorozhtsov Novosibirsk Institute of Organic Chemistry, Pr. Lavrentieva 9, Novosibirsk 630090, Russia

## Abstract

Oxidative kinetic resolution of 1-phenylethanol in the presence of manganese complexes, bearing conformationally nonrigid *achiral bis*-amine*-bis*-pyridine ligands, in the absence of any exogenous chiral additives, is reported. The only driving force for the chiral discrimination is the small initial enantiomeric imbalance of the scalemic (nonracemic) substrate: the latter dynamically controls the chirality of the catalyst, serving itself as the chiral auxiliary. In effect, the *ee* of 1-phenylethanol increases monotonously over the reaction course. This dynamic control of catalyst chirality by the *substrate* has been unprecedented; a consistent kinetic model for this process is presented. The reported catalyzed substrate self-enantioenrichment mechanism is discussed in relation to the problem of prebiotic chirality amplification.

## 1. Introduction

The origin of chirality in living systems has been debated since the time of Pasteur. Today the early assumption of Terent'ev and Klabunovskii that “life cannot and never could exist without molecular dissymmetry” [1] has become widespread, with the further realization that absolute chiral homogeneity is essential for the existence of life [2]. Moreover, it has been hypothesized that the chiral purity observed in the biosphere was achieved at the stage of prebiotic evolution and was a necessary condition for the subsequent development of self-replication characteristic of living matter [3]. These considerations put forward an abiotic and not a biotic mechanism as the primordial origin of chirality and chiral homogeneity—whether on Earth or elsewhere in the universe [2].

The question remains how the initial prebiotic enantiomeric imbalance could come about on Earth [2, 3]; existing hypotheses invoke either stochastic processes (operated by “chance,” without chemical or physical directing forces) or determinate processes (directed by external chiral forces) [4–9], or assume extraterrestrial chirality protosource [10] without considering the problem in essence. However, regardless of the origin of this initial (likely small) enantiomeric imbalance, there should be an efficient mechanism of *ee* amplification, transforming the slightly nonracemic mixture of reactants into an enantiomerically enriched, ideally single-handed state. Crucially, such mechanism should not require assistance of other nonracemic chiral substances (e.g., catalysts), since simultaneous emergence of two different chirality protosources (on Earth), capable of readily reacting with each other, should be a less likely event. Asymmetric autocatalysis has been widely invoked when considering the problem of the origin of biological homochirality from the chemical perspective [11–14]. The autocatalytic Soai et al. reaction [15] provided one model for the scenario described above: the possibility of reproducible amplification of negligibly small enantiomeric imbalance (down to 5∙10^−5^%*ee*) has been established in this reaction [16].

On the other hand, hypothetical chemical mechanisms of chirality amplification should not necessarily be autocatalytic, mediated by the chiral reaction product. The applicability of kinetic resolution to the problem of enantiomeric enrichment was emphasized in a fundamental review [17], yet previously known examples of kinetic resolutions were concluded to require contrived experimental conditions, making them prebiotically unrealistic [18].

At the same time, the possibility of the reaction *substrate* itself being the raw material whose mirror symmetry was initially broken could be considered. Such substrate with small enantiomeric imbalance, which came to be in the prebiotic soup, could direct its own kinetic resolution, catalyzed by third substance(s), assembled from achiral reactants available from the primitive environment. Herewith, experimental model of such scenario based on the self-directed oxidative kinetic resolution of scalemic 1-phenylethanol is presented. It has been demonstrated that for the kinetic resolution to take place, the catalyst chirality should not be compulsorily predetermined by the chiral, enantiomerically pure ligand. The process is efficiently conducted by stereolabile manganese complexes bearing achiral tetradentate ligands; the catalyst chirality is dynamically determined by coordination of the chiral substrate to the Mn active sites.

## 2. Results and Discussion

### 2.1. Kinetic Resolution of Scalemic 1-Phenylethanol in the Presence of Complexes 1 and 2

**Figure 1:**
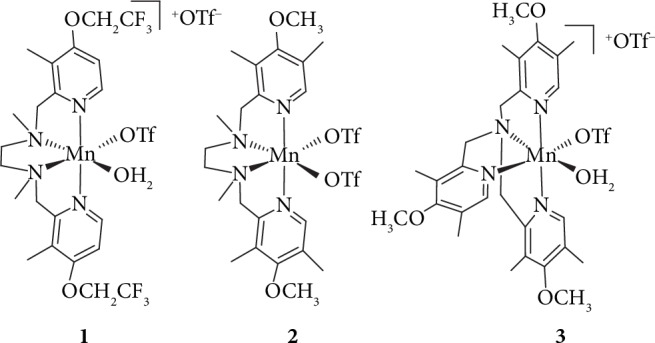
Structures of Mn complexes **1-3**.

**Figure 2:**
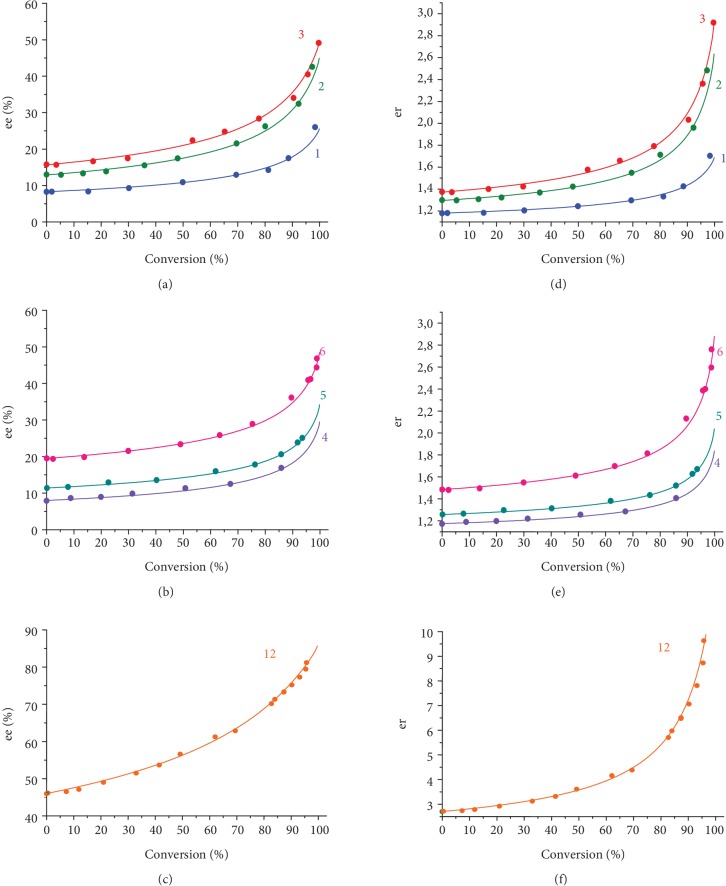
Enantiomeric excess (*ee*) vs. conversion plots for the kinetic resolution of scalemic (*S*)-1-phenylethanol in the presence of catalysts **1** (a, c) and **2** (b). Enantiomeric ratio (*er*) [S]/[R] vs. conversion plots for the kinetic resolution of scalemic 1-phenylethanol in the presence of achiral catalysts **1** (d, f) and **2** (e). Dots represent experimental data; solid lines are theoretical curves with parameters *A* and *B* given in Table 1. Curve numbers correspond to the numbers of entries in Table 1.

**Figure 3:**
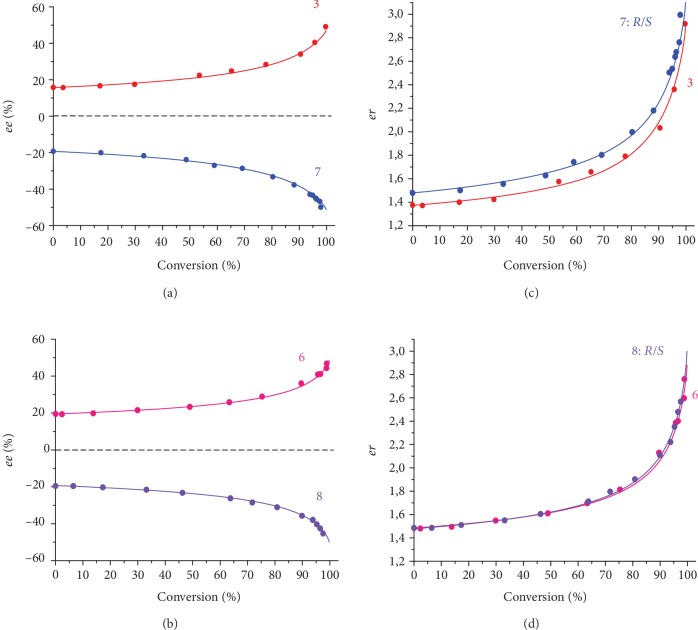
Enantiomeric excess (*ee*) vs. conversion plots for the kinetic resolution of scalemic (*S*)-1-phenylethanol and (*R*)-1-phenylethanol in the presence of catalysts **1** (a) and **2** (b). Enantiomeric ratio (*er*: [S]/[R] for (*S*)-substrate or [R]/[S] for (*R*)-substrate) vs. conversion plots for the kinetic resolution of scalemic (*S*)-1-phenylethanol and (*R*)-1-phenylethanol in the presence of achiral catalysts **1** (c) and **2** (d). Dots represent experimental data; solid lines are theoretical curves with parameters *A* and *B* given in Table 1. Curve numbers correspond to the numbers of entries in Table 1.

In this work, it has been found that Mn complexes **1** and **2** (Figure 1), bearing *achiral* ligands, efficiently catalyze the oxidation of 1-phenylethanol with aqueous H_2_O_2_, using 0.1 mol.% catalyst loading (Table 1). The oxidation of the *sec*-alcohol to the corresponding ketone proceeds with very high selectivity, affording no detectable side products (SI, pages 26-48), so the amount of the residual alcohol (Figures 2 and 3) can be calculated as (100% − %conversion) with high accuracy.

Crucially, the small initial enantiomeric excess of scalemic substrate of ca. 8% *ee* ((*S*)-configuration) has been shown to increase up to 17-26% *ee* (entries 1 and 4 of Table 1). When 1-phenylethanol with higher initial enantiomeric excess was taken to the oxidation, the *ee* growth was steeper, and the final *ee* was higher (cf. entries 1-3 and 4-6 and 12). When racemic 1-phenylethanol was oxidized in the presence of **1**, no enantioenrichment was observed. The *ee* and *er* vs. conversion plots for the experiments 1-6 of Table 1 are presented in Figure 2; one can see that the enantiomeric excess grows up monotonously, this growth becoming steeper with increasing conversion.

Eventual contamination of 1-phenylethanol with any catalytically active chiral species can be ruled out since (1) the oxidation did not occur in the absence of added Mn complex (**1**, **2**, or **3**) within at least 1 day at -10°C and (2) scalemic 1-phenylethanols of different origin—either synthesized ((*S*)-1-phenylethanol) or commercial ((*R*)-1-phenylethanol) showed identical results, with the only exception for the sign of chirality (Figure 3). Moreover, manganese complex **3** with achiral tripodal aminopyridine ligand also efficiently catalyzed the oxidation of scalemic 1-phenylethanol, but was unable to ensure *ee* amplification (Table 1, entry 9, [Supplementary-material supplementary-material-1], SI). When scalemic methyl and butyl mandelates ((*R*)-configuration) were oxidized in the presence of complex **2**, no *ee* enhancement was observed, either (entries 10, 11, [Supplementary-material supplementary-material-1], SI).

### 2.2. The Origin of 1-Phenylethanol Enantioenrichment in the Presence of Mn Complexes Bearing Achiral Ligands

These intriguing results deserve detailed discussion. The fundamental difference between complexes **1**, **2**, and **3** is that, in contrast to the truly achiral **3**, octahedral complexes **1** and **2**, yet derived from achiral tetradentate ligands, are chiral-at-metal complexes, capable of existing in two enantiomeric forms [19, 20]. Therefore, one should first rule out the possibility of spontaneous enantioenrichment of **1** and **2** upon preparation and by-crystallization isolation. To this end, specific rotation values of **1** and **2** were measured and found to be zero, as well as, predictably, that for complex **3** (SI, [Supplementary-material supplementary-material-1]).

**Figure 4:**
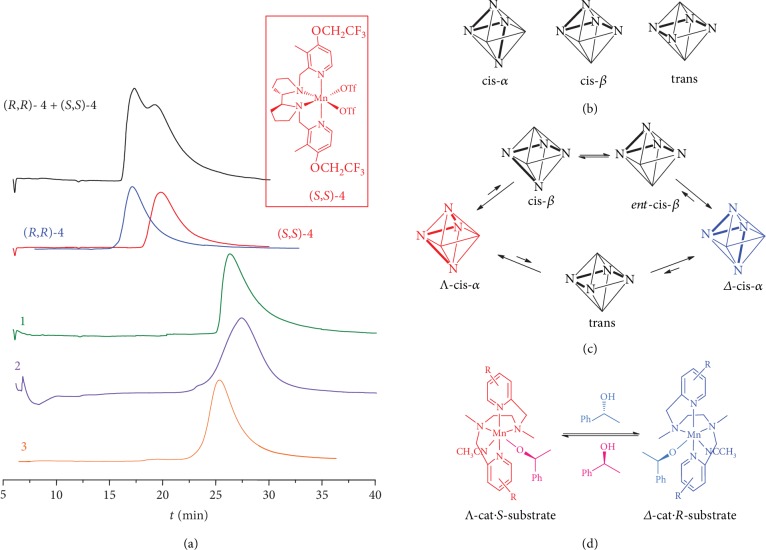
HPLC traces of (*R*,*R*)-**4** (blue), (*S*,*S*)-**4** (red), mixture (*R*,*R*)-**4** + (*S*,*S*)-**4** (black), **1** (green), **2** (violet), **3** (orange) (a). Possible topologies for octahedral iron complexes with *bis*-amine*-bis*-pyridine ligands (b). Proposed alternative pathways for the enantiomerization of complexes **1** and **2** (c). Predicted effect of enantiomers of 1-phenylethanol on the enantiomerization equilibrium (d).

The absence of optical rotation of the chiral-at-metal complexes **1** and **2** can indicate two alternative situations: (1) samples of **1** and **2** are conformationally stable and racemic and (2) the enantiomers of **1** and **2** are stereolabile, these complexes undergoing stereo isomerization leading to equilibrium racemic mixtures of the enantiomers. To distinguish between these possibilities, HPLC on chiral stationary phases (CSP) can be efficiently used [21–24]. To this end, HPLC conditions for the separation of enantiomeric chiral compexes (*R*,*R*)**-4** and (*S*,*S*)**-4** (Figure 4(a)) have been found: cellulose-based Chiralcel OJ-H CSP, eluent: hexane/ethanol 90 : 10, 0.1% trifluoroacetic acid, +20°C, flow rate 0.8 mL/min. Owing to the conformationally rigid bipyrrolidine backbone, the enantiomers of **4** are unable to stereo isomerize, which results in that a mixture of enantiomers gives a pair of separate, partially superimposed peaks (Figure 4(a)).

Contrariwise, the HPLC traces of complexes **1** and **2**, as well as of **3**, clearly exhibit single peaks (Figure 4(a)). While in the case of achiral **3**, such picture is predictable and unsurprising, the absence of two separate peaks from two enantiomers in the cases of **1** and **2** is highly informative, characteristic of dynamic enantiomerization of these compounds. In particular, the situation of fast enantiomerization, which is much faster than the chromatographic separation, is the case, leading to the coalescence of HPLC peaks of individual enantiomers into one signal [24].

The mechanism of this stereo isomerization is key issue, directly related to the mechanism of the emergence of dynamic control of the catalyst's chirality in the course of the catalytic reaction (see below). Possible explanation may be the following. In general, three coordination topologies, such as *cis-α*, *cis-β*, and *trans* (Figure 4(b)), have been documented for octahedral iron and manganese complexes with *bis*-amine*-bis*-pyridine type ligands [25–29]. In the solid state, complexes **1** (SI), **2** [29], **3** (SI), and **4** [30, 31] adopt *cis-α*-coordination topology. It is known, however, that octahedral metal triflate complexes with the same *bis*-amine*-bis*-pyridine ligand can exist in both *cis-α* and *cis-β* forms; these forms are stable in solution in case the chirality of the complex is predetermined by the rigid stereochemistry of the diamine backbone [26, 27].

The situation is different if the ligand contains conformationally nonrigid diamine bridge, like 1,2-ethylenediamine: the latter readily changes its conformation [32], which could entail the change of the overall ligand coordination topology in the complex. In polar media, such as aqueous acetonitrile (i.e., under catalytic conditions), as well as in ethanol-containing eluent under HPLC conditions, the triflates would be displaced from the labile *α*-coordination cites of Mn, preferentially existing in dissociated form, which is a prerequisite for the catalytic reaction to occur. At the same time, this would facilitate changing the coordination mode of the diamine-bridged chiral ligands of **1** and **2**. Apparently, a sequence of *cis-α*→*cis-β*, *ent*-*cis-β*→*cis-β*, *ent*-*cis-β*→*ent*-*cis-α* transitions, each one proceeding via consecutive dissociation/coordination of one of the pyridine moieties, could account for the plausible pathway for enantiomerization in complexes **1** and **2** (Figure 4(c)). At the same time, the hypothetic pathway through the square planar *trans*-intermediate could not *a priori* be ruled out, yet being less likely because of the need for the simultaneous dissociation of both pyridine moieties of the ligand (Figure 4(c)).

So, manganese aminopyridine complexes bearing tetradentate achiral, ethylenediamine-based ligands appear to be conformationally nonrigid likewise manganese salen [32, 33] and ruthenium salen [34] prototypes. In the cases of the cited “achiral” manganese and ruthenium salen-based catalysts, the addition of a coordinating chiral additive resulted in the emergence of asymmetric induction in oxidation and cyclopropanation reactions [32–34]. This was explained by translation of the chirality from the externally added chiral auxiliary toward the chirality-at-metal through shifting the equilibrium between the enantiomeric conformational isomers via formation of diastereomeric catalytic species. This phenomenon is regarded as *chiral environment amplification* or *dynamic chirality control* [32–37]. In much the same way, dynamic chirality control was more recently reported for complexes of manganese with nonrigid achiral *bis*-amine*-bis*-pyridine ligands: in the presence of Boc-protected *L*-proline as the chirality source, the dynamically racemic complexes catalyzed the asymmetric epoxidation of olefins in up to 60% *ee* [29]. The unique peculiarity of the present case, however, is that the role of the chiral auxiliary, ensuring the dynamic control of the catalyst chirality (Figure 4(d), bottom), is played *by the substrate itself*.

The absence of *ee* growth in the oxidation of alkyl mandelates on catalyst **1** (entries 10 and 11 of Table 1) apparently reflects their inability (owing to relatively low electron-donor properties) to stabilize the mirror enantioconformers of **2** via formation of diastereomeric coordination complexes [32–37]. In the case of **3**, in turn, the key condition for achieving stereoselectivity—the possibility of shifting the equilibrium between two mirror conformers of the catalyst—cannot be fulfilled in principle because of the absence of enantiomeric conformations for this complex.

### 2.3. Model Kinetic Scheme

**Figure 5:**
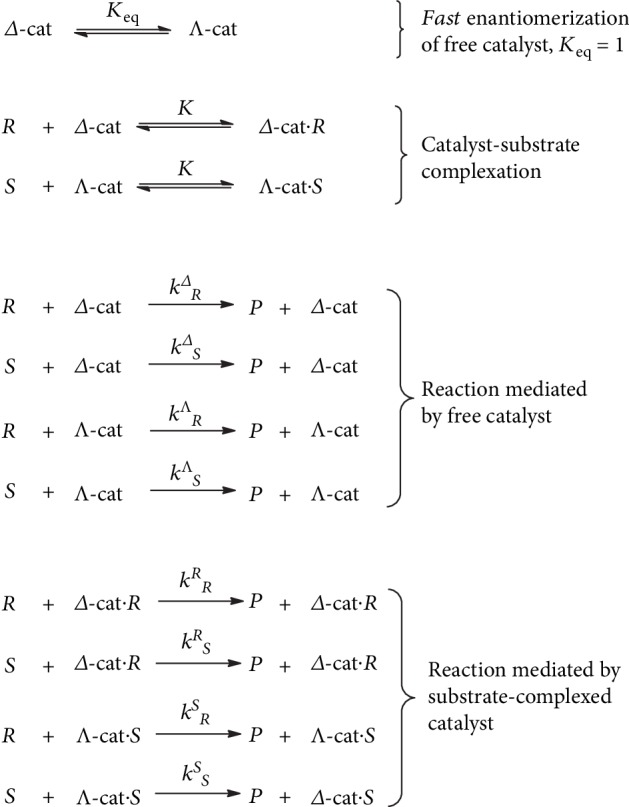
Proposed scheme of kinetic resolution with dynamically racemic catalyst. *P* is the product, *Λ*-cat and *Δ*-cat are the enantioconformations of the catalyst, and *R* and *S* stand for the substrate enantiomers.

The proposed reaction scheme is presented in Scheme S1, SI. For satisfactory quantitative description of the reactions in the system, the following minimal kinetic model can be considered (Figure 5). Based on DFT calculations data (SI), only the existence of [*Δ*-Cat·*R*] and [*Λ*-Cat·*S*] adducts should be taken into account, while formation of [*Λ*-Cat·*R*] and [*Δ*-Cat·*S*] adducts could be neglected as a first approximation.

Assuming the enantiomerization of the free catalyst to be the fastest reaction of all, one can obtain (see SI for details):

(1) $$\begin{array}{c} \frac{d\left[S\right]}{d\left[R\right]}=\frac{k\left[S\right]+{{Kk}^{R}}_{S}\left[R\right]\left[S\right]+{{Kk}^{S}}_{S}\left[S\right]\left[S\right]}{k\left[R\right]+{{Kk}^{S}}_{R}\left[S\right]\left[R\right]+{{Kk}^{R}}_{R}\left[R\right]\left[R\right]}, \end{array}$$

where *k* = *k*^Δ^_*R*_ + *k*^*Λ*^_*R*_ = *k*^Δ^_*S*_ + *k*^*Λ*^_*S*_ (SI). Applying evident relationships *k*^*S*^_*R*_ = *k*^*R*^_*S*_ and *k*^*S*^_*S*_ = *k*^*R*^_*R*_, and replacing *s* = [*S*]/*S*_0_ and *r* = [*R*]/*R*_0_ (*R*_0_ and *S*_0_ are the initial concentrations of the substrate (*R*)- and (*S*)-enantiomers), equation (1) can be reduced (SI) to the following:

(2) $$\begin{array}{c} \frac{ds}{dr}=\frac{s+{AS}_{0}\left(R_{0}/S_{0}\right)sr+{BS}_{0}s^{2}}{r+{AS}_{0}sr+{BS}_{0}\left(R_{0}/S_{0}\right)r^{2}}, \end{array}$$

where *A* = *Kk*^*R*^_*S*_/*k* = *Kk*^*S*^_*R*_/*k* and *B* = *Kk*^*S*^_*S*_/*k* = *Kk*^*R*^_*R*_/*k*. Equation (2) includes the initial concentration *S*_0_ and initial enantiomeric ratio *R*_0_/*S*_0_, and two parameters, *A* and *B*, that can be fitted to the experimental curves (Figures 2 and 3). In Figures 2 and 3, the experimentally determined *ee* vs. conversion and *er* vs. conversion plots for entries 1-8 of Table 1 are presented, together with the theoretical dependencies according to equation (2). The values of parameters *A* and *B* resulting therefrom are given in the last two columns of Table 1. Based on the results of three separate experiments at different *ee*_0_ for each catalyst (entries 1-3 and 4-6, respectively), the following average parameter values were calculated (1) for **1**: *A* = 34.5 ± 0.5 M^−1^, *B* = 12.9 ± 0.8 M^−1^ and (2) for **2**: *A* = 62 ± 4, *B* = 30.6 ± 1.2 (standard deviations are given). The *ee* and *er* vs. conversion plots for entries 1-3 of Table 1, simulated using the average *A* and *B* values, can be found in [Supplementary-material supplementary-material-1], SI.

We notice that parameter *AS*_0_ reflects the dominance of the heterochiral catalytic reaction mediated by substrate-complexed catalyst over the reaction mediated by free catalyst, while the ratio *A*/*B* = *k*^*S*^_*R*_/*k*^*S*^_*S*_ = *k*^*R*^_*S*_/*k*^*R*^_*R*_ defines the relative rates of the heterochiral and homochiral oxidation reactions, mediated by the substrate-complexed catalyst. As compared with **1**, complex **2** shows somewhat higher *A* and *B*, ensuring higher degree of substrate-induced acceleration of the overall process; at the same time, the *A*/*B* ratio for **1** is higher than for **2** (2.7 vs. 2.0), thus witnessing better stereodiscrimination by the substrate-complexed catalyst **1**.

Upon increasing the concentration of the reaction mixture, the nonlinear amplification of *ee* and *er* with increasing conversion became steeper, and higher optical purities were achieved at comparable conversion levels ([Supplementary-material supplementary-material-1]; see also [Supplementary-material supplementary-material-1] for details). Increasing or decreasing the reaction temperature (250 and 275 K) resulted in a slight decrease of both the *A* and *B* values (see [Supplementary-material supplementary-material-1] and [Supplementary-material supplementary-material-1] for details), thus revealing 263 K as the optimal reaction temperature.

**Figure 6:**
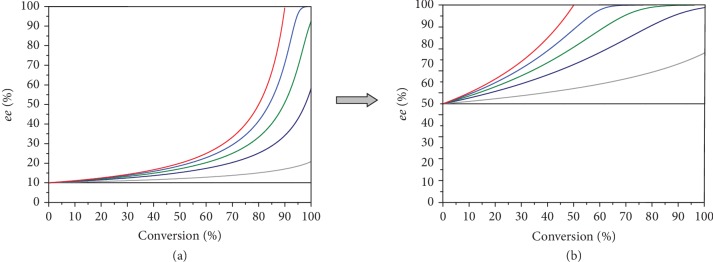
Theoretically predicted enantiomeric excess vs. conversion plots for at different initial *ee*s: *ee*_0_ = 10% (a), *ee*_0_ = 50% (b). *S*_0_ = 0.30 M, *B* = 10 M^−1^. *A*/*B* = 1 (black), 2 (grey), 5 (navy), 10 (green), 25 (blue), and 10^4^ M^−1^ (red).

The initial enantiomeric excess *ee*_0_ also dramatically affects the kinetic resolution. Indeed, the picture of *ee* evolution with conversion, calculated based on the scheme depicted in Figure 5, changes significantly when passing from the case with *ee*_0_ = 10% to the case with *ee*_0_ = 50% (Figure 6; see also SI, [Supplementary-material supplementary-material-1], B, C), predicting that even for moderate *A*/*B* ratios (2…5), the low *ee* of the starting precursor can be readily amplified up to nearly 100% in a sequence of kinetic resolutions. Relevant example is presented in entry 12 of Table 1 (see also Figures 2(c) and 2(f)), where enantiomeric purity of >80% *ee* was achieved starting from 45.5% initial *ee*_0_.

## 3. Conclusions

Overall, the above data demonstrate that nonracemic substrate mixtures can be kinetically resolved in a catalytic process, even though the catalyst's chirality is not predetermined by a chiral ligand: herewith, such process has been efficiently conducted by stereolabile manganese complexes with achiral tetradentate ligands, with the catalysts' chirality being dynamically determined by coordination of the chiral substrate to the Mn active sites. This is the first example of simultaneous action of two effects, chiral autoamplification [38] and dynamic chirality control (of the catalyst) [32–37], providing an unprecedented mechanism of chirality amplification in a far-from-equilibrium chemical system.

**Figure 7:**
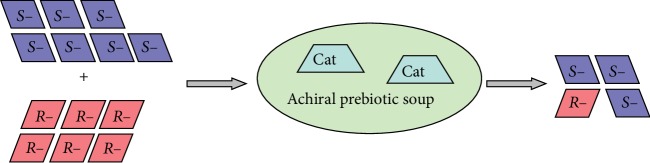
Proposed model of through-competition chirality amplification in the absence of exogenous sources of chirality. “*R*-” and “*S*-” are enantiomeric substrates; “Cat” stands for catalyst.

This reaction suggests an idea of alternative, nonautocatalytic [38] chemical mechanism of transformation of abiotic raw material with small initial enantiomeric imbalance [2, 8–14, 18, 39] into an enantiomerically pure specimen, without action of catalysts with predetermined chirality or participation of any other exogenous chiral molecules (Figure 7). In this system, the predominant enantiomer of the scalemic substrate itself controls the absolute configuration of the conformationally flexible catalyst, eventually “outcompeting” the other enantiomer in the concurrent catalyzed oxidation reaction. This through-competition “chemical evolution” mechanism, recreated here at the molecular level, has analogy with the natural selection-based biological evolution. Last but not least, the reported catalyzed self-enantioenrichment mechanism shows unprecedented capacity of operating efficiently and unidirectionally in partially aqueous medium [40], i.e., one step closer to the conditions modeling the prebiotic broth. Further studies of this interesting reaction are underway.

Least of all this first demonstration of the substrate-governed kinetic resolution on catalysts derived from achiral precursors claims to give exhaustive explanations, rather serving as a proof of new principle, which contributes to the discussion on the origins of homochirality on Earth by providing a so far missing piece of the overall puzzle. Hopefully, such fresh vision of the problem can help us to make another step towards the understanding of the origins of life and its fundamental principles.

## 4. Materials and Methods

### 4.1. Materials

For catalytic epoxidation experiments, 30% analytical grade aqueous H_2_O_2_ was used. Mn complexes **2** [21], **4** [30, 31], and (*S*)-1-phenylethanol [30] were prepared as described. All chemicals and solvents were from Sigma-Aldrich, Acros Organics, or Alfa Aesar commercial reagents (and were used without additional purification unless noted otherwise), or were prepared according to literature procedures.

### 4.2. Methods

^1^H and ^13^C NMR spectra were measured on Bruker Avance 400 at 400.13 and 100.613 MHz, respectively, or on Bruker DPX-250 at 250.13 and 62.903 MHz, respectively. Chemical shifts were internally referenced to tetramethylsilane. Specific rotation values (in CH_3_CN) were measured using Kruss Optronic polarimeter P8000-T using 100 mm cuvette.

Analytical chiral resolutions were performed by HPLC on Shimadzu LC-20 chromatograph equipped with chiral stationary phases Chiralcel/Chiralpak.

Experimental (kinetic) data were treated using Origin 9.0 package.

## Figures and Tables

**Table 1:** Kinetic resolution of scalemic 1-phenylethanol in the presence of Mn complexes **1-3**^a^.
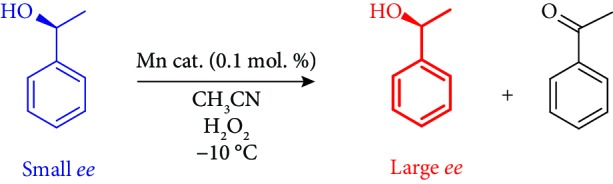

No.	Substrate	Initial *ee* (%)*^b^*	*S*_0_, M	Catalyst	Conversion (%)^b^	Final *ee* (%)^b^	Kinetic parameters	
							*A*, M^−1^	*B*, M^−1^
1	(*S*)-1-Ph-ethanol	8.3	0.29	**1**	98.4	26.0	34.5	13.8
2	(*S*)-1-Ph-ethanol	13.0	0.29	**1**	97.3	42.6	35.0	12.5
3	(*S*)-1-Ph-ethanol	15.7	0.29	**1**	99.6	50.0	34.0	12.5
4	(*S*)-1-Ph-ethanol	8.0	0.27	**2**	85.9	16.9	66.7	31.5
5	(*S*)-1-Ph-ethanol	11.4	0.29	**2**	93.6	25.1	62.1	31.0
6	(*S*)-1-Ph-ethanol	19.5	0.30	**2**	98.9	46.8	58.3	29.2
7	(*R*)-1-Ph-ethanol	19.3	0.32*^c^*	**1**	97.9	49.9	33.0	14.0
8	(*R*)-1-Ph-ethanol	19.5	0.31*^c^*	**2**	97.5	45.3	60.5	31.5
9	(*S*)-1-Ph-ethanol	12.8	0.29	**3**	69.5	12.6	—	—
10	(*R*)-Methyl mandelate	17.4	0.20 *^c^*	**1**	26.3	17.5	—	—
11	(*R*)-Butyl mandelate	14.6	0.22 *^c^*	**1**	52.8	14.9	—	—
12	(*S*)-1-Ph-ethanol	45.5	0.36	**1**	95.7	81.2	32.5	13.7

^a^For reaction conditions, see SI. ^b^Determined by chiral HPLC. ^c^Initial concentration of (*R*)-enantiomer.

## Supplementary Materials

Supplementary Materials Supplementary materials such as detailed description of materials used; methods; synthetic, analytical, and catalytic procedures; crystal data for the new complexes **1** and **3**; treatment of the kinetic scheme presented in Figure 5; DFT calculation details; additional tables and figures related to this article; and HPLC traces can be found, in the online version, at doi:10.34133/2019/4756025. X-ray crystallographic data in CIF for CCDC 1887476 (**1**) and CCDC 1887477 (**3**) can be obtained free of charge from the Cambridge Crystallographic Data Centre via ww.ccdc.cam.ac.uk/data_request/cif and from the authors.
