# Supplementary material for: Chiral Autoamplification Meets Dynamic Chirality Control to Suggest Nonautocatalytic Chemical Model of Prebiotic Chirality Amplification
Source: Research (Wash D C). 2019 Nov 4;2019:4756025. doi: 10.34133/2019/4756025 (PMC6946252; doi:10.34133/2019/4756025)
Supplement: Supplementary Materials — such as detailed description of materials used; methods; synthetic, analytical, and catalytic procedures; crystal data for the new complexes 1 and 3; treatment of the kinetic scheme presented in Figure 5; DFT calculation details; additional tables and figures related to this article; and HPLC traces can be found, in the online version, at doi:10.34133/2019/4756025. X-ray crystallographic data in CIF for CCDC 1887476 (1) and CCDC 1887477 (3) can be obtained free of charge from the Cambridge Crystallographic Data Centre via ww.ccdc.cam.ac.uk/data_request/cif and from the authors. [file 4756025.f1.pdf]

Chiral Autoamplification Meets Dynamic Chirality Control to Suggest Non-Autocatalytic Chemical Model of Prebiotic Chirality Amplification

*Evgenii P. Talsi,[a,b] Anna A. Bryliakova,[b] Roman V. Ottenbacher,[a,b] Tatyana V. Rybalova[a,c] and Konstantin P. Bryliakov[a,b]**

[a] Novosibirsk State University, Pirogova 2, Novosibirsk 630090, Russian Federation

[b] Boreskov Institute of Catalysis, Pr. Lavrentieva 5, Novosibirsk 630090, Russian Federation

[c] Vorozhtsov Novosibirsk Institute of Organic Chemistry, Pr. Lavrentieva 9, Novosibirsk 630090, Russian Federation

E-mail: bryliako@catalysis.ru

Supporting Information

Materials and methods 2

Syntheses of Mn complexes 3

Kinetic resolution procedures 4

Schemes S1 and S2; treatment of the kinetic scheme 6

Figure S1 9

Effect of concentration on the nonlinear *ee* growth (Table S1, Figure S2) 10

Effect of temperature on the nonlinear *ee* growth (Table S2, Figure S3) 11

Figures S4, Table S3 12

Figure S5 13

Figure S6 14

X-ray crystallography 15

DFT calculations 21

Additional references 23

Copies of NMR spectra 24

HPLC traces 26

DFT optimized Cartesian coordinates of Mn complexes 50

Materials

For catalytic epoxidation experiments, 30 % analytical grade aqueous H_2O_2 was used. Mn complexes **2**,[21] **4**,[30,31] and (*S*)-1-phenylethanol[30] were prepared as described. All chemicals and solvents were either Aldrich, Acros Organics, or Alfa Aesar commercial reagents (and were used without additional purification unless noted otherwise), or were prepared according to literature procedures.

Methods

^{1}H and ^{13}C NMR spectra were measured on Bruker Avance 400 at 400.13 and 100.613 MHz, respectively, or on Bruker DPX-250 at 250.13 and 62.903 MHz, respectively. Chemical shifts were internally referenced to tetramethylsilane. Specific rotation values (in CH_3CN) were measured using Kruss Optronic polarimeter P8000-T using 100 mm cuvette.

Reaction mixtures were analyzed by HPLC (Shimadzu LC-20) with chiral stationary phase Chiralcel OD-H, to obtain the relative concentrations of the 1-phenylethanol and acetophenone, and the enantiomeric excess of the 1-arylalkanol (see table below). Experimental uncertainty of enantioselectivity measurements did not exceed ±0.5% *ee*.

Mn complexes (*R*,*R*)-**4**, (*S*,*S*)-**4**, **1**, **2**, and **3** were resolved by HPLC (Shimadzu LC-20) with chiral stationary phase Chiralpak OJ-H (eluent: hexane/ethanol 90:10; 0.1 % trifluoroacetic acid), 20 °C, 0.8 mL/min, detection at 206 nm.

Experimental (kinetic) data were treated using Origin 9.0 package.

HPLC separation details

Analyte	Column	T °C	Eluent:	λ, nm	V, mL/min	t_{R1}, min (config)	t_{R2}, min (config)
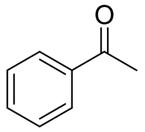	Chiralcel OD-H	20	*i*-PrOH/ hexane 3:97	206	1.0	6.4 (−)	
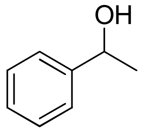	Chiralcel OD-H	20	*i*-PrOH/ hexane 3:97	206	1.0	12.0 (*R*)	14.4 (*S*)

(*S*,*S*)-**4**	Chiralcel OJ-H	20	EtOH/ hexane 10:90 [a]	206	0.8	16.8
(*R*,*R*)-**4**	Chiralcel OJ-H	20	EtOH/ hexane 10:90 [a]	206	0.8	19.8
**1**	Chiralcel OJ-H	20	EtOH/ hexane 10:90 [a]	206	0.8	26.2
**2**	Chiralcel OJ-H	20	EtOH/ hexane 10:90 [a]	206	0.8	27.3
**3**	Chiralcel OJ-H	20	EtOH/ hexane 10:90 [a]	206	0.8	25.4

[a] 0.1 % vol. trifluoroacetic acid was added.

Syntheses of Mn complexes

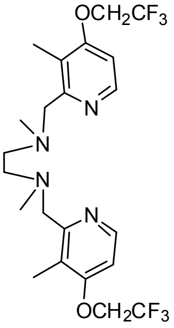

***N*[1],*N*[2]-dimethyl-*N*1,*N*2-bis((3-methyl-4-(2,2,2-trifluoroethoxy)pyridin-2-yl)methyl)ethane-1,2-diamine**. To 2-(chloromethyl)-3-methyl-4-(2,2,2-trifluoroethoxy)pyridine hydrochloride (303.7 mg, 1.1 mmol) and *N*[1],*N*[2]-dimethylethane-1,2-diamine (54 μL, 0.50 mmol), water (1.0 mL) and CH_2Cl_2 (1.5 mL) were added, and NaOH (88 mg, 2.2 mmol) was added in one portion. The mixture was stirred overnight at r. t., diluted with water (2 mL), and extracted with CH_2Cl_2 (3×5 mL). Combined organic extracts were washed with aqueous NaCl, dried with $CaSO_4$, and evaporated under reduced pressure. The crude product was purified on a short SiO_2 column (eluent: acetone; acetone:MeOH 5:1), to yield the desired product (173 mg, 70 %).

^{1}H NMR ($CDCl_3$/CCl_4, 25 °C), δ: 8.26 (d, 2H, Ar-*H*, $^3J_{HH}$ = 5.5 Hz); 6.58 (d, 2H, Ar-*H*, $^3J_{HH}$ = 5.5 Hz); 4.34 (q, 4H, OCH_2CF_3, $^3J_{HF}$ = 7.9 Hz); 3.63 (s, 4H, ArCH_2N); 2.61 (s, 4H, C_2H_4), 2.23 (s, 6H, ArCH_3); 2.20 (s, 6H, NCH_3).

^{13}C NMR{^{1}H} ($CDCl_3/CCl_4$, 25 °C), δ: 161.58 (2C, C); 158.55 (2C, C); 147.29 (2C, CH); 123.05 (q, 2C, CF_3, $^{1}J_{HF}$ = 278 Hz); 122.44 (2C, C); 105.22 (2C, CH); 65.41 (q, 2C, CH_2CF_3, $^{2}J_{HF}$ = 36 Hz); 62.34 (2C, ArCH_2N); 55.48 (2C, C_2H_4); 42.66 (2C, NCH_3); 10.38 (2C, ArCH_3).

Complex 1

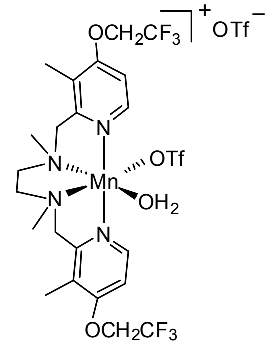

To N^1,N^2-dimethyl-*N*1,*N*2-bis((3-methyl-4-(2,2,2-trifluoroethoxy)pyridin-2-yl)methyl)ethane-1,2-diamine (150 mg, 0.30 mmol) in acetonitrile (1.5 mL), $Mn(OTf)_2 \cdot CH_3CN$ (118 mg, 0.30 mmol) was added in one portion, and the mixture was stirred overnight. The mixture was filtered, and diethyl ether (10 mL) was layered upon, to afford, in a few days, colorless crystals of **1** (132.5 mg).

Complex 3

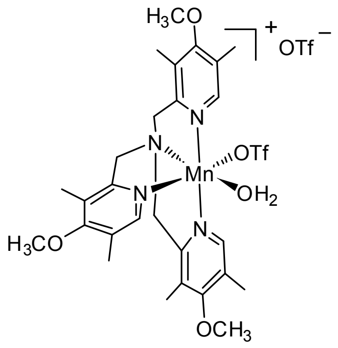

To tris((4-methoxy-3,5-dimethylpyridin-2-yl)methyl)amine (66 mg, 0.142 mmol) in acetonitrile (0.8 mL), $Mn(OTf)_2 \cdot CH_3CN$ (59 mg, 0.15 mmol) was added in one portion, and the mixture was stirred for 1 h, followed by filtering, layering with diethyl ether (6 mL), and storing at +4 °C for several days. Second recrystallization from CH_3CN/Et_2O. afforded X-ray quality colorless needle crystals of **3** (71 mg).

Kinetic resolution procedures

Kinetic resolution of scalemic 1-phenylethanol

Scelemic 1-phenylethanol mixtures were prepared by mixing calculated volumes of racemic and non-racemic 1-phenylethanol (either homemade 92 % *ee* (*S*)-1-phenylethanol or commercial (*R*)-1-

phenylethanol, >99.0 % *ee*, SigmaAldrich). The resulting mixture with total amount of 1-phenylethanol of 0.18…0.23 mmol and of appropriate manganese complex (0.2 μmol) in CH_3CN (0.40 mL; or 0.8 or 0.2 mL in the case of experiments presented in Figure 2, red, and green curves, respectively) was prepared and analyzed by chiral HPLC (twice) to obtain the initial enantiomeric imbalance (ee_0). The mixture was thermostated at -10 °C, and 30 % aqueous H_2O_2 was added in appropriate portions (typically 2-5 μL every 0.5 h. At the end of each 0.5 h period, 2.5 μL aliquot was taken, dissolved in 0.25 mL of *i*-propanol, and analyzed by chiral HPLC to obtain the relative concentrations of the alcohol and the ketone, and the alcohol *ee* at several different conversions. The results were presented in the form of *ee* vs. conversion and [S]/[R] vs. conversion plots.

Kinetic resolution of scalemic alkyl mandelates

The solution of the appropriate racemic alkyl mandelate and enantiopure (*R*)-alkyl mandelate (total amount of alkyl mandelate 0.2 mmol) and of appropriate manganese complex (0.2 μmol) in CH_3CN (0.40 mL) was prepared and analyzed by chiral HPLC (twice) to obtain the initial enantiomeric imbalance (ee_0). The mixture was thermostated at -10 °C, and 30 % aqueous H_2O_2 was added in appropriate portions (typically 2-5 μL every 0.5 h. At the end of each 0.5 h period, 2.5 μL aliquot was taken, dissolved in 0.25 mL of *i*-propanol, and analyzed by chiral HPLC to obtain the relative concentrations of the alcohol and the ketone, and the alcohol *ee* at several different conversions. No increase in the *ee* was observed over the reaction course.

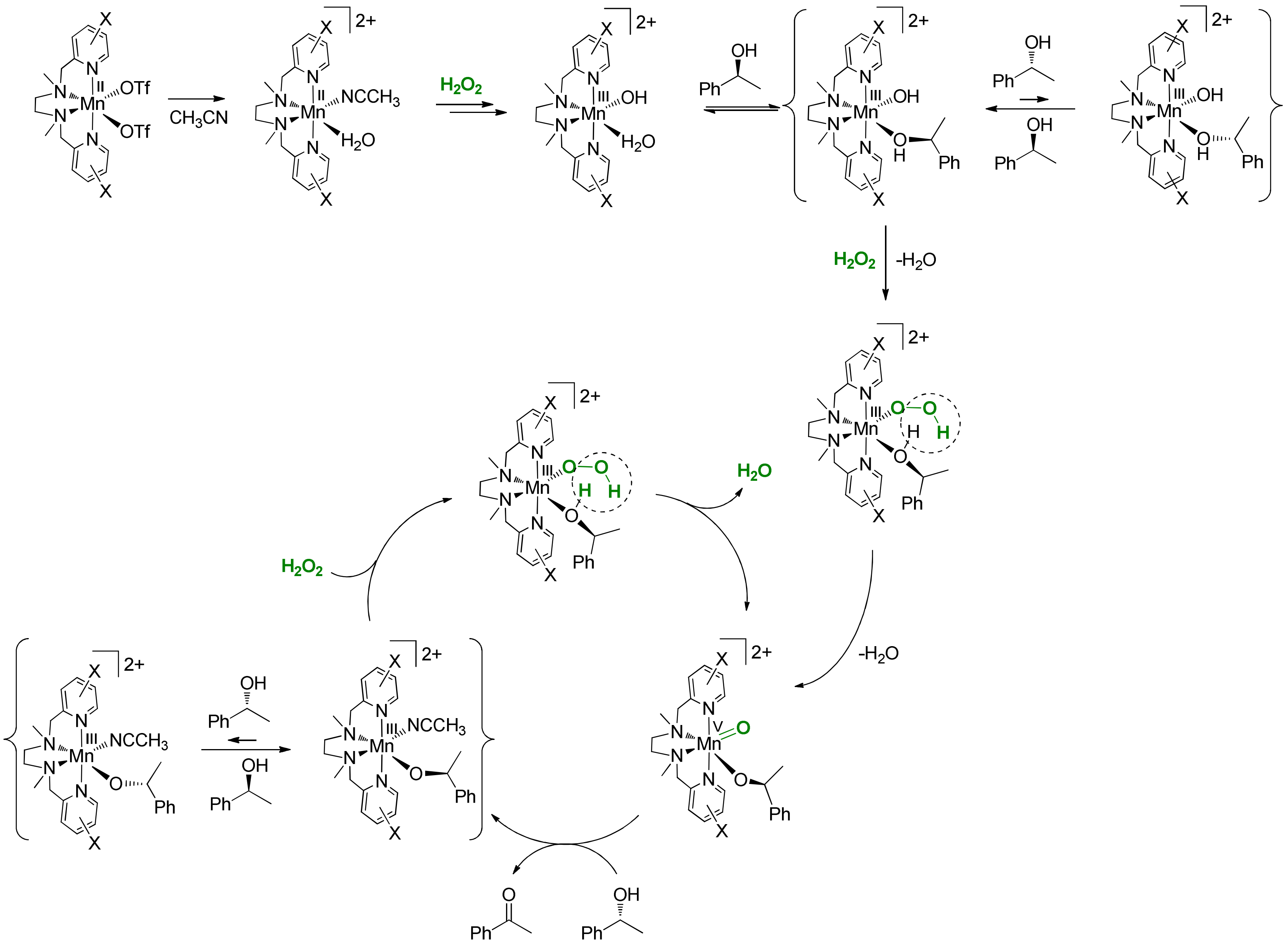

Scheme S1. Proposed reaction scheme of 1-phenylethanol oxidation, mediated by dynamically racemic Mn catalysts.

Treatment of the kinetic scheme

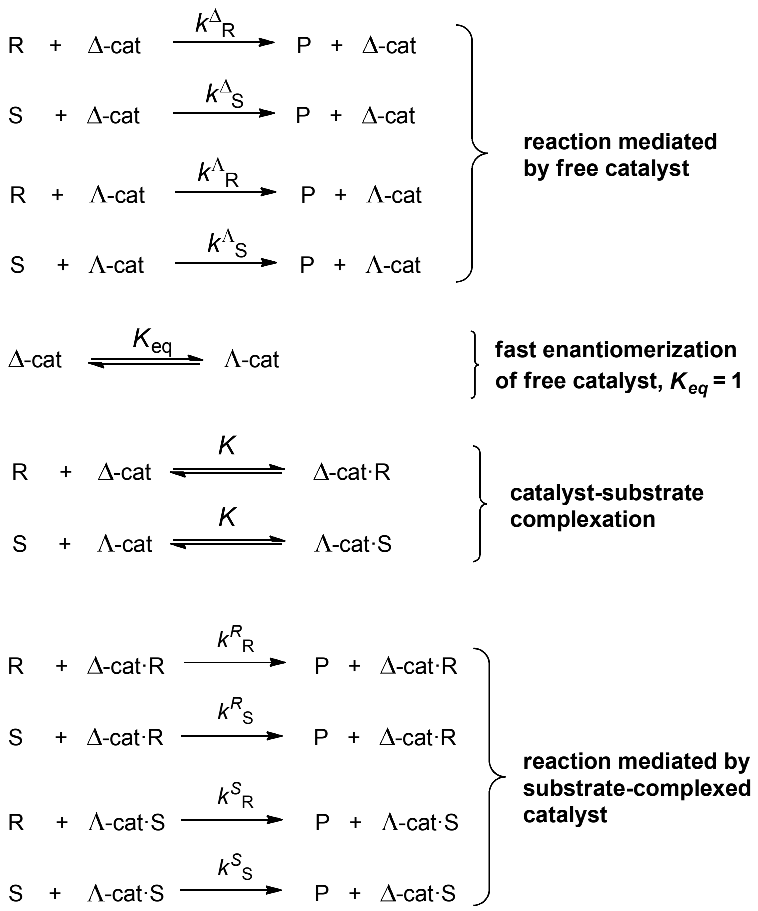

Scheme S2. Model kinetic scheme of kinetic resolution with dynamically racemic catalyst. P is product, cat is catalyst, R and S stand for the enantiomers of the chiral substrate.

In Scheme S2, formation of catalyst-substrate adducts [Δ-Cat·S] and [Λ-Cat·R] has been neglected, assuming that stability constants for such adducts should be << *K*. DFT calculations (Table S8, SI) provide qualitative support of this assumption.

The overall rates of consumption of the (*S*)- and (*R*)-alcohol, respectively, are

$$\frac{d[\mathrm{S}]}{dt} = -k^{\Delta}[\Delta - \mathrm{cat}][\mathrm{S}] - k^{\Lambda}[\Lambda - \mathrm{cat}][\mathrm{S}] - k^{R}{}_{S}[\Delta - \mathrm{cat}\cdot\mathrm{R}][\mathrm{S}] - k^{S}{}_{S}[\Lambda - \mathrm{cat}\cdot\mathrm{S}][\mathrm{S}] \qquad \text{(S1)}$$

$$\frac{d[\mathrm{R}]}{dt} = -k^{\Delta}[\Delta - \mathrm{cat}][\mathrm{R}] - -k^{\Lambda}[\Lambda - \mathrm{cat}][\mathrm{R}] - k^{R}{}_{R}[\Delta - \mathrm{cat}\cdot\mathrm{R}][\mathrm{R}] - k^{S}{}_{R}[\Lambda - \mathrm{cat}\cdot\mathrm{S}][\mathrm{R}] \qquad \text{(S2)}$$

Assuming that the enantiomerization equilibrium of the free catalyst is the fastest reaction in Scheme S1 (i.e. [Δ-Cat] = [Λ-Cat] = [X]), and taking into account the catalyst-substrate complexation equilibria and the mass balance for catalyst Cat_0 = [Δ-Cat] + [Λ-Cat] + [Δ-Cat·R] + [Λ-Cat·S], one can calculate the concentrations of all forms of the catalyst:

$$[\mathrm{cat}] = \frac{\mathrm{cat}_0}{2+K[\mathrm{R}]+K[\mathrm{S}]},\ [\mathrm{cat}\cdot\mathrm{S}] = \frac{\mathrm{cat}_0 K[\Lambda-\mathrm{cat}][\mathrm{S}]}{2+K[\mathrm{R}]+K[\mathrm{S}]},\ [\mathrm{cat}\cdot\mathrm{R}] = \frac{\mathrm{cat}_0 K[\Delta-\mathrm{cat}][\mathrm{R}]}{2+K[\mathrm{R}]+K[\mathrm{S}]}\ , \qquad \text{(S3)}$$

We notice the following self-evident relationships:

$$-k^{\Delta}{}_{R}[\Delta-\mathrm{cat}][\mathrm{R}] - k^{\Lambda}{}_{R}[\Lambda-\mathrm{cat}][\mathrm{R}] = -k^{\Delta}{}_{R}[\mathrm{X}][\mathrm{R}] - k^{\Lambda}{}_{R}[\mathrm{X}][\mathrm{R}] = -k[\mathrm{X}][\mathrm{R}] \qquad \text{(S4)}$$

$$-k^{\Delta}{}_{S}[\Delta-\mathrm{cat}][\mathrm{S}] - k^{\Lambda}{}_{S}[\Lambda-\mathrm{cat}][\mathrm{S}] = -k^{\Delta}{}_{R}[\mathrm{X}][\mathrm{S}] - k^{\Lambda}{}_{R}[\mathrm{X}][\mathrm{S}] = -k[\mathrm{X}][\mathrm{S}] \qquad \text{(S5)}$$

which in combination with eq. (S1), (S2) and (S3) leads to the following equation

$$\frac{d[\mathrm{S}]}{d[\mathrm{R}]} = \frac{k[\mathrm{S}] + Kk^{R}{}_{S}[\mathrm{R}][\mathrm{S}] + Kk^{S}{}_{S}[\mathrm{S}][\mathrm{S}]}{k[\mathrm{R}] + Kk^{S}{}_{R}[\mathrm{S}][\mathrm{R}] + Kk^{R}{}_{R}[\mathrm{R}][\mathrm{R}]} \qquad \text{(S6)}$$

Taking into account that, evidently, $k^{S}{}_{R} = k^{R}{}_{S}$ and $k^{S}{}_{S} = k^{R}{}_{R}$, multiplying left and right parts of (S6) by R_0/S_0 (R_0 and S_0 are the initial concentrations of the (*R*)- and (*S*)-enantiomers), substituting $\frac{Kk^{R}{}_{S}}{k} = \frac{Kk^{S}{}_{R}}{k} = A$, and $\frac{Kk^{S}{}_{S}}{k} = \frac{Kk^{R}{}_{R}}{k} = B$, and introducing dimensionless concentrations $s = [\mathrm{S}]/S_0$ and $r = [\mathrm{R}]/R_0$, one obtains

$$\frac{ds}{dr} = \frac{s + AS_0\frac{R_0}{S_0}sr + BS_0s^2}{r + AS_0sr + BS_0\frac{R_0}{S_0}r^2} \qquad \text{(S5)}$$

R_0/S_0 for each experiment is known (and equal to $\frac{R_0}{S_0} = \frac{1-ee_0}{1+ee_0}$, where ee_0 is expressed as a decimal fraction). In effect, this equation can be solved numerically to yield $s = F(A, B, r)$. Parameters A and B can be revealed by fitting the *ee* vs. conversion or, better, *er* vs. conversion

dependencies to the experimental points. For this purpose, the following obvious relationships have been used:

$$er = \frac{[S]}{[R]} = \frac{s}{r}\left(\frac{1+ee_0}{1-ee_0}\right);$$

$$conversion = \frac{S_0 + R_0 - ([S]+[R])}{S_0 + R_0} = \frac{2 - r(1-ee_0) - s(1+ee_0)}{2};$$

$$ee = \frac{[S]-[R]}{[S]+[R]} = \frac{s - r\left(\frac{1-ee_0}{1+ee_0}\right)}{s + r\left(\frac{1-ee_0}{1+ee_0}\right)}.$$

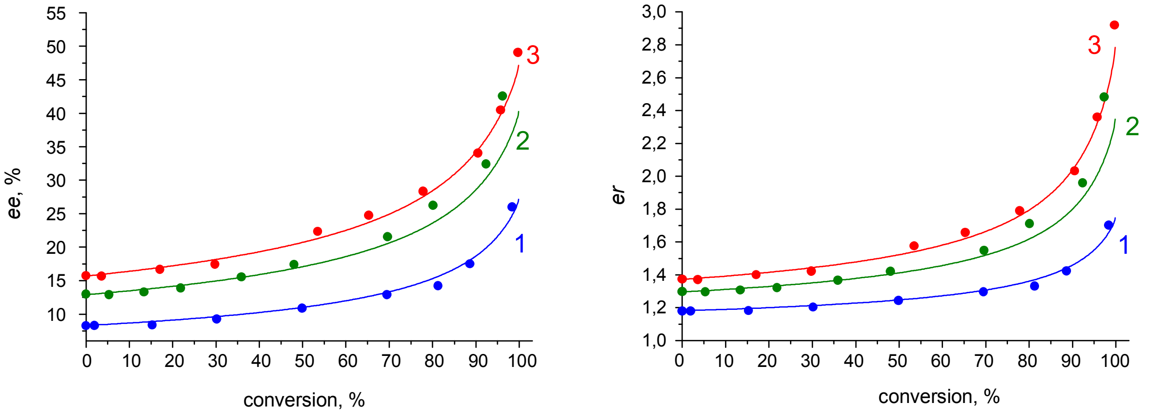

Figure S1. Enantiomeric excess (*ee*) vs. conversion (A) plot and [S]/[R] ratio (*er*) vs. conversion plot (B) for the kinetic resolution of scalemic 1-phenylethanol in the presence of achiral catalyst **1**. Dots represent experimental data; solid lines are theoretical fits with averaged parameters $A = 34.5$ M^{-1}, $B = 12.9$ M^{-1}. Curves are numbered in accordance with the numbers of entries in Table 1.

Effect of concentration on the nonlinear *ee* growth

Table S1. Kinetic resolution of scalemic 1-phenylethanol in the presence of Mn complexes at different initial concentrations of the substrate. [a]

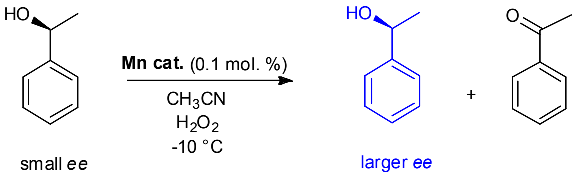

No	substrate	initial *ee* (%)[b]	S_0, M	catalyst	conversion (%)[b]	final *ee* (%)[b]	Modeling results *A*, M^{-1}	Modeling results *B*, M^{-1}
1	1-Ph-ethanol	22.5	0.15	**1**	98.0	46.0	33.0	13.9
2	1-Ph-ethanol	23.3	0.25	**1**	97.3	48.4	33.0	13.5
3	1-Ph-ethanol	24.5	0.53	**1**	96.5	51.7	33.0	14.8

[a] Reaction conditions as reported in page 4 of the SI. [b] Determined by chiral HPLC (see above).

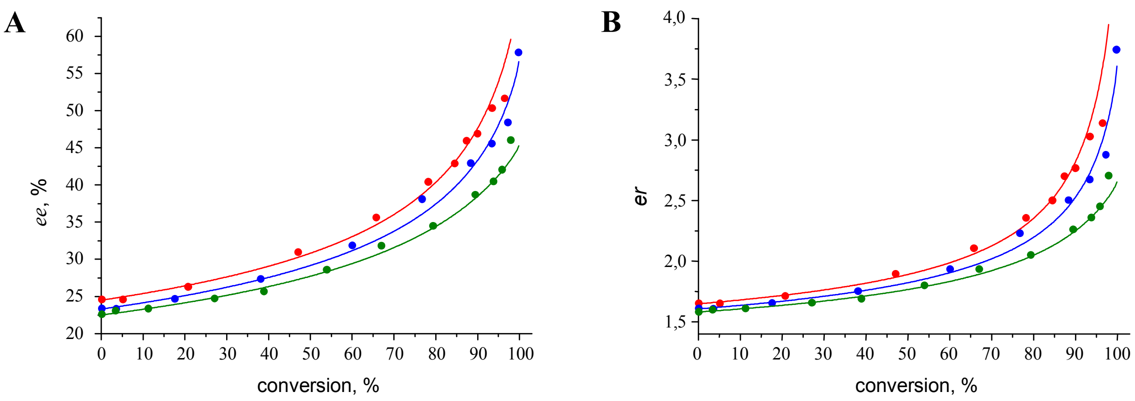

Figure S2. Enantiomeric excess (A) and enantiomeric ratio (B) vs. conversion plots for the kinetic resolution of scalemic 1-phenylethanol in the presence of achiral catalyst **1**, at different initial substrate concentrations. Dots represent experimental data; solid lines are theoretical curves. Red: S_0 = 0.53 M^{-1}, ee_0 = 24.5 %; blue: S_0 = 0.25 M, ee_0 = 23.3 %; green: S_0 = 0.15 M, ee_0 = 22.5 %.

Effect of temperature on the nonlinear *ee* growth

Table S2. Kinetic resolution of scalemic 1-phenylethanol in the presence of Mn complexes at different initial concentrations of the substrate.[a]

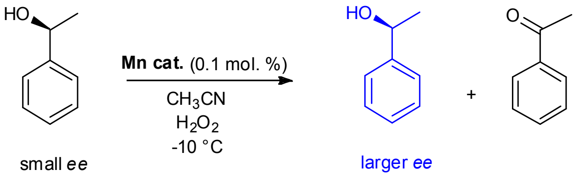

No	substrate	T, K	initial *ee* (%)[b]	S_0, M	catalyst	Modeling results	
						A, M^{-1}	B, M^{-1}
1	1-Ph-ethanol	**250**	14.9	0.29	**1**	29.8	11.6
2	1-Ph-ethanol	**263**	15.7	0.29	**1**	34.5 [c]	12.9[c]
3	1-Ph-ethanol	**275**	14.2	0.31	**1**	31.9	11.5

[a] Reaction conditions as reported in page 4 of the SI. [b] Determined by chiral HPLC as reported (refs. 2a,b). [a] Average A and B values (A = 34.5±0.5 M^{-1}, B = 12.9±0.8 M^{-1}, see main text).

The corresponding *ee* vs. conversion and *er* vs. conversion curves are presented in Figure S2.

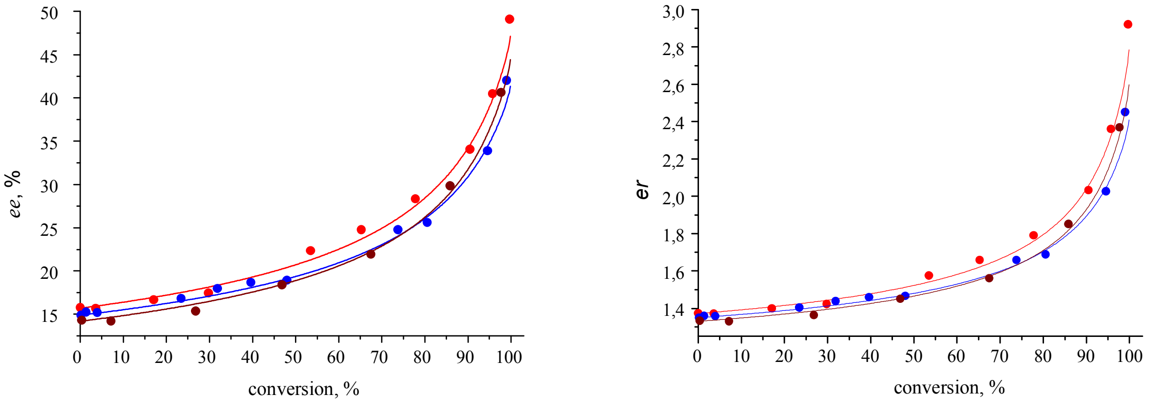

Figure S3. Enantiomeric excess (*ee*) vs. conversion (A) and enantiomeric ratio (*er*) vs. conversion (B) plots for the kinetic resolution of scalemic 1-phenylethanol in the presence of achiral catalyst **1**, at different temperatures. Dots represent experimental data; solid lines are theoretical fits. Blue: T = 250 K; S_0 = 0.27 M^{-1}, ee_0 = 0.149; Red: T = 263 K; S_0 = 0.29 M^{-1}, ee_0 = 0.157; Wine: T = 275 K; S_0 = 0.27 M^{-1}, ee_0 = 0.142.

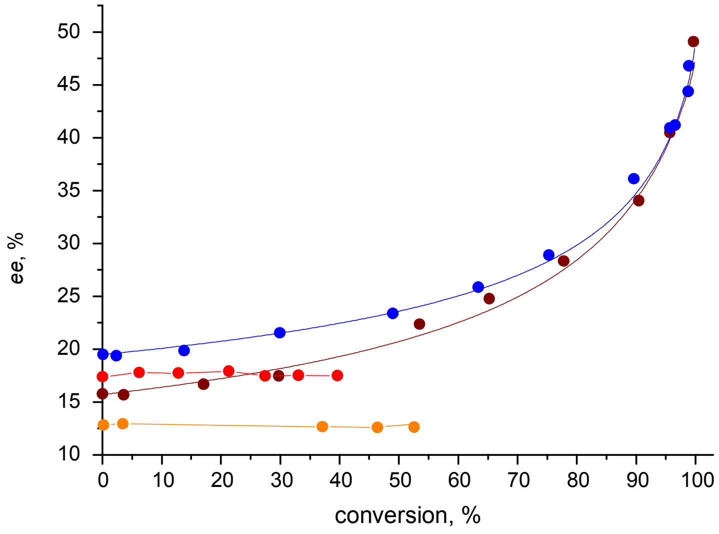

Figure S4. Enantiomeric excess (*ee*) vs. conversion plots for the kinetic resolution of
– (*S*)-1-phenylethanol in the presence of achiral catalyst **1** (wine, ee_0 = 15.7%; entry 3 of Table 1);
– (*S*)-1-phenylethanol in the presence of achiral catalyst **2** (blue, ee_0 = 19.4%; entry 6 of Table 1);
– (*S*)-1-phenylethanol in the presence of achiral catalyst **3** (orange, ee_0 = 12.8%; entry 7 of Table 1);
– (*R*)-methyl mandelate in the presence of achiral catalyst **1** (red, ee_0 = 17.4%; entry 8 of Table 1).

Table S3. Optical rotation values of complexes **1**, **2**, **3**, **4** (values averaged over 7 measurements for each complex).

Complex	C	Solvent	$[\alpha]_D^{20}$	Comment
**1**	1.19	CH_3CN	+0.16 °	
**2**	1.42	CH_3CN	+0.14 °	
**3**	2.06	CH_3CN	+0.18 °	*a fortiori* achiral complex
**4**	1.54	CH_3CN	–60.35 °	(*S*,*S*)-enantiomer

In the cases of **1**, **2**, **3**, the specific rotation values fell within the experimental uncertainty (±0.2 °); cf. -60.4 ° for the enantiomerically pure chiral complex **4**. This clearly shows that chiral-at-metal complexes **1** and **2** are not rotating, like the achiral complex **3**.

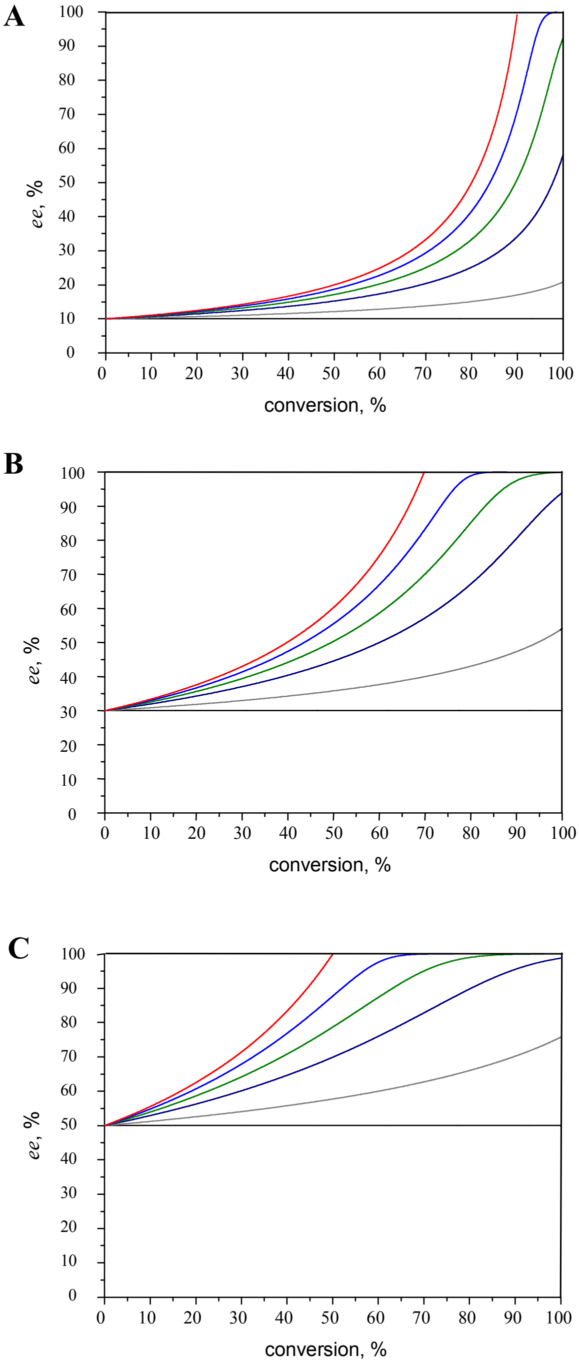

Figure S5. Theoretically predicted enantiomeric excess vs. conversion plots for at different initial *ee*s: ee_0 = 10 % (A), ee_0 = 30 % (B), ee_0 = 50 % (C). S_0 = 0.30 M, B = 10 M^{-1}. A/B = 1 (black), 2 (grey), 5 (navy), 10 (green), 25 (blue), 10^4 M^{-1} (red).

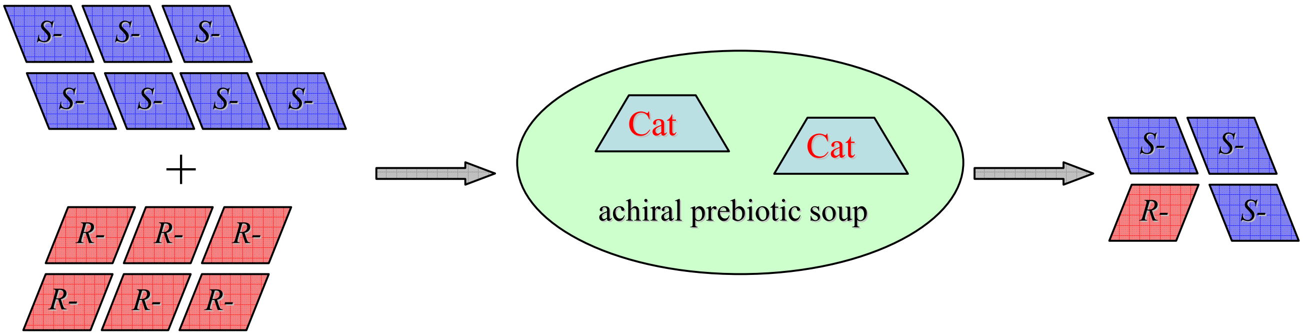

Figure S6. Proposed model of through-competition chirality amplification in the absence of exogenous sources of chirality. "*R*-" and "*S*-" are enantiomeric substrates, "Cat" stands for catalyst.

X-ray crystallography

X-ray data for complex 1 and 3

Single-crystal diffraction data for **1** and **3** were collected at room temperature on a Bruker Kappa Apex II CCD diffractometer using *φ, ω* scans of narrow (0.5°) frames with Mo Kα radiation (λ = 0.71073 Å) and a graphite monochromator. The structures were solved by direct methods and refined by full-matrix least-squares method against all F^2 in anisotropic (isotropic for H) approximation using the *SHELX-97* programs set.[S1] Absorption corrections were applied based on intensities of equivalent reflections using *SADABS* programs.[S2] The hydrogen atoms positions were calculated geometrically and refined in riding model exept the water hydrogens refined independently with restriction of O-H bond length of 0.83 Å. Free solvent accessible volume for compound **2** derived from PLATON[S3,S4] routine analysis was found to be 11.6% (449.8 $Å^3$). This volume is occupied by highly disordered solvent molecules that could not be modeled as a set of discrete atomic sites. We employed PLATON/SQUEEZE procedure to calculate the contribution to the diffraction from the solvent region and thereby produced a set of solvent-free diffraction intensities. The most probable solvents can be H_2O and/or acetonitryl. Asymmetric unit of **5** contains two independent molecules.

The crystallographic data and details of the structure refinements are summarized in Table S4. Selected bond distances and angles are listed in Table S5 for **1** and Table S6 for **3**.

In contrast to previously reported bipyrrolidine- and 1,2-ethylenediamine derived aminopyridine Mn triflates, in **1** and **3**, only one of the triflates is coordinated to Mn, while the other Mn cite is occupied by H_2O molecule (Figure S7). In both complexes, hydrogens of the Mn-coordinated water molecules form O-H···O hydrogen bonds with the outer-sphere triflates, forming associates (supermolecules) consisting of two Mn complexes and two triflate molecules (Figure S8). The hydrogen bond parameters are collected in Table S7.

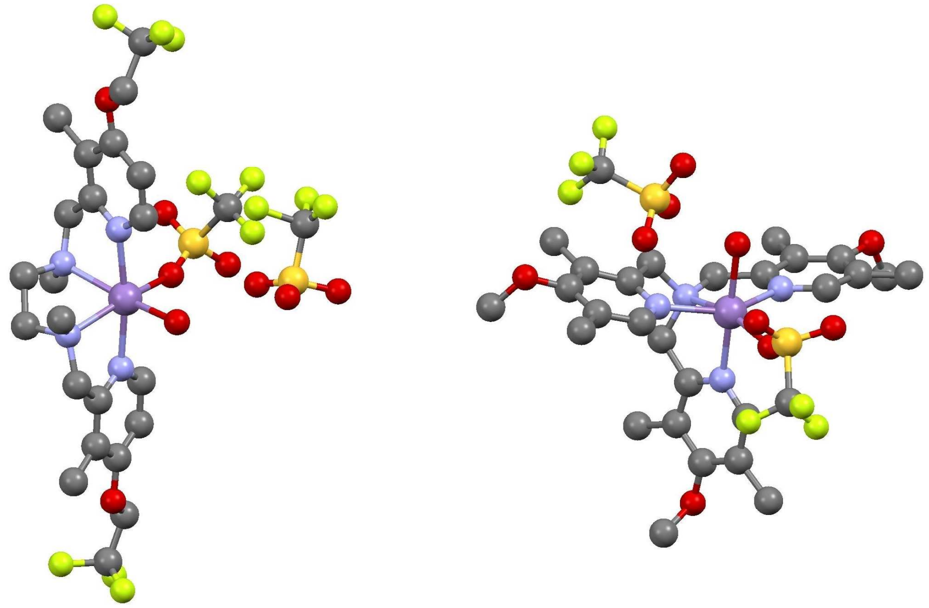

1 3

Figure S7. X-ray structures of complexes **1** and **3** in a ball-and-stick representation. Hydrogen atoms (and the second molecule for **3**) are omitted for clarity.

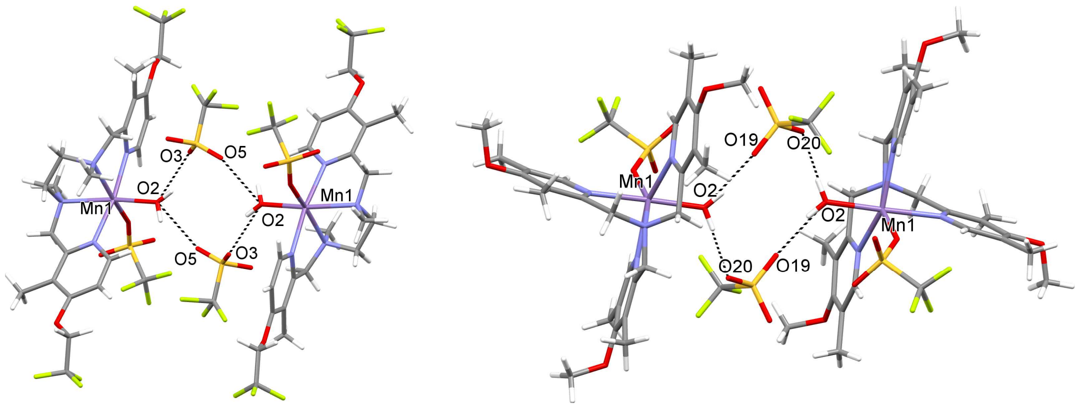

(A) (B)

Figure S8. The H-bonded supermolecules of **1** (A) and **3** (B). Each independent molecule of **3** forms the supermolecule independently (substantively).

Table S4. Crystal data and structure refinement for **1** and **3**.

Identification code	**1**	**3**
Empirical formula	$[C_{23}H_{30}F_9MnN_4O_6S]^{-}\cdot[CF_3SO_3]^{+}$	$[C_{28}H_{38}F_3MnN_4O_7S]^{-}\cdot[CF_3SO_3]^{+}$
Formula weight	865.58	835.69
Crystal system	Monoclinic	Triclinic
Space group	$P2_1/c$	*P-1*
a, Å	17.329(4)	15.800(2)
b, Å	14.618(3)	16.123(2)
c, Å	15.853(3)	16.406(2)
α	90.00	109.302(4)
β	104.790(6)	100.003(4)
γ	90.00	105.981(4)
V, Å^3	3883(2)	3626.8(6)
Z	4	4
D(calcd), g/cm^3	1.481	1.531
Absorption coefficient, mm^{-1}	0.553	0.568
F(000)	1756	1724
Crystal size, mm	1.00 × 0.80 × 0.30	0.77 × 0.12 × 0.06
θ range for data collection, deg.	1.22–25.25	1.37–31.11
Index ranges	$-20 \le h \le 20$, $-15 \le k \le 17$, $-18 \le l \le 18$	$-22 \le h \le 22$, $-23 \le k \le 23$, $-23 \le l \le 23$
Reflections collected / independent	36553 / 6966	85834 / 23058
R_{int}	0.1214	0.0747
$T_{min} - T_{max}$	0.4602 − 0.7452	0.7414 − 0.8018
Reflections with $I > 2\sigma(I)$	3504	12510
Data / restraints / parameters	6966 / 32 / 479	23058 / 6 / 967
Goodness-of-fit on F^2	1.024	1.001
Final R_1 [$I > 2\sigma(I)$]	0.1093	0.0557
wR_2 (all data)	0.3234	0.1714
Largest diff. peak / hole, e/Å^3	1.119 / −0.680	0.908 / −0.471

Table S5. Selected bond lengths and angles for **1**.

Bond	*d*, Å	Bond	*d*, Å
Mn(1) – O(1)	2.171(6)	Mn(1) – O(2)	2.167(6)
Mn(1) – N(1)	2.241(7)	Mn(1) – N(2)	2.273(6)
Mn(1) –N(3)	2.293(6)	Mn(1) – N(4)	2.223(6)
Angle	ω, deg.	Angle	ω, deg.
O(1)–Mn(1)–O(2)	97.9(2)	O(2)–Mn(1)–N(1)	90.7(2)
O(1)–Mn(1)–N(1)	91.7(2)	O(2)–Mn(1)–N(2)	92.6(2)
O(1)–Mn(1)–N(2)	162.7(2)	O(2)–Mn(1)–N(3)	163.6(2)
O(1)–Mn(1)–N(3)	93.1(2)	O(2)–Mn(1)–N(4)	94.1(2)
O(1)–Mn(1)–N(4)	94.2(2)	N(1)–Mn(1)–N(2)	74.3(2)
N(2)–Mn(1)–N(3)	80.0(2)	N(1)–Mn(1)–N(3)	101.2(2)
N(2)–Mn(1)–N(4)	98.9(2)	N(1)–Mn(1)–N(4)	171.8(2)
N(3)–Mn(1)–N(4)	72.8(2)		

Table S6. Selected bond lengths and angles for two independent molecules of **3**.

Bond	*d*, Å	Bond	*d*, Å
Mn(1) – O(1)	2.130(3)	Mn(2) - O(3)	2.116(3)
Mn(1) – O(2)	2.152(2)	Mn(2) - O(4)	2.184(2)
Mn(1) – N(1)	2.216(2)	Mn(2) - N(5)	2.199(2)
Mn(1) – N(2)	2.305(2)	Mn(2) - N(6)	2.301(2)
Mn(1) – N(3)	2.263(2)	Mn(2) - N(7)	2.239(2)
Mn(1) – N(4)	2.252(2)	Mn(2) - N(8)	2.259(2)
Angle	ω, deg.	Angle	ω, deg.
O(1)–Mn(1)–O(2)	94.43(10)	O(3)–Mn(2)–O(4)	95.11(9)
O(1)–Mn(1)–N(1)	105.54(10)	O(3)–Mn(2)–N(5)	103.44(10)
O(1)–Mn(1)–N(2)	160.97(8)	O(3)–Mn(2)–N(6)	161.91(8)
O(1)–Mn(1)–N(3)	106.92(10)	O(3)–Mn(2)–N(7)	109.65(10)
O(1)–Mn(1)–N(4)	86.19(9)	O(3)–Mn(2)–N(8)	87.68(9)
O(2)–Mn(1)–N(1)	89.49(8)	O(4)–Mn(2)–N(5)	86.57(8)
O(2)–Mn(1)–N(2)	104.53(9)	O(4)–Mn(2)–N(6)	102.96(8)
O(2)–Mn(1)–N(3)	84.59(9)	O(4)–Mn(2)–N(7)	83.69(8)
O(2)–Mn(1)–N(4)	178.79(9)	O(4)–Mn(2)–N(8)	176.62(9)
N(1)–Mn(1)–N(2)	76.78(8)	N(5)–Mn(2)–N(6)	77.03(8)
N(1)–Mn(1)–N(3)	147.35(9)	N(5)–Mn(2)–N(7)	146.16(9)
N(1)–Mn(1)–N(4)	89.35(8)	N(5)–Mn(2)–N(8)	90.93(8)
N(2)–Mn(1)–N(3)	73.76(8)	N(6)–Mn(2)–N(7)	73.77(8)
N(2)–Mn(1)–N(4)	74.89(8)	N(6)–Mn(2)–N(8)	74.23(8)
N(3)–Mn(1)–N(4)	96.23(8)	N(7)–Mn(2)–N(8)	97.18(8)

Table S7. Parameters of H-bonds of **1** and **3**

complex	interaction	O-H, Å	H O, Å	O O, Å	O–H O, °
**2**	O2–H1W O3	0.82(6)	2.00(6)	2.766(9)	155(6)
	O2–H2W O5	0.82(5)	2.01(8)	2.650(14)	135(7)
**4**	O2–H1W O19	0.82(3)	1.95(3)	2.765(3)	168(4)
	O2–H2W O20	0.82(2)	1.95(2)	2.768(3)	174(4)
	O4–H3W O16	0.84(3)	1.95(3)	2.785(3)	172(3)
	O4–H4W O15	0.83(2)	1.94(2)	2.766(3)	176(4)

DFT calculations: computational methods.

Calculations on cationic parts of complexes [Λ-Cat((*R*)-1-phenylethanol)(CH_3CN)] and [Λ-Cat((*S*)-1-phenylethanol)(CH_3CN)] (**[Λ-Cat·R]** and **[Λ-Cat·S]**) were performed with the hybrid B3-LYP[S5,S6] density functional theory scheme (DFT) using GAUSSIAN 09 program suite, with the LANL2DZ basis set with associated Hay–Wadt nonrelativistic effective core potential for the Mn atom[S7] and 6-311G(d)[S8] basis set for other atoms. Calculations on **^{5}[Λ-Cat·R] in CH_3CN** and **^{5}[Λ-Cat·S] in CH_3CN** were also done with UB3LYP and PBE0[S9] functionals. Geometry optimizations were carried out without symmetry restrictions. Solvation effects (with CH_3CN or H_2O) were incorporated using polarized continuum model (PCM) method as implemented in GAUSSIAN 09. The results are presented in Table S8 and Figure S9.

Table S8. Selected solvation-corrected energies, bond lengths and spin densities for Mn complexes.

Entry	Complex	*E*, a.u.	Δ*E*, kcal/mol	Bond lengths, Å		Spin density			
				**Mn–O**	**Mn–N**(≡CCH_3)	**Mn**	**O(R*)**	**N**(≡CCH_3)	**N_4 (Ligand)**
**1**	**^{5}[Λ-Cat·R] in CH_3CN**	-1850.64742	2.330	1.81	2.34	3.95	0.06	0.01	-0.04
**2**	**^{5}[Λ-Cat·S] in CH_3CN**	-1850.64794	2.003	1.81	2.34	3.96	0.05	0.01	-0.04
**3**	**^{3}[Λ-Cat·R] in CH_3CN**	-1850.63793	8.284	1.81	2.03	1.98	0.15	-0.02	-0.12
**4**	**^{3}[Λ-Cat·S] in CH_3CN**	-1850.63834	8.021	1.81	2.03	1.99	0.15	-0.02	-0.12
**5**	**^{1}[Λ-Cat·R] in CH_3CN**	-1850.59556	34.853	1.76	2.02	-	-	-	-
**6**	**^{1}[Λ-Cat·S] in CH_3CN**	-1850.59633	34.368	1.76	2.02	-	-	-	-
7	**^{5}[Λ-Cat·R] in H_2O**	-1850.65065	0.307	1.81	2.34	3.95	0.06	0.01	-0.04
**8**	**^{5}[Λ-Cat·S] in H_2O**	-1850.65114	0	1.81	2.34	3.96	0.05	0.01	-0.04
**9**	**^{5}[Λ-Cat·R] in CH_3CN***	-1850.64742	0.325	1.81	2.34	3.95	0.06	0.01	-0.04
**10**	**^{5}[Λ-Cat·S] in CH_3CN***	-1850.64794	0*	1.81	2.34	3.96	0.05	0.01	-0.04
**11**	**^{5}[Λ-Cat·R] in CH_3CN****	-1848.53429	0.881	1.81	2.31	4.07	0.02	0.00	-0.08
**12**	**^{5}[Λ-Cat·S] in CH_3CN****	-1848.53569	0**	1.81	2.31	4.08	0.00	0.00	-0.08

* Calculated at the UB3LYP level. ** Calculated at the PBE0 level.

The calculations provide qualitative evidence that the [Λ-Cat·S] structure is energetically preferable with respect to [Λ-Cat·R], either in water or in acetonitrile solutions (cf. Table S8, entry 7 vs. 8, 9 vs. 10, and 11 vs. 12). On the PBE0 calculations level, the difference between [Λ-Cat·S] and [Λ-Cat·R] is 0.881 kcal/mol, which at -10 °C should corresponds to a [Λ-Cat·S]/[Λ-Cat·R] equilibrium ratio of 5.4; as 1st approximation, the existence of the less stable coordination stereoisomer, [Λ-Cat·R], could be neglected.

Similarly, [Δ-Cat·R] should prevail over [Δ-Cat·S]. In both cases, more preferable was the quintet state (overall $S = 2$), which is archetypical of d^4 Mn(III) ions.[S10] In the ^{5}[Λ-Cat·R] and ^{5}[Λ-Cat·S] structures, long Mn–N(≡CCH_3) and the opposite Mn–N(ligand) contacts (2.32 Å) are indicative of Jahn-Teller tetragonal elongation of the coordination octahedron, typical for high-spin d^4 metal complexes.[S11]

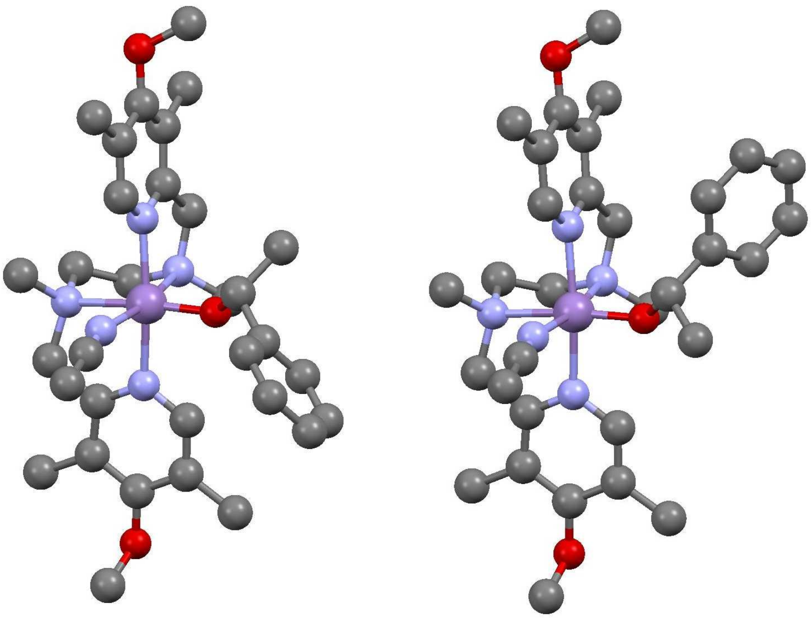

^{5}[Λ-Cat·R] in CH_3CN **^{5}[Λ-Cat·R] in CH_3CN**

Figure S9. DFT optimized structures of [Λ-Cat((*R*)-1-phenylethanol)(CH_3CN)] and [Λ-Cat((*S*)-1-phenylethanol)(CH_3CN)] in CH_3CN at the B3-LYP level. Hydrogen atoms are omitted for clarity.

Additional references

S1. G. M. Sheldrick, *Acta Crystallogr. Sect. A*, 2008, **64**, 112–122.

S2. SADABS, v. 2008-1, Bruker AXS, Madison, WI, USA, 2008.

S3. A. L. Spek, PLATON, A Multipurpose Crystallographic Tool, version 10M, Utrecht University, The Netherlands, 2003.

S4. A. L. Spek, *J. Appl. Crystallogr.*, 2003, **36**, 7–13.

S5. A. D. Becke, *J. Chem. Phys.* 1993, **98**, 5648-5652.

S6. C. Lee, W. Yang, R. G. Parr, *Phys. Rev. B*, 1988, **37**, 785-789.

S7. W. R. Wadt, P. J. Hay, *J. Chem. Phys.*, 1985, **82**, 284-98.

S8. E. R. Davidson, D. Feller, *Chem. Rev.*, 1986, **86**, 681-696.

S9. C. Adamo, V. Barone, *J. Chem. Phys.*, 1999, **110**, 6158-69.

S10. D. P. Goldberg, J. Tesler, J. Krzystek, A. G. Montalban, L. C. Brunel, A. G. M. Barrett, B. M. Hoffman, *J. Am. Chem. Soc.*, 1997, **119**, 8722-8723.

S11. D. F. Shriver, P. W. Atkins, Inorganic Chemistry (3rd ed.). Oxford University Press, 1999.

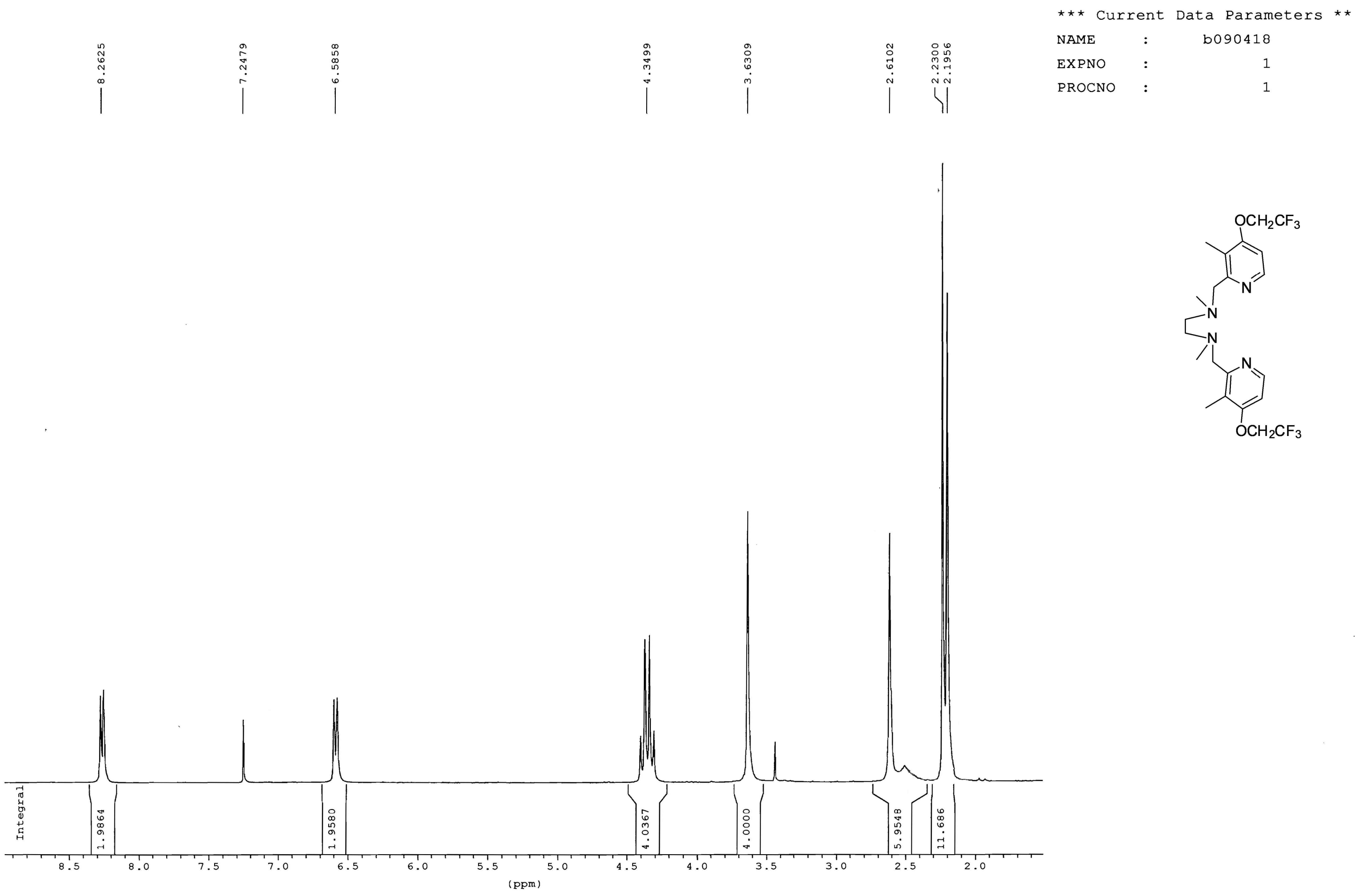

^{1}H NMR spectrum ($CDCl_3/CCl_4$, 25 °C) of *N*[1],*N*[2]-dimethyl-*N*1,*N*2-bis((3-methyl-4-(2,2,2-trifluoroethoxy)pyridin-2-yl)methyl)ethane-1,2-diamine.

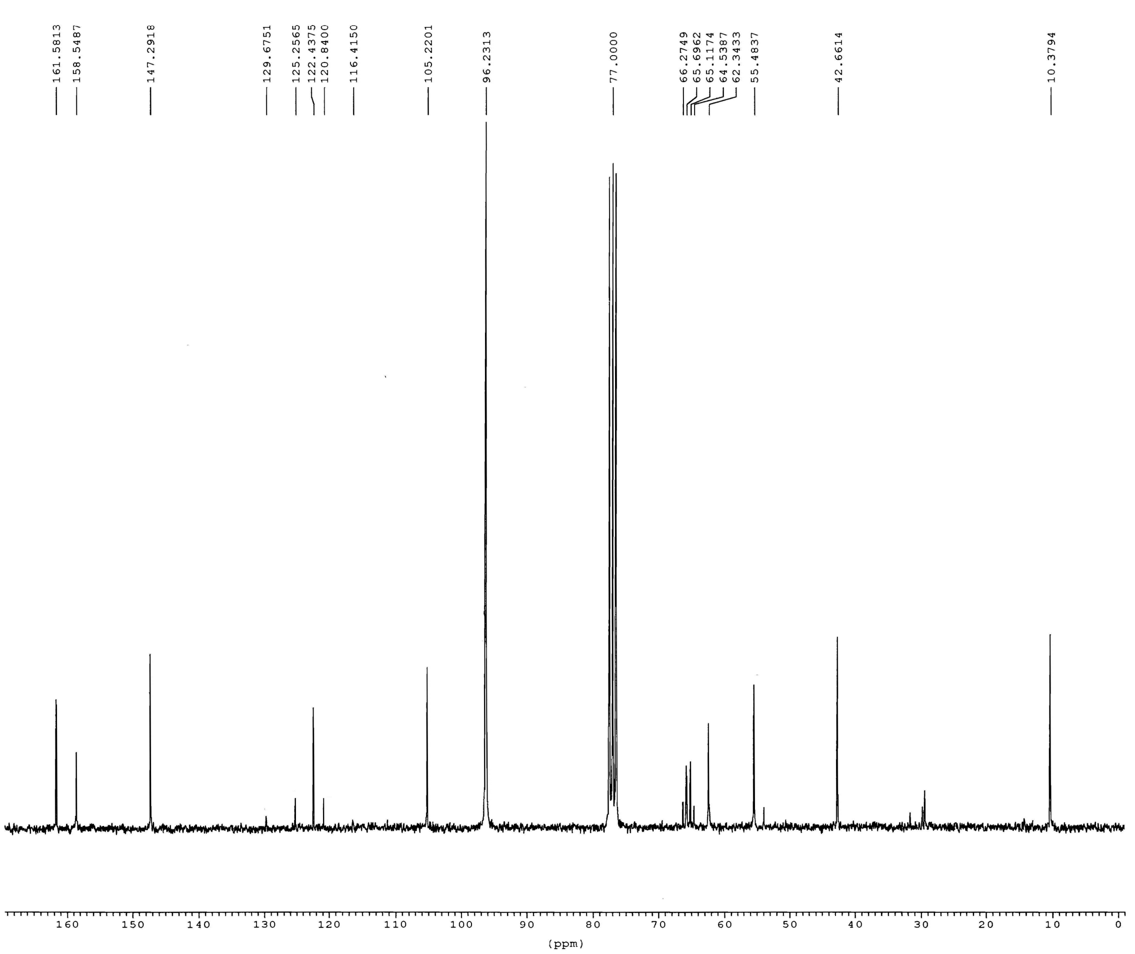

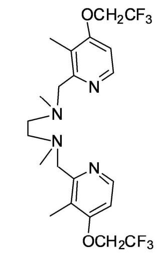

$^{13}C\{^1H\}$ NMR spectrum ($CDCl_3/CCl_4$, 25 °C) of *N*[1],*N*[2]-dimethyl-*N*1,*N*2-bis((3-methyl-4-(2,2,2-trifluoroethoxy)pyridin-2-yl)methyl)ethane-1,2-diamine.

HPLC traces

Table 1, Entry 2 / Figure 2A, green trace

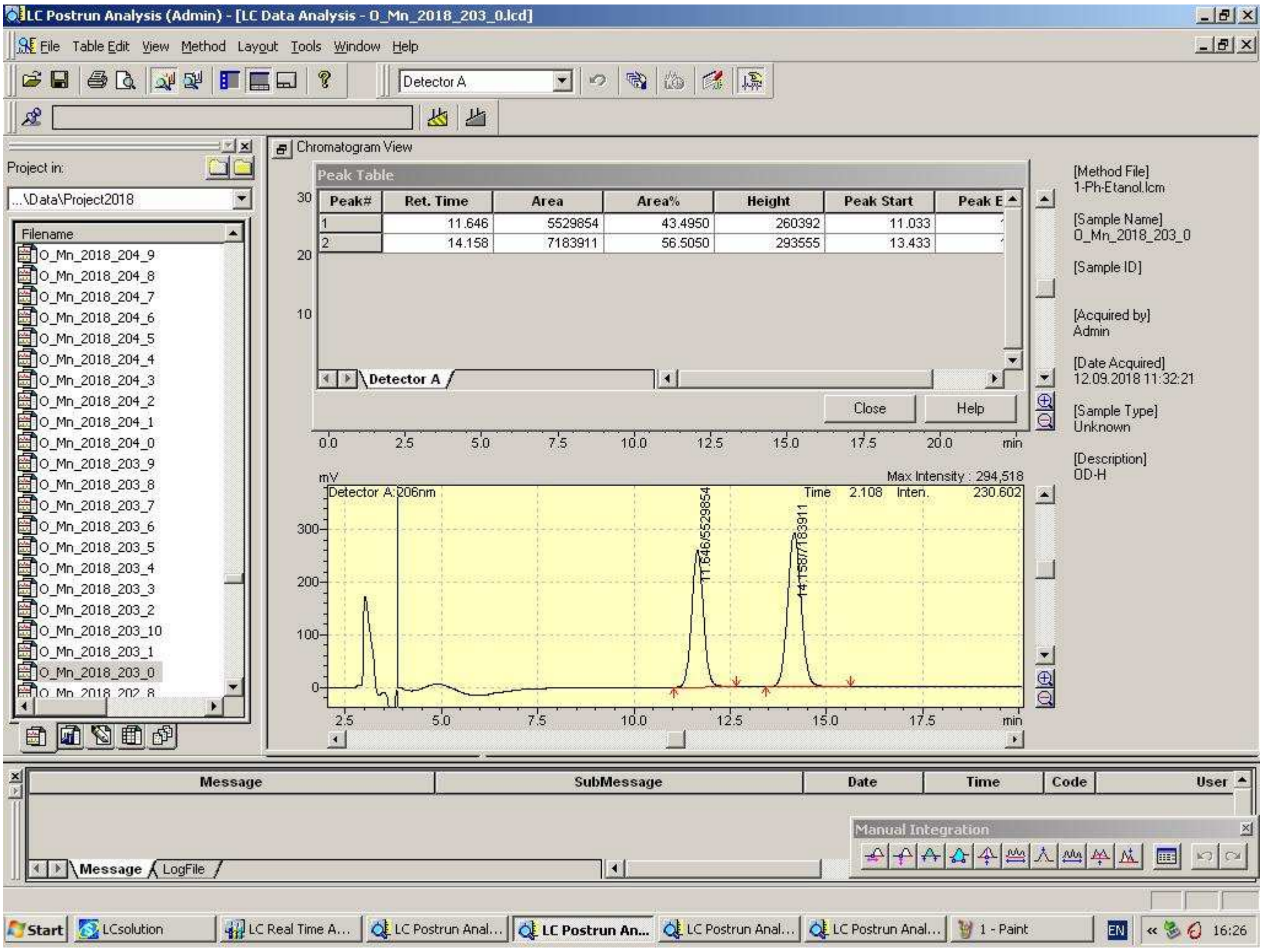

Table 1, Entry 2 / Figure 2A, green trace, 0th point			
Time, min	Area, %	Assignment	Amount(%)
11.646	43.4950	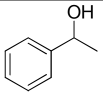 (*R*)-	100 %, 13.0 % *ee*
14.158	56.5050	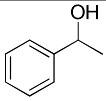 (*S*)-	

Conditions: Chiralcel OD-H, *i*-PrOH/ hexane 3:97, 1.0 mL/min, λ 206 nm, 20 °C.

Notice: Artifacts, originating from the eluent "front" are observed at 3-5 min: they were not integrated.

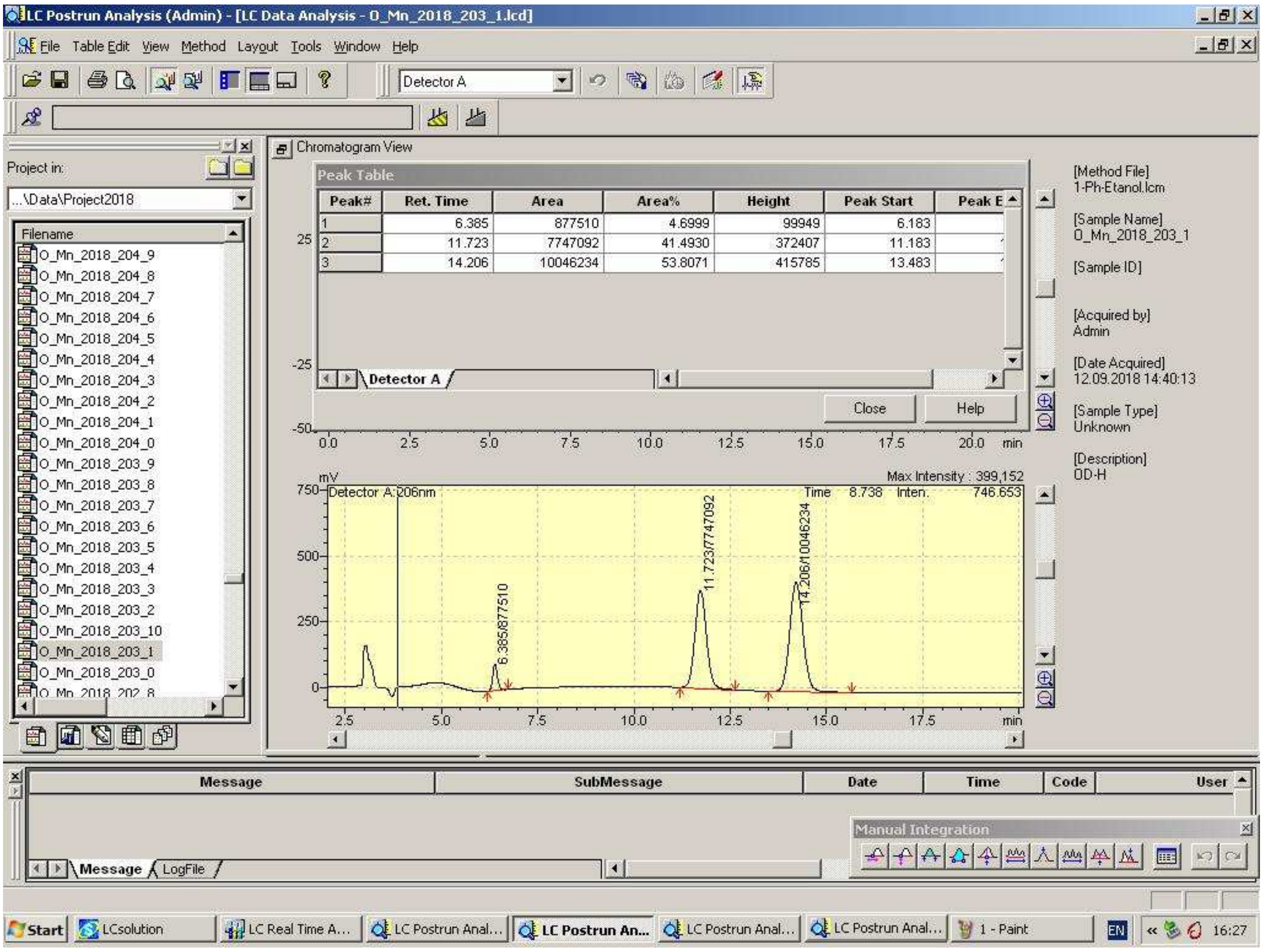

Table 1, Entry 2 / Figure 2A, green trace, 1st point			
Time, min	Area, %	Assignment	Amount(%)
6.385	4.6999	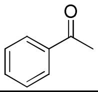	5.3 %
11.723	41.4930	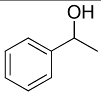 (*R*)-	94.7 %, 12.9 % *ee*
14.206	53.8071	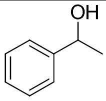 (*S*)-	

Conditions: Chiralcel OD-H, *i*-PrOH/ hexane 3:97, 1.0 mL/min, *λ* 206 nm, 20 °C.

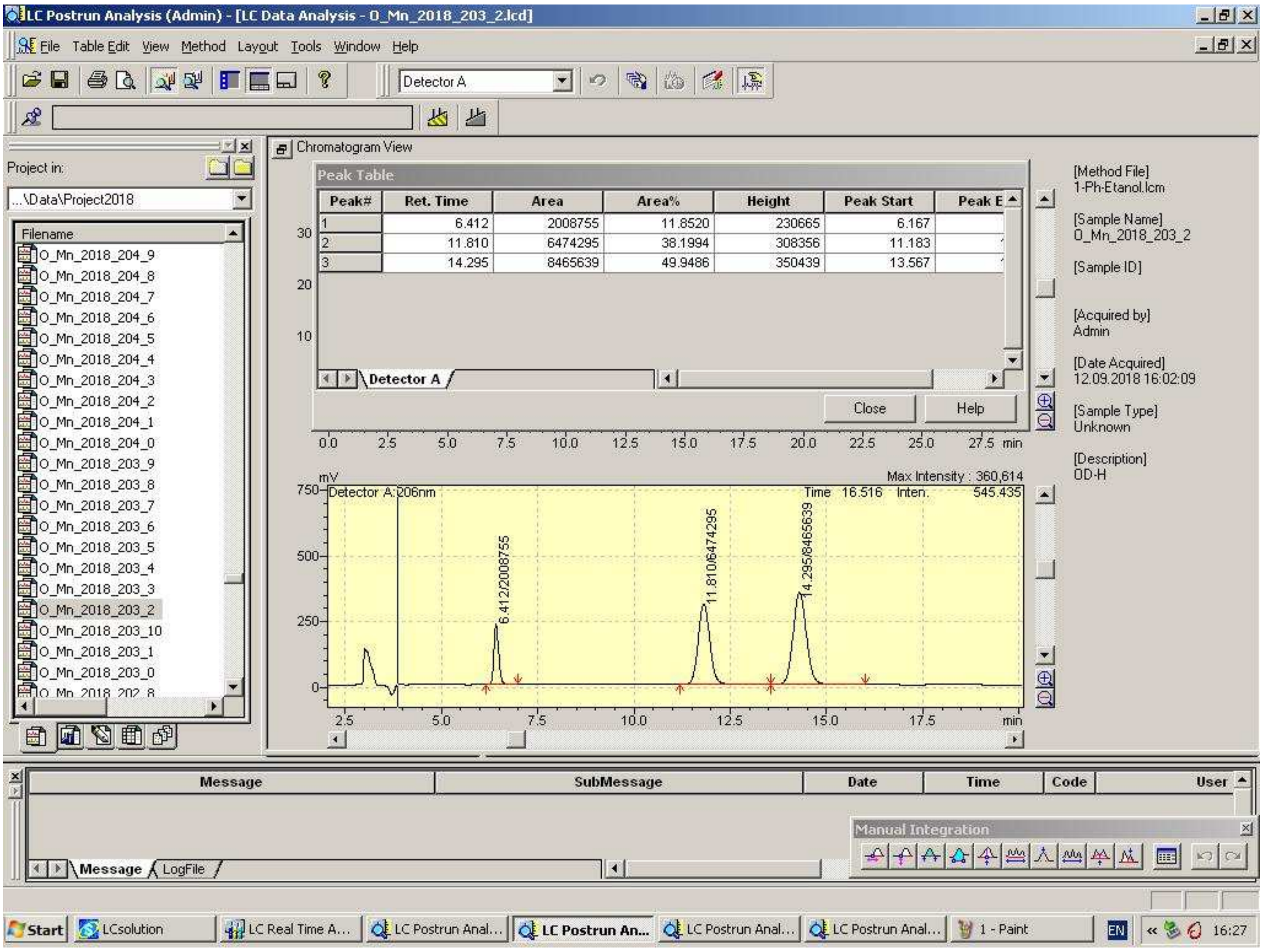

Table 1, Entry 2 / Figure 2A, green trace, 2nd point			
Time, min	Area, %	Assignment	Amount(%)
6.412	11.8520	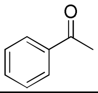	13.4 %
11.810	38.1994	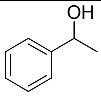 (*R*)-	86.4 %, 13.3 % *ee*
14.295	49.9486	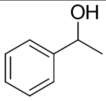 (*S*)-	

Conditions: Chiralcel OD-H, *i*-PrOH/ hexane 3:97, 1.0 mL/min, λ 206 nm, 20 °C.

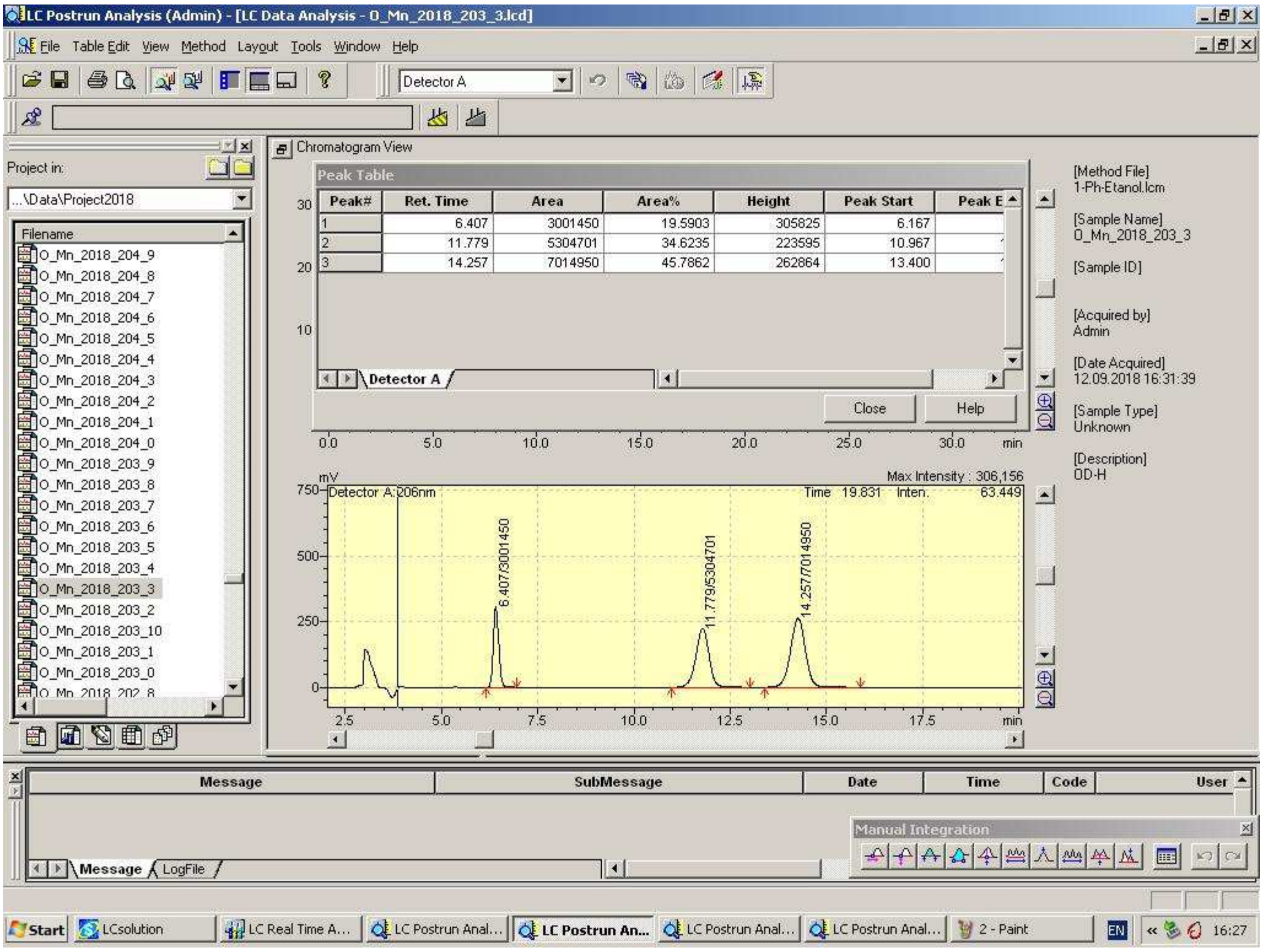

Table 1, Entry 2 / Figure 2A, green trace, 3rd point			
Time, min	Area, %	Assignment	Amount(%)
6.407	19.5903	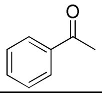	21.9 %
11.779	34.6235	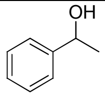 (*R*)-	78.1 %, 13.9 % *ee*
14.257	45.7862	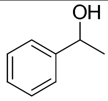 (*S*)-	

Conditions: Chiralcel OD-H, *i*-PrOH/ hexane 3:97, 1.0 mL/min, *λ* 206 nm, 20 °C.

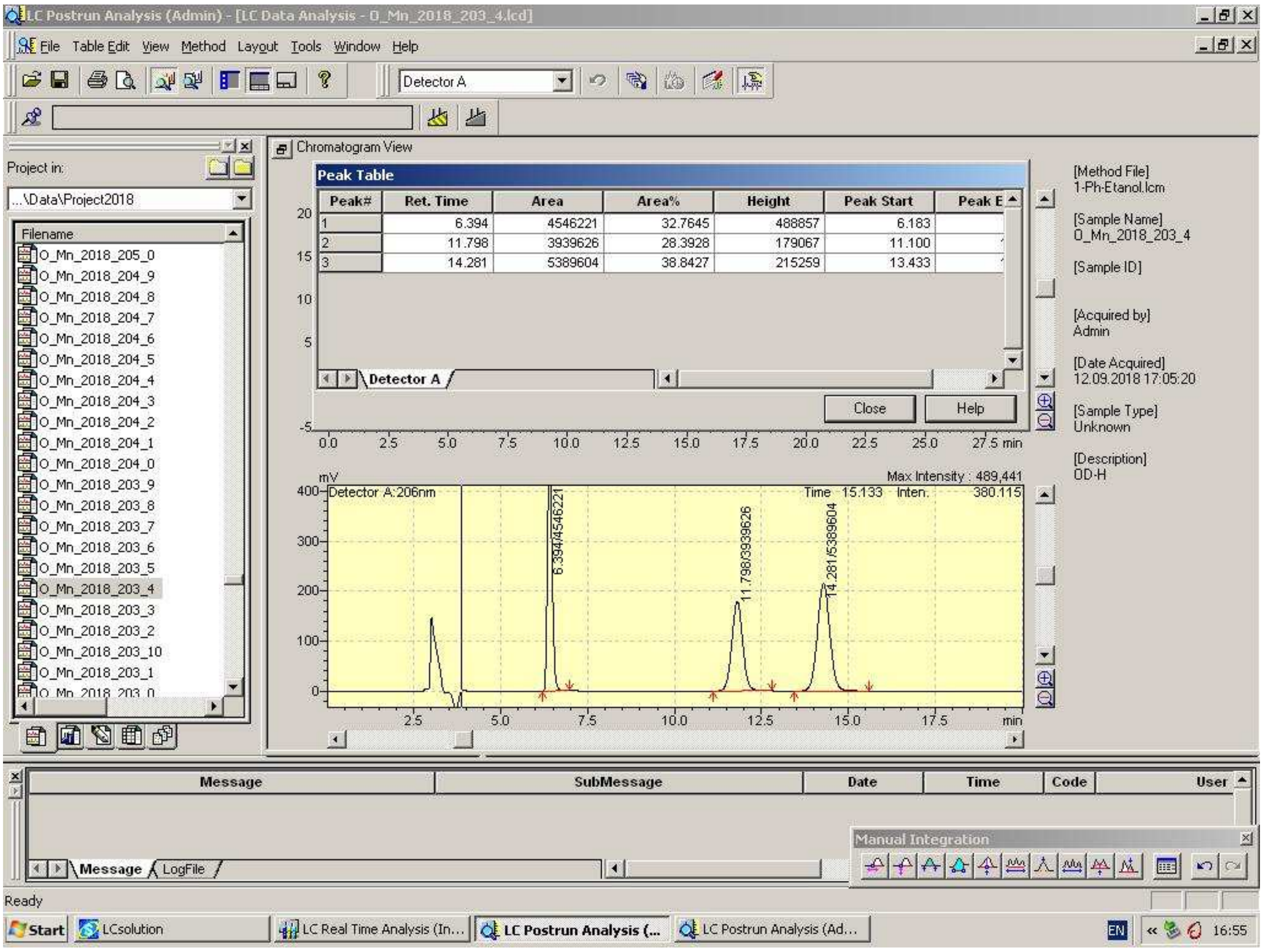

<table>
<tr><th colspan="4">Table 1, Entry 2 / Figure 2A, green trace, 4th point</th></tr>
<tr><td>Time, min</td><td>Area, %</td><td>Assignment</td><td>Amount(%)</td></tr>
<tr><td>6.394</td><td>32.7645</td><td>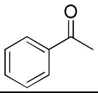</td><td>35.9 %</td></tr>
<tr><td>11.798</td><td>28.3928</td><td>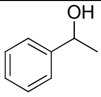 (R)-</td><td rowspan="2">64.1 %, 15.5 % ee</td></tr>
<tr><td>14.281</td><td>38.8427</td><td>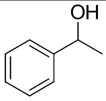 (S)-</td></tr>
</table>

Conditions: Chiralcel OD-H, *i*-PrOH/ hexane 3:97, 1.0 mL/min, λ 206 nm, 20 °C.

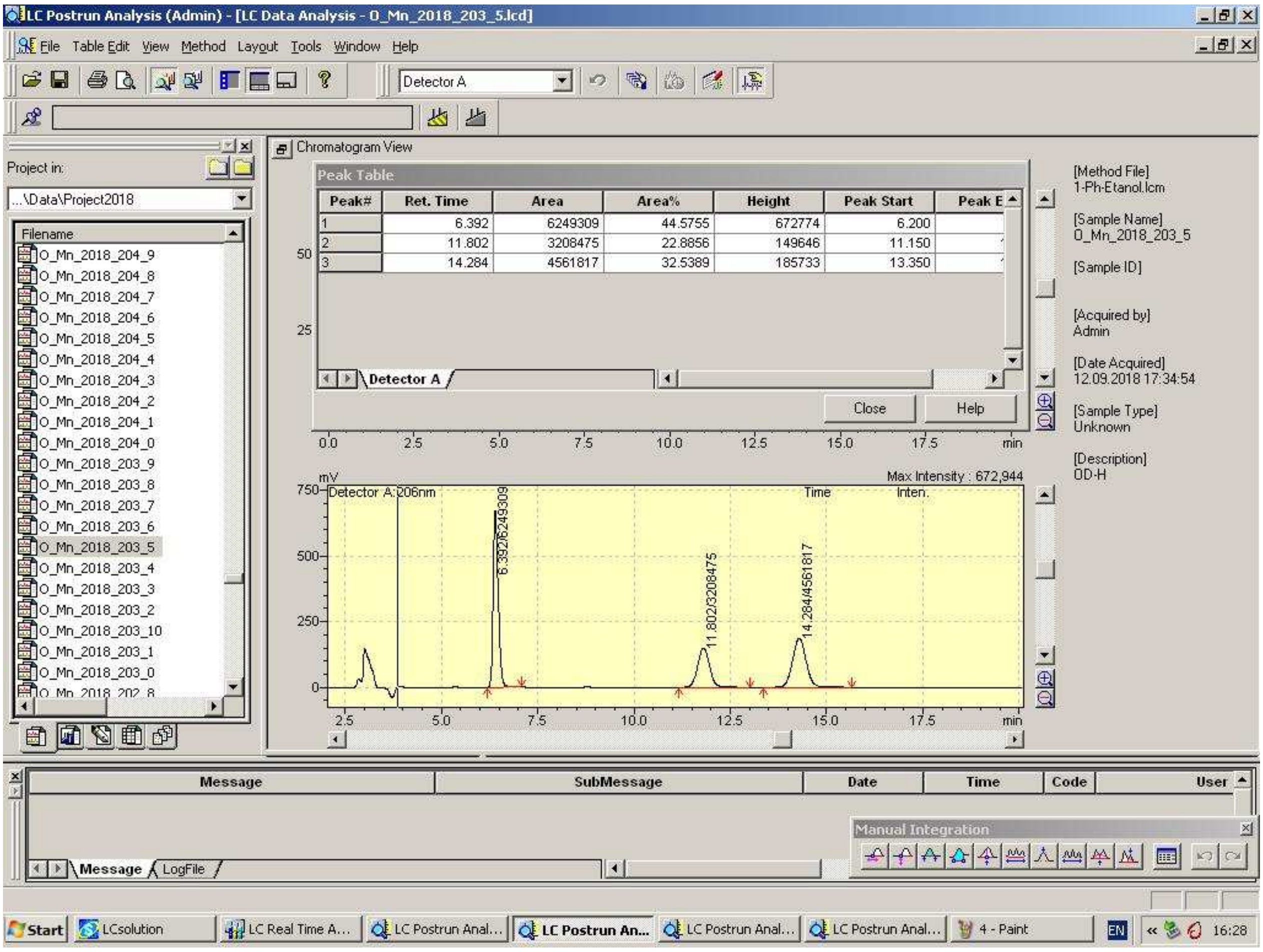

Table 1, Entry 2 / Figure 2A, green trace, 5th point			
Time, min	Area, %	Assignment	Amount(%)
6.392	44.5755	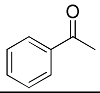	48.0 %
11.802	22.8856	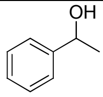 (*R*)-	52.0 %, 17.4 % *ee*
14.284	32.5389	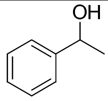 (*S*)-	

Conditions: Chiralcel OD-H, *i*-PrOH/ hexane 3:97, 1.0 mL/min, *λ* 206 nm, 20 °C.

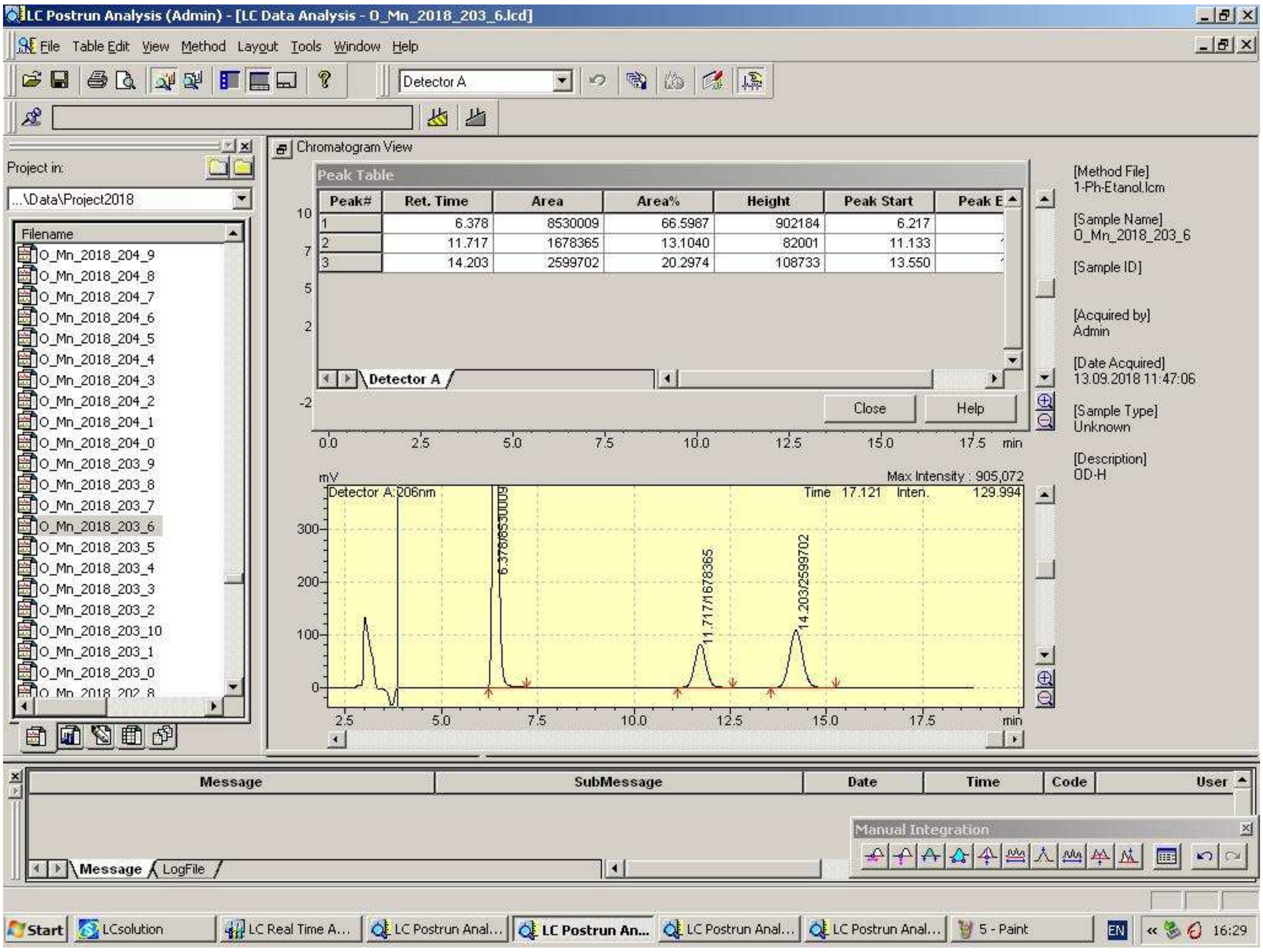

Table 1, Entry 2 / Figure 2A, green trace, 6th point			
Time, min	Area, %	Assignment	Amount(%)
6.378	66.5987	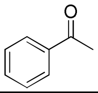	69.6 %
11.717	13.1040	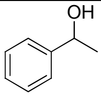 (*R*)-	30.4 %, 21.5 % *ee*
14.203	20.2974	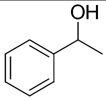 (*S*)-	

Conditions: Chiralcel OD-H, *i*-PrOH/ hexane 3:97, 1.0 mL/min, λ 206 nm, 20 °C.

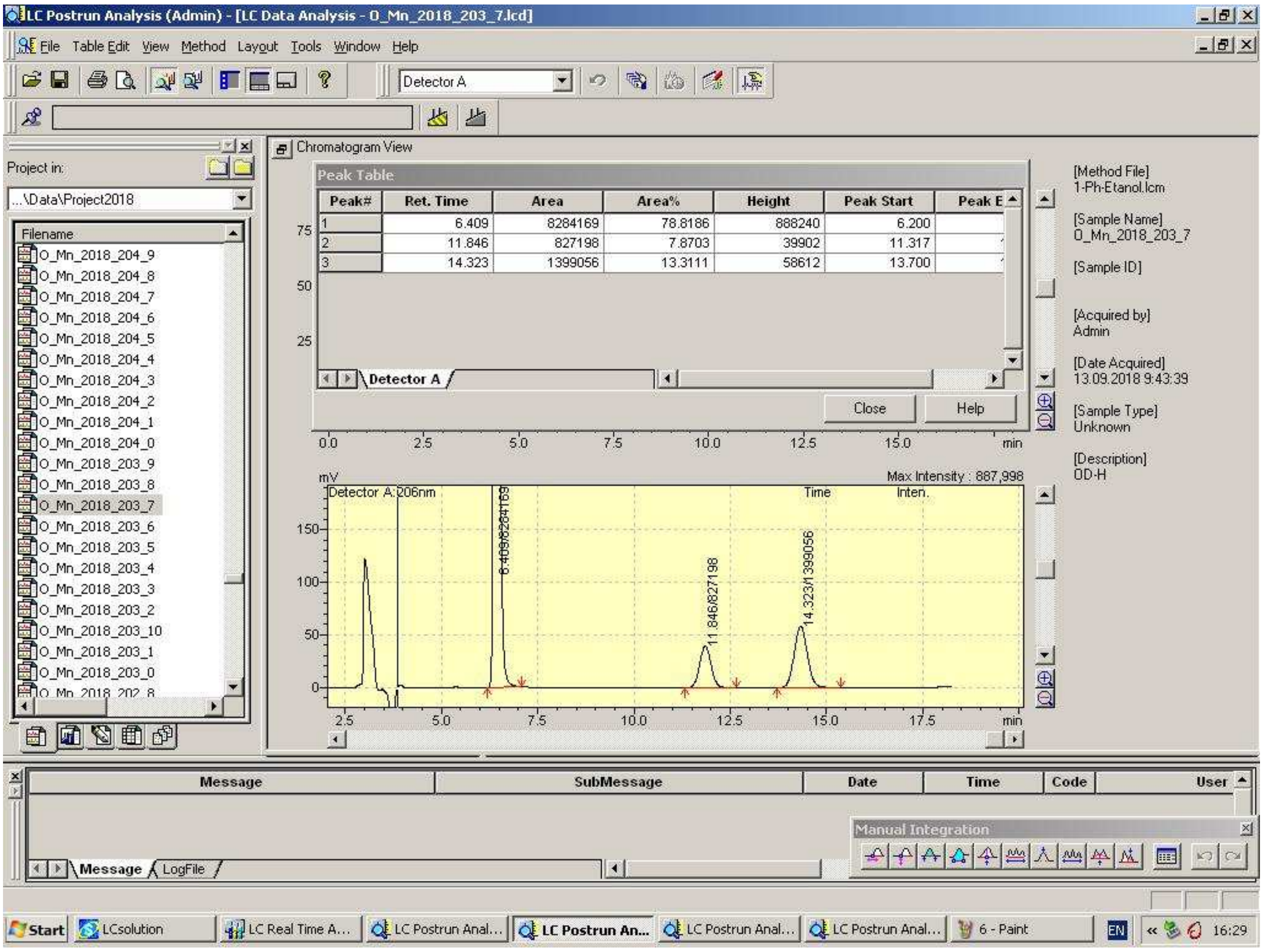

Table 1, Entry 2 / Figure 2A, green trace, 7th point			
Time, min	Area, %	Assignment	Amount(%)
6.409	78.8186	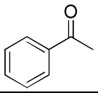	80.1 %
11.846	7.8703	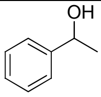 (*R*)-	19.9 %, 26.3 % *ee*
14.323	13.3111	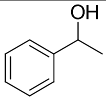 (*S*)-	

Conditions: Chiralcel OD-H, *i*-PrOH/ hexane 3:97, 1.0 mL/min, λ 206 nm, 20 °C.

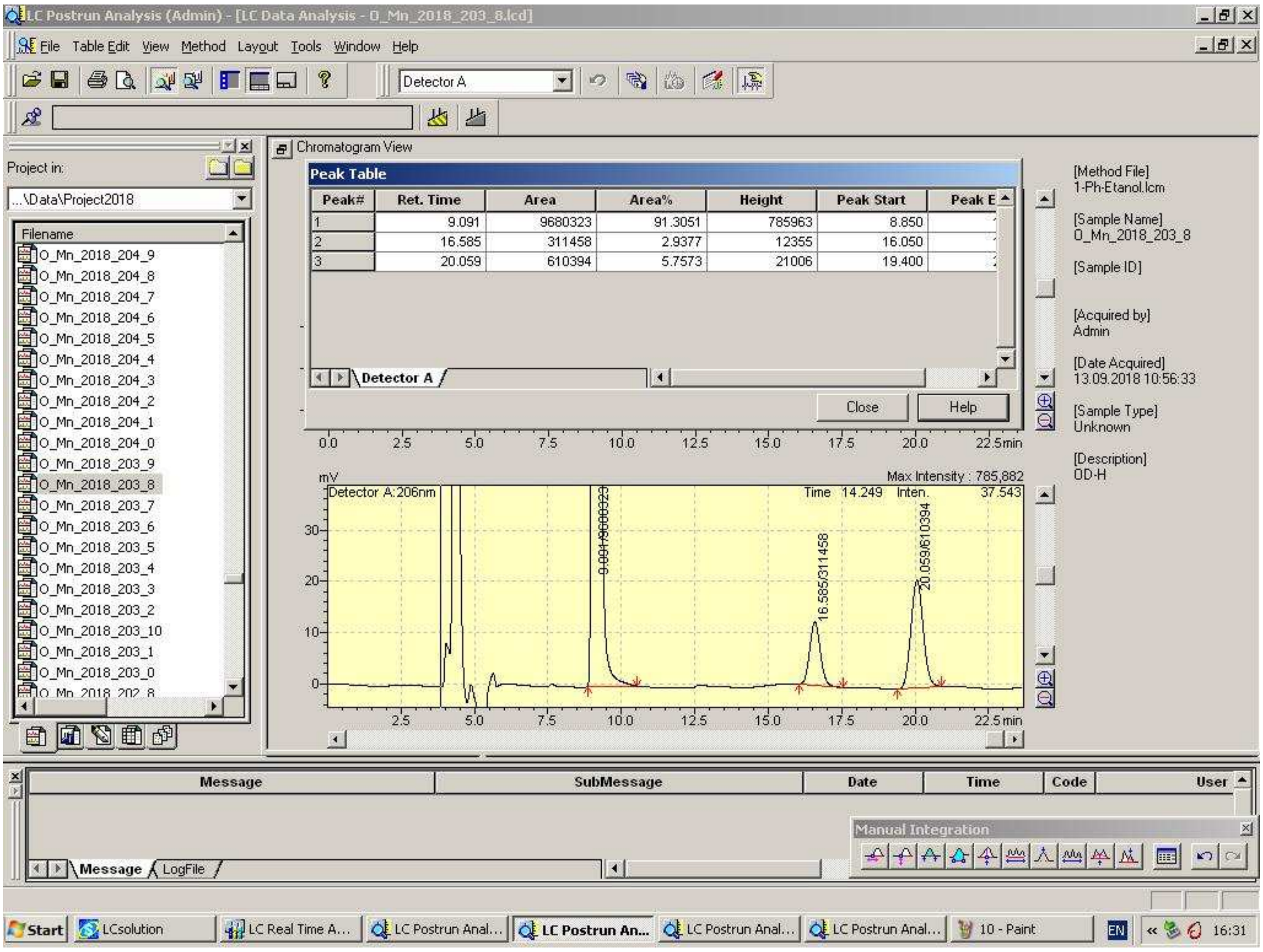

**Table 1, Entry 2 / Figure 2A, green trace, 8th point**			
Time, min	Area, %	Assignment	Amount(%)
9.091	91.3051	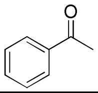	92.3 %
16.585	2.9377	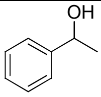 (*R*)-	7.7 %, 32.4 % *ee*
20.059	5.7573	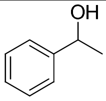 (*S*)-	

Conditions: Chiralcel OD-H, *i*-PrOH/ hexane 3:97, 0.7 mL/min, *λ* 206 nm, 20 °C.

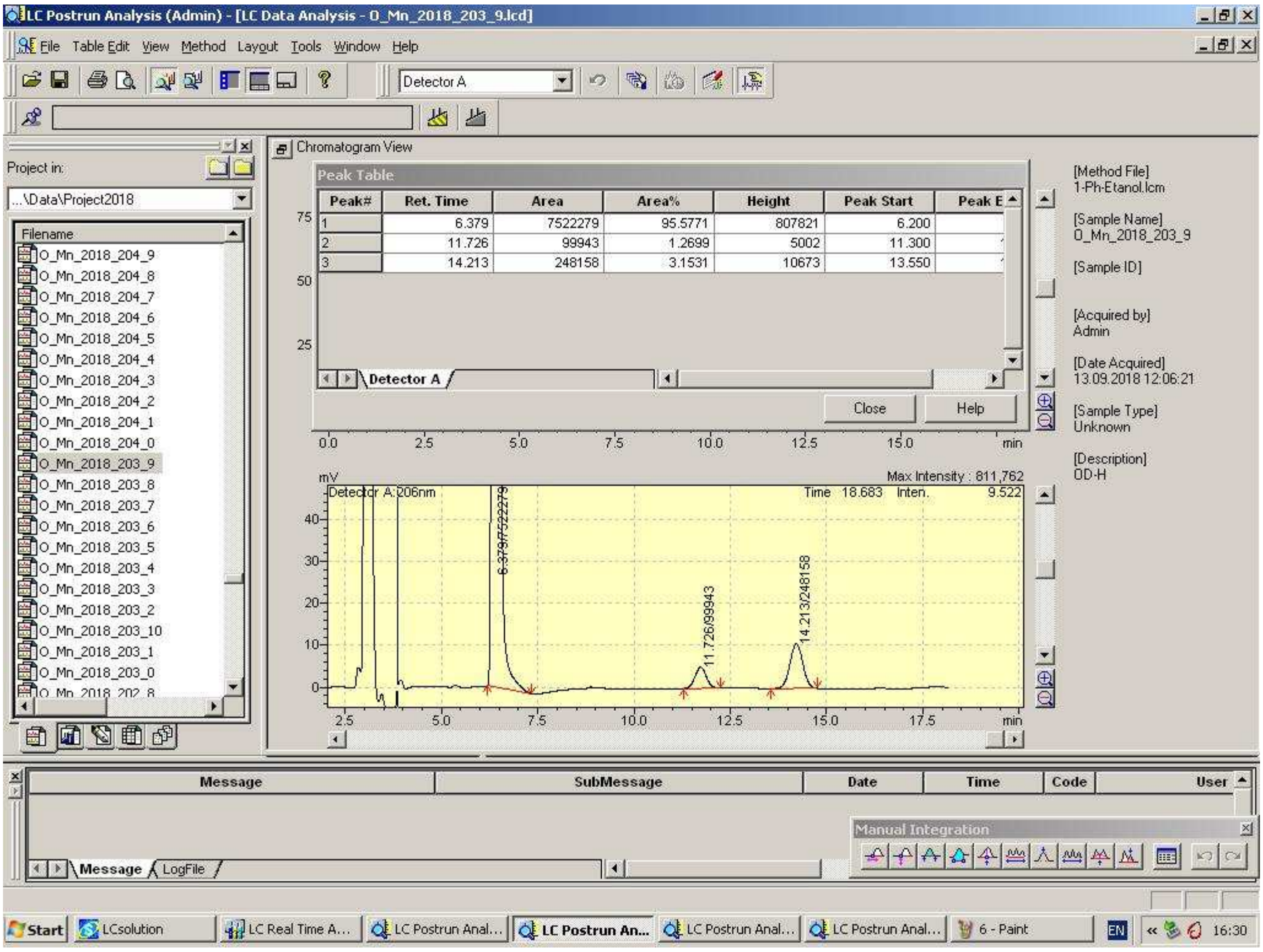

Table 1, Entry 2 / Figure 2A, green trace, 9th point			
Time, min	Area, %	Assignment	Amount(%)
6.379	95.5771	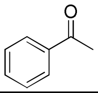	96.1 %
11.726	1.2699	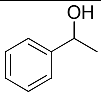 (*R*)-	3.9 %, 42.6 % *ee*
14.213	3.1531	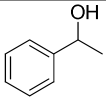 (*S*)-	

Conditions: Chiralcel OD-H, *i*-PrOH/ hexane 3:97, 1.0 mL/min, *λ* 206 nm, 20 °C.

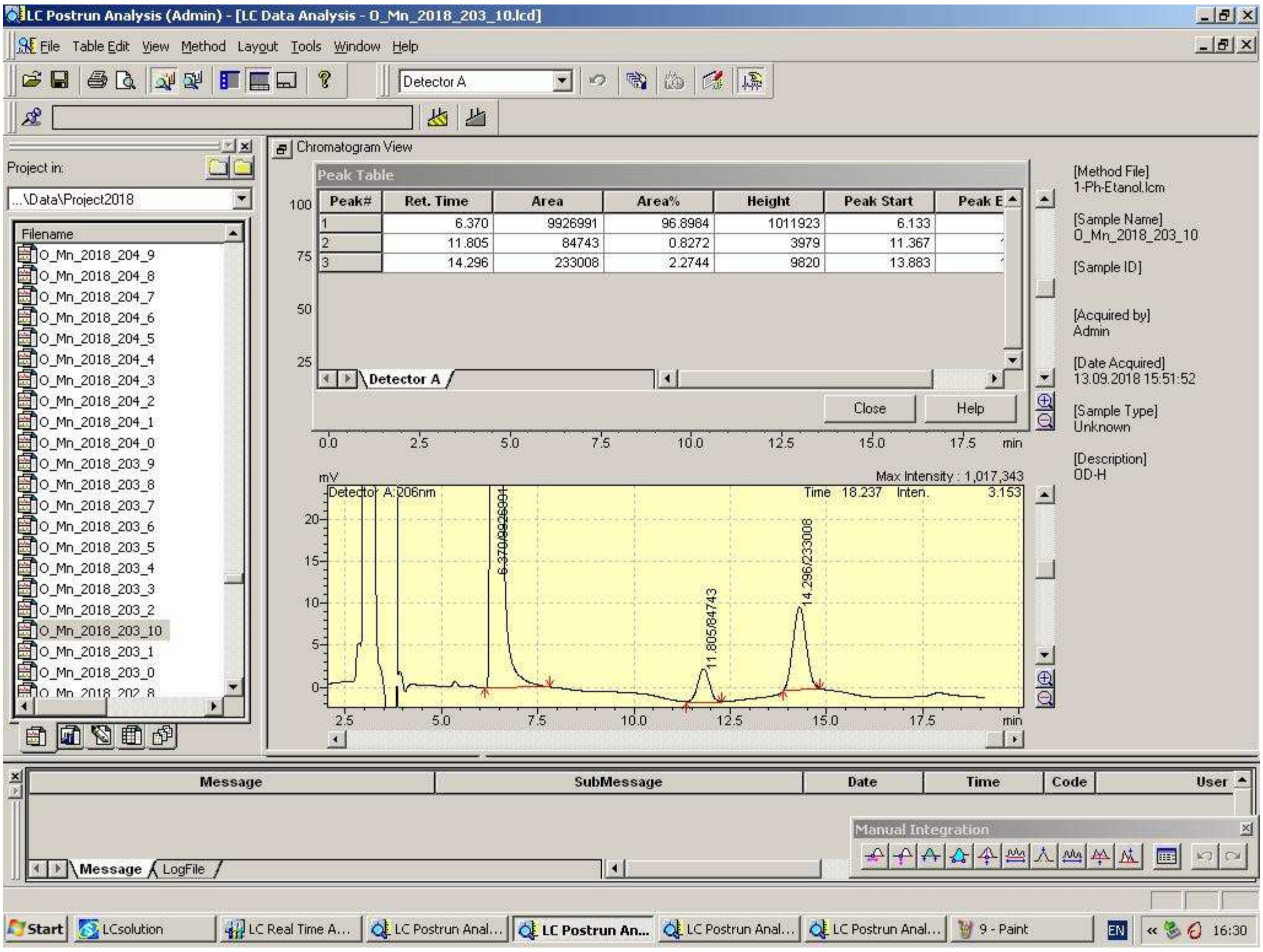

Table 1, Entry 2 / Figure 2A, green trace, 10th point			
Time, min	Area, %	Assignment	Amount(%)
6.370	96.8984	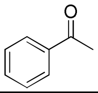	97.3 %
11.805	0.8272	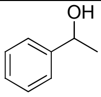 (*R*)-	2.7 %, 46.7 % *ee*
14.296	2.2744	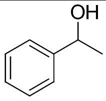 (*S*)-	

Conditions: Chiralcel OD-H, *i*-PrOH/ hexane 3:97, 1.0 mL/min, *λ* 206 nm, 20 °C.

Table 1, Entry 8 / Figure 3A, violet trace

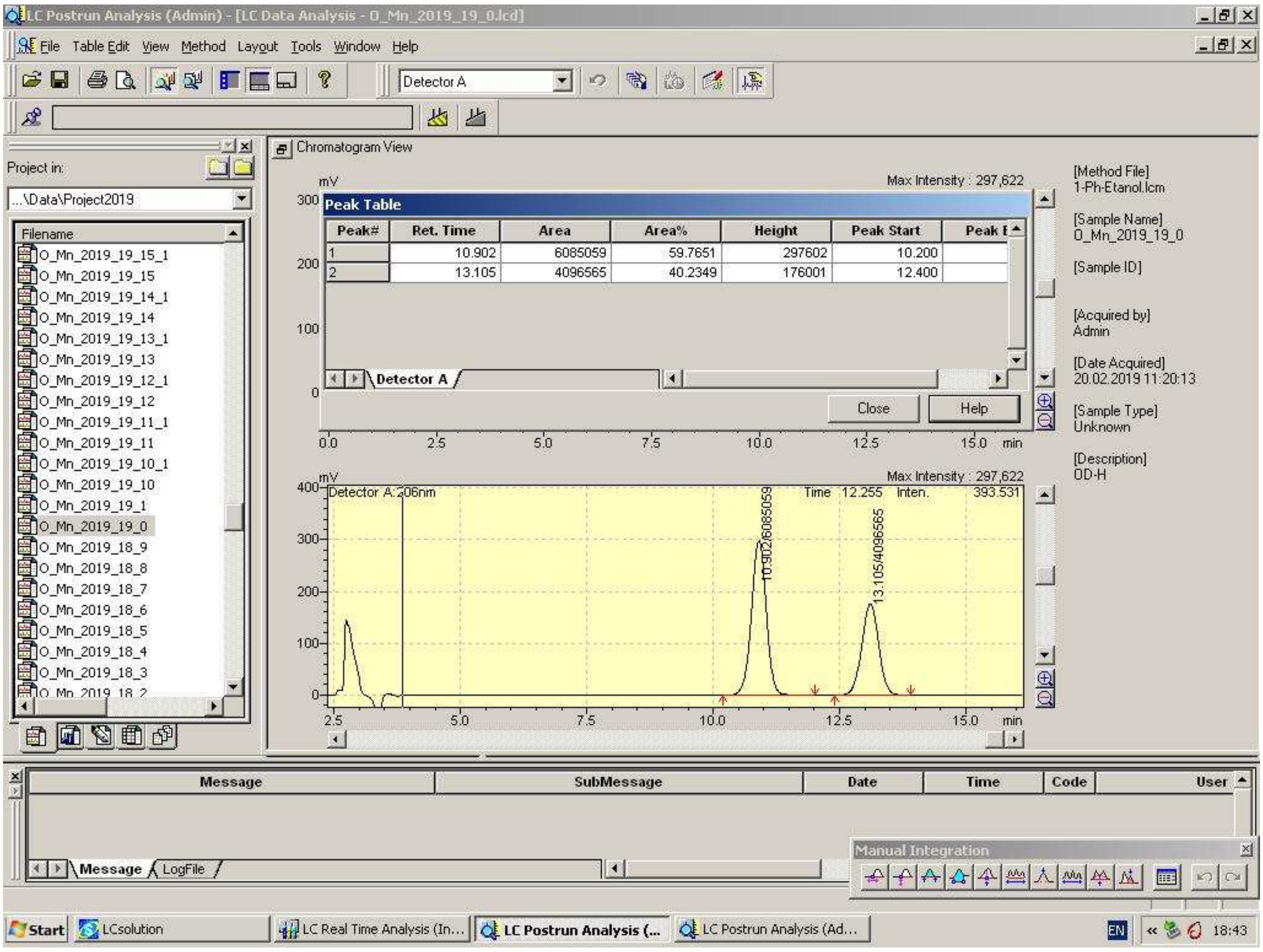

**Table 1, Entry 8 / Figure 3A, violet trace, 0th point**			
Time, min	Area, %	Assignment	Amount(%)
10.902	59.7651	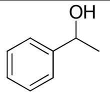 (*R*)-	100 %, 19.5 % *ee* (*R*)-
13.105	40.2349	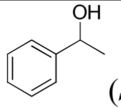 (*S*)-	

Conditions: Chiralcel OD-H, *i*-PrOH/ hexane 3:97, 1.15 mL/min, *λ* 206 nm, 20 °C.

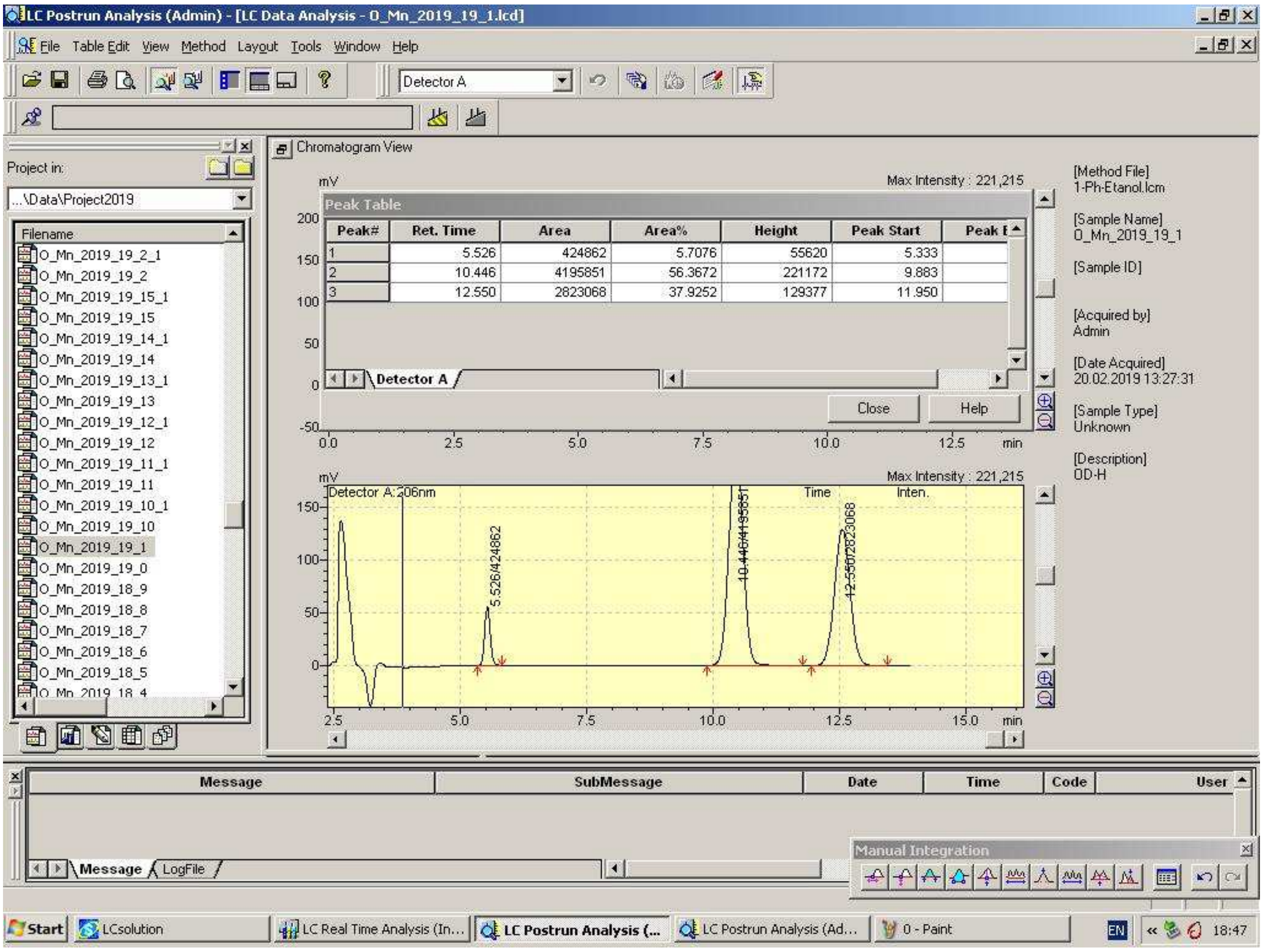

Table 1, Entry 8 / Figure 3A, violet trace, 1st point			
Time, min	Area, %	Assignment	Amount(%)
5.526	5.7076	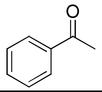	6.5 %
10.446	56.3872	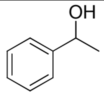 (*R*)-	93.5 %, 19.6 % *ee*
12.550	37.9252	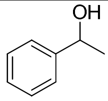 (*S*)-	

Conditions: Chiralcel OD-H, *i*-PrOH/ hexane 3:97, 1.15 mL/min, *λ* 206 nm, 20 °C.

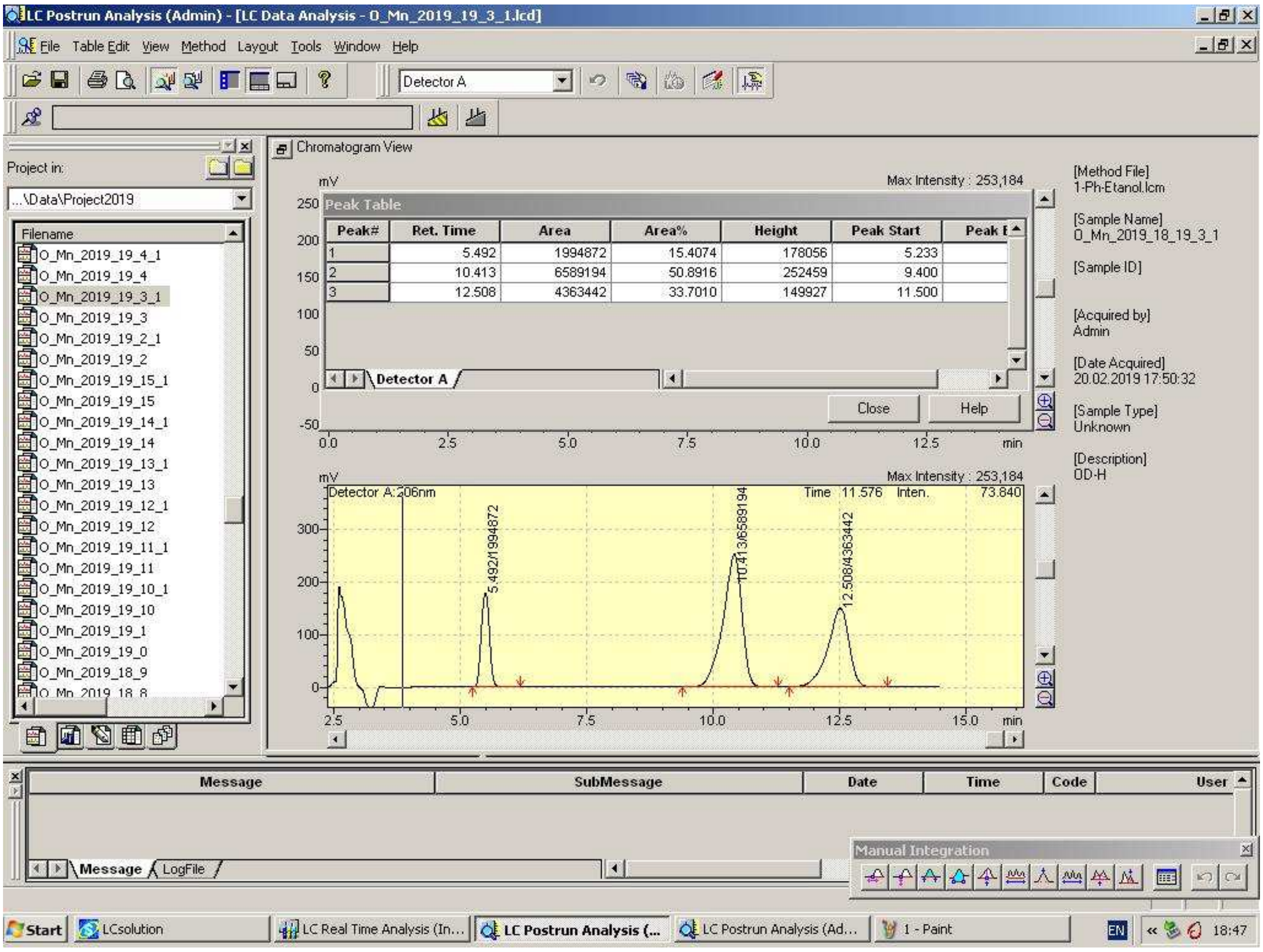

Table 1, Entry 8 / Figure 3A, violet trace, 2nd point			
Time, min	Area, %	Assignment	Amount(%)
5.492	15.4074	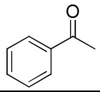	17.3 %
10.413	50.8916	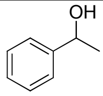 (*R*)-	82.7 %, 20.3 % *ee*
12.508	33.7010	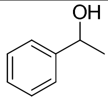 (*S*)-	

Conditions: Chiralcel OD-H, *i*-PrOH/ hexane 3:97, 1.15 mL/min, *λ* 206 nm, 20 °C.

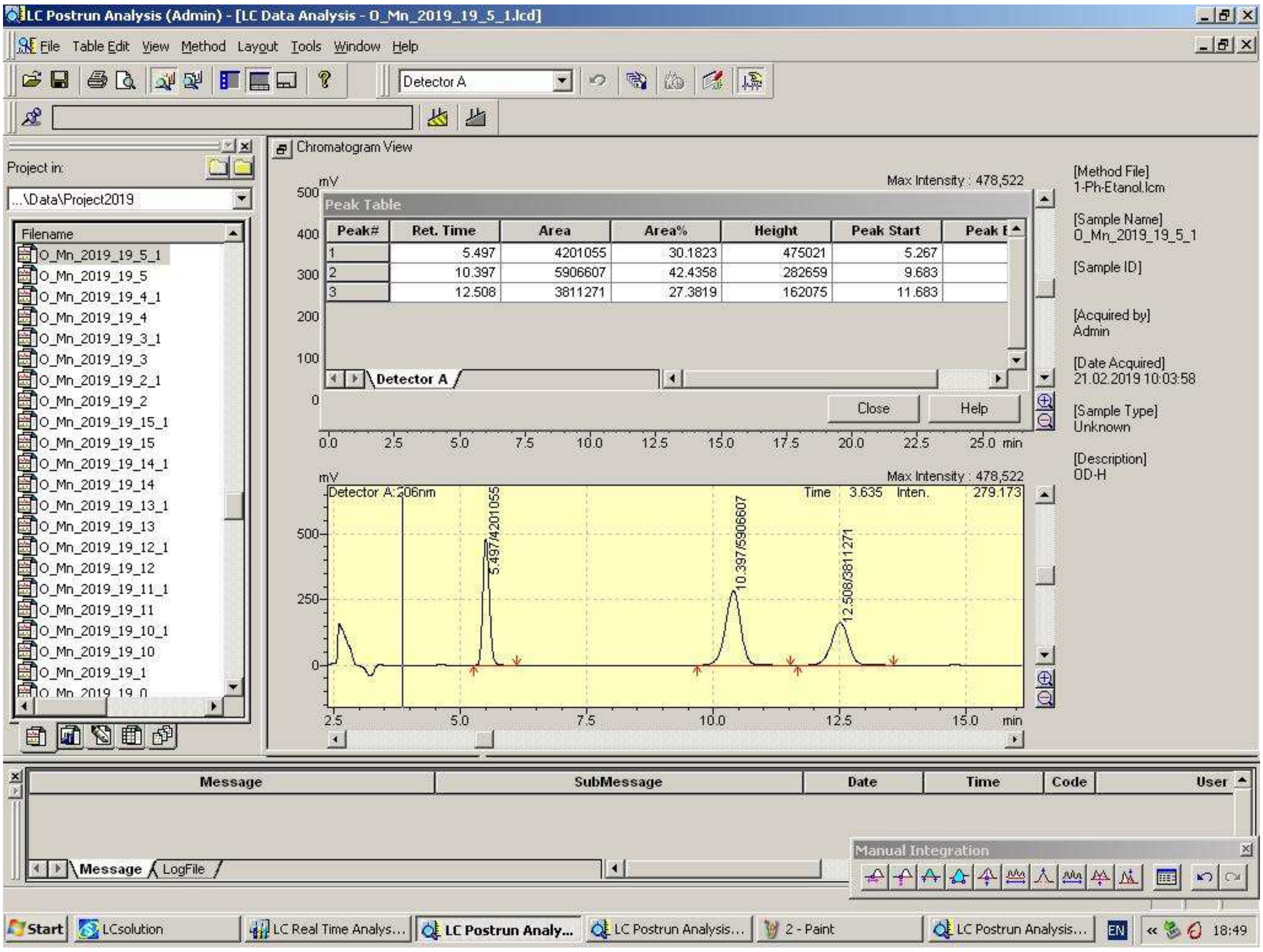

**Table 1, Entry 8 / Figure 3A, violet trace, 3rd point**			
Time, min	Area, %	Assignment	Amount(%)
5.497	30.1823	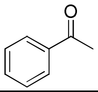	33.2 %
10.397	42.4358	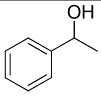 (*R*)-	66.8 %, 21.6 % *ee*
12.508	27.3819	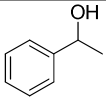 (*S*)-	

Conditions: Chiralcel OD-H, *i*-PrOH/ hexane 3:97, 1.15 mL/min, λ 206 nm, 20 °C.

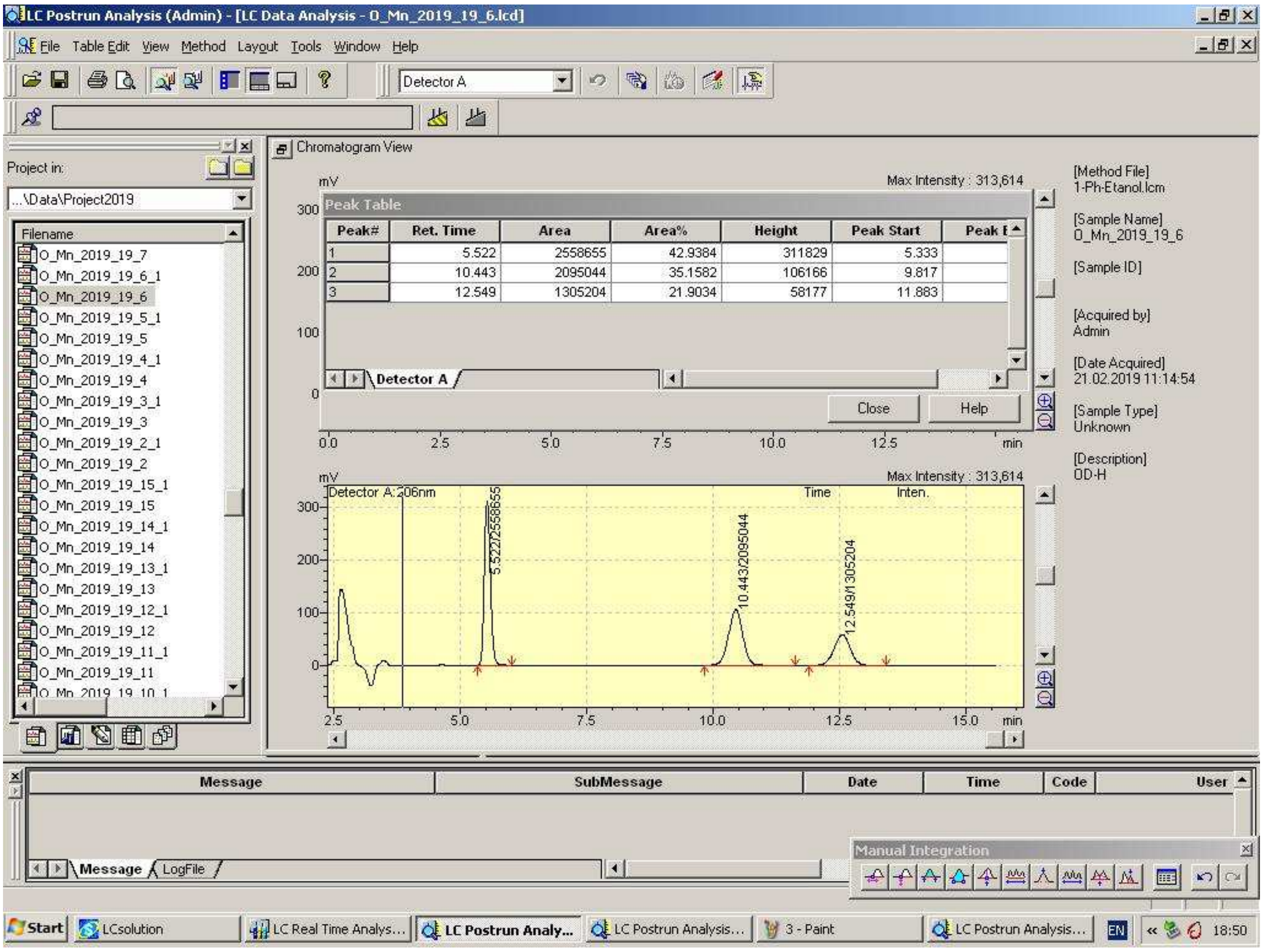

Table 1, Entry 8 / Figure 3A, violet trace, 4th point			
Time, min	Area, %	Assignment	Amount(%)
5.522	42.9384	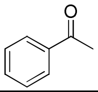	46.3 %
10.443	35.1582	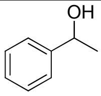 (*R*)-	53.7 %, 23.2 % *ee*
12.549	21.9034	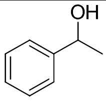 (*S*)-	

Conditions: Chiralcel OD-H, *i*-PrOH/ hexane 3:97, 1.15 mL/min, *λ* 206 nm, 20 °C.

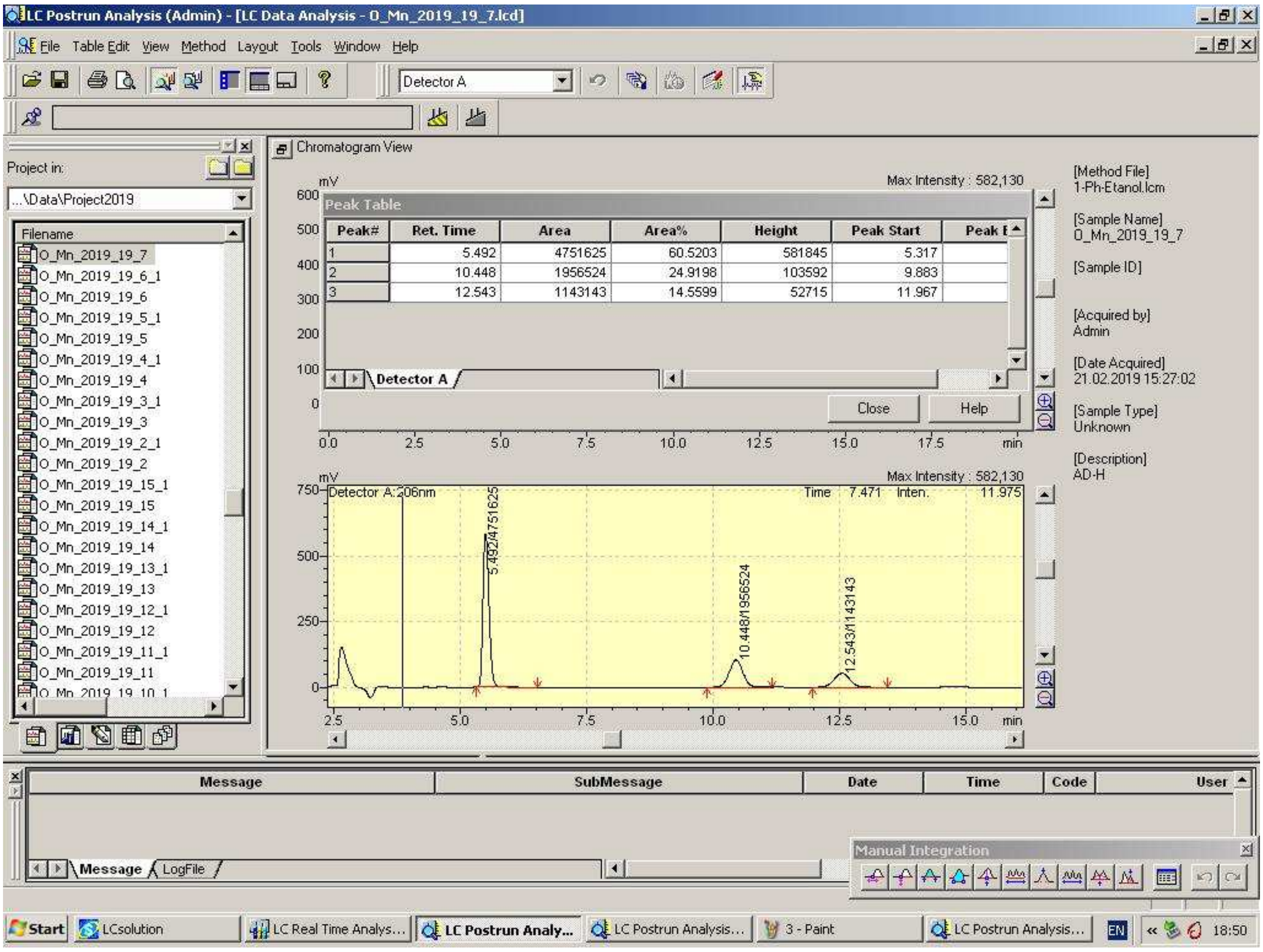

Table 1, Entry 8 / Figure 3A, violet trace, 5th point			
Time, min	Area, %	Assignment	Amount(%)
5.492	60.5203	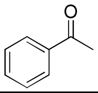	63.7 %
10.448	24.9198	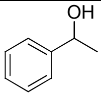 (*R*)-	36.2 %, 26.2 % *ee*
12.543	14.5599	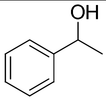 (*S*)-	

Conditions: Chiralcel OD-H, *i*-PrOH/ hexane 3:97, 1.15 mL/min, λ 206 nm, 20 °C.

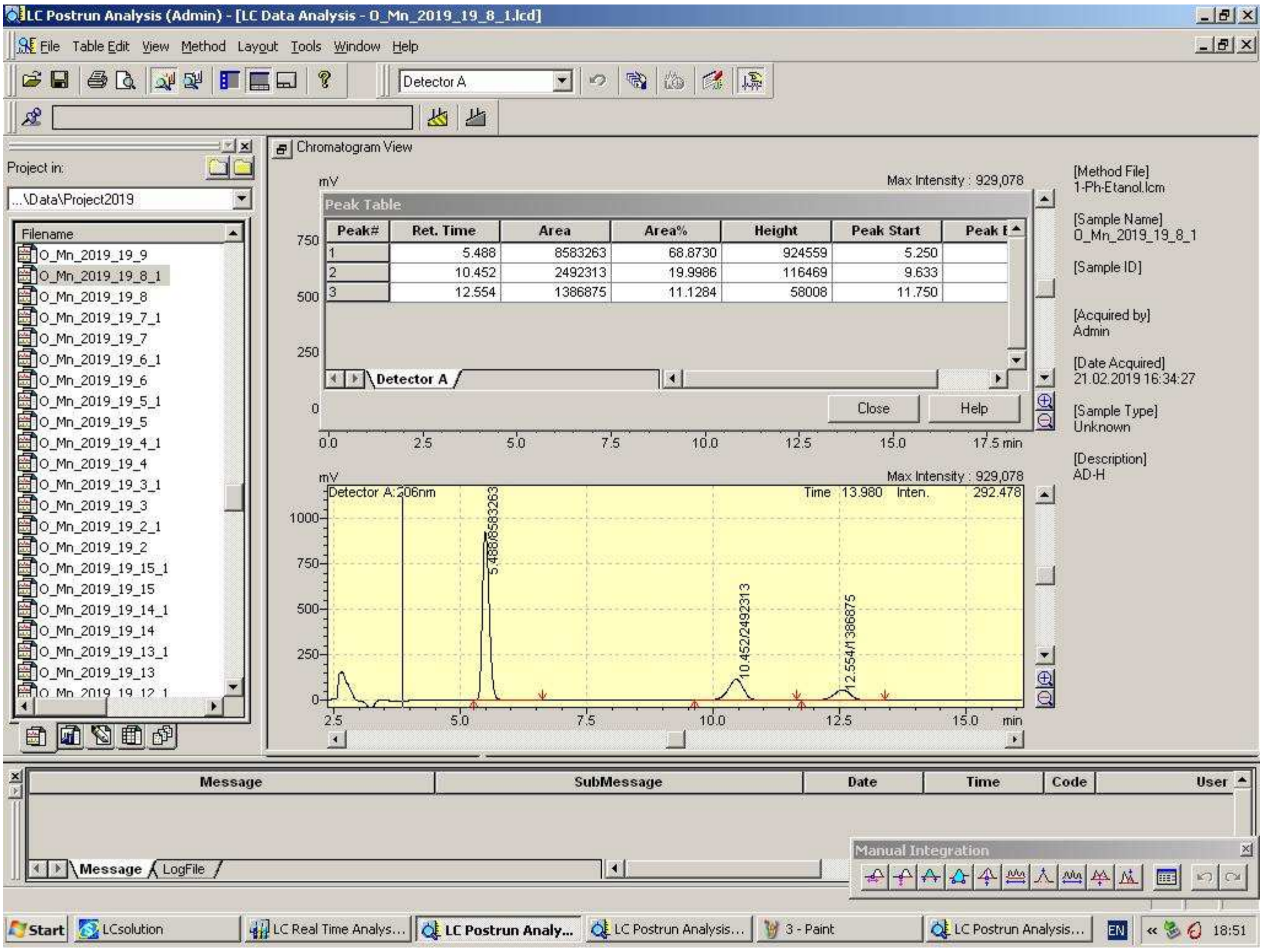

Table 1, Entry 8 / Figure 3A, violet trace, 6th point			
Time, min	Area, %	Assignment	Amount(%)
5.488	68.8730	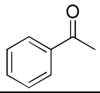	71.7 %
10.452	19.9986	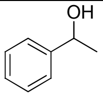 (*R*)-	28.3 %, 28.5 % *ee*
12.554	11.1284	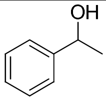 (*S*)-	

Conditions: Chiralcel OD-H, *i*-PrOH/ hexane 3:97, 1.15 mL/min, *λ* 206 nm, 20 °C.

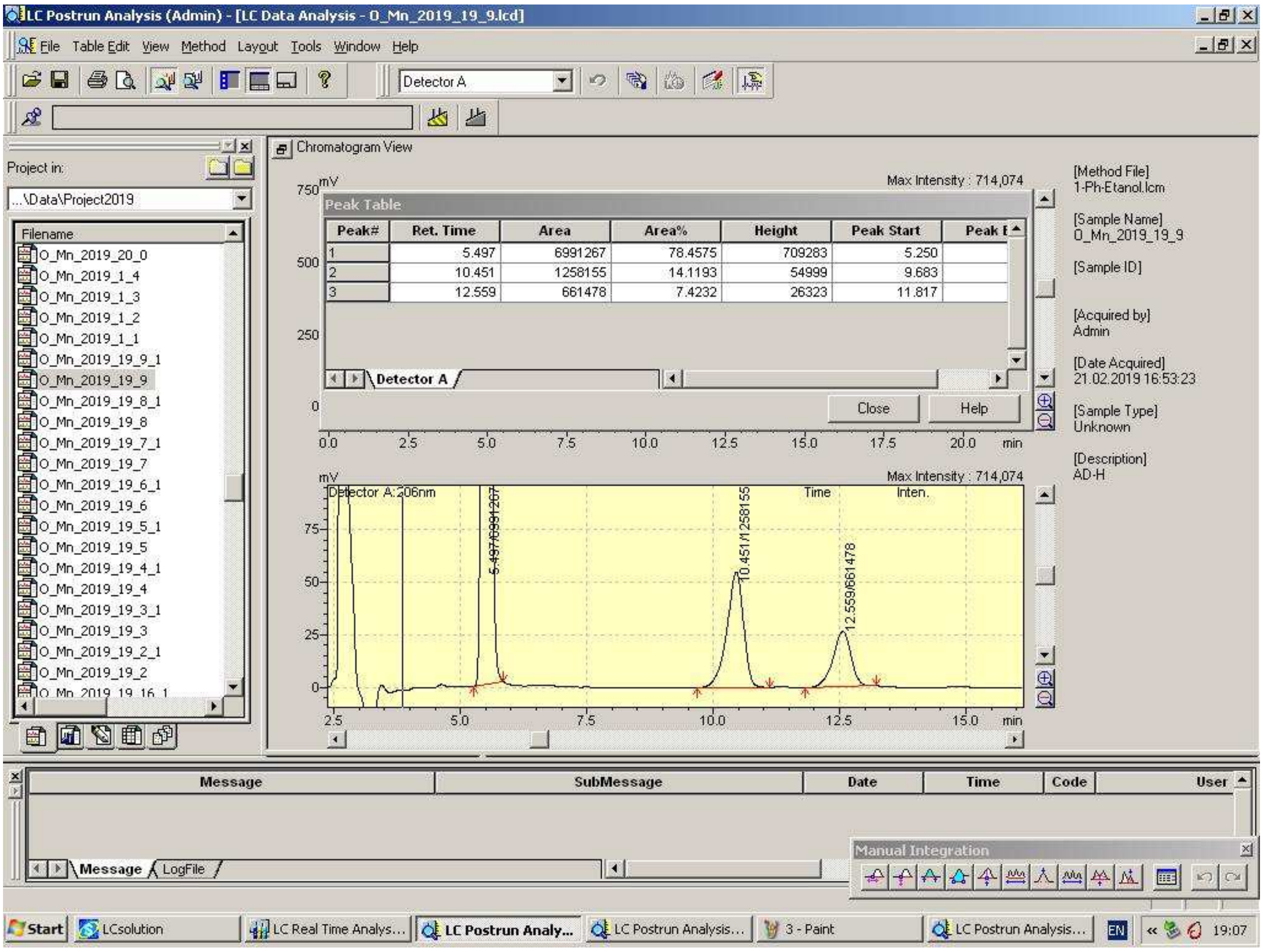

**Table 1, Entry 8 / Figure 3A, violet trace, 7th point**			
Time, min	Area, %	Assignment	Amount(%)
5.497	78.4575	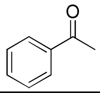	80.8 %
10.451	14.1193	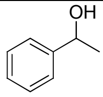 (*R*)-	19.2 %, 31.1 % *ee*
12.559	7.4232	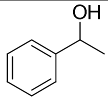 (*S*)-	

Conditions: Chiralcel OD-H, *i*-PrOH/ hexane 3:97, 1.15 mL/min, *λ* 206 nm, 20 °C.

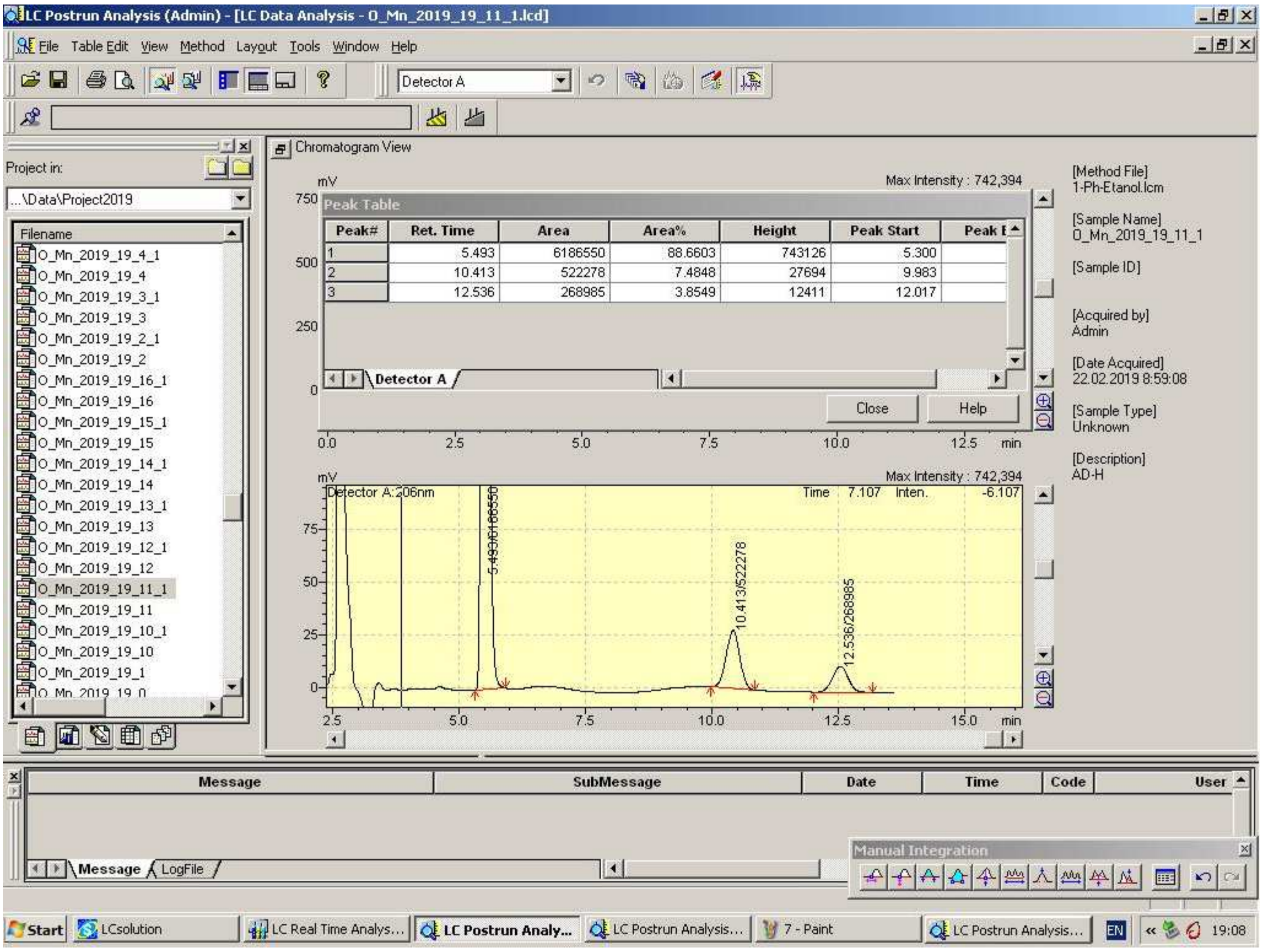

Table 1, Entry 8 / Figure 3A, violet trace, 8th point			
Time, min	Area, %	Assignment	Amount(%)
5.493	88.6603	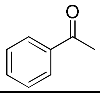	89.9 %
10.413	7.4848	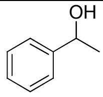 (*R*)-	10.1 %, 35.7 % *ee*
12.536	3.8549	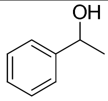 (*S*)-	

Conditions: Chiralcel OD-H, *i*-PrOH/ hexane 3:97, 1.15 mL/min, λ 206 nm, 20 °C.

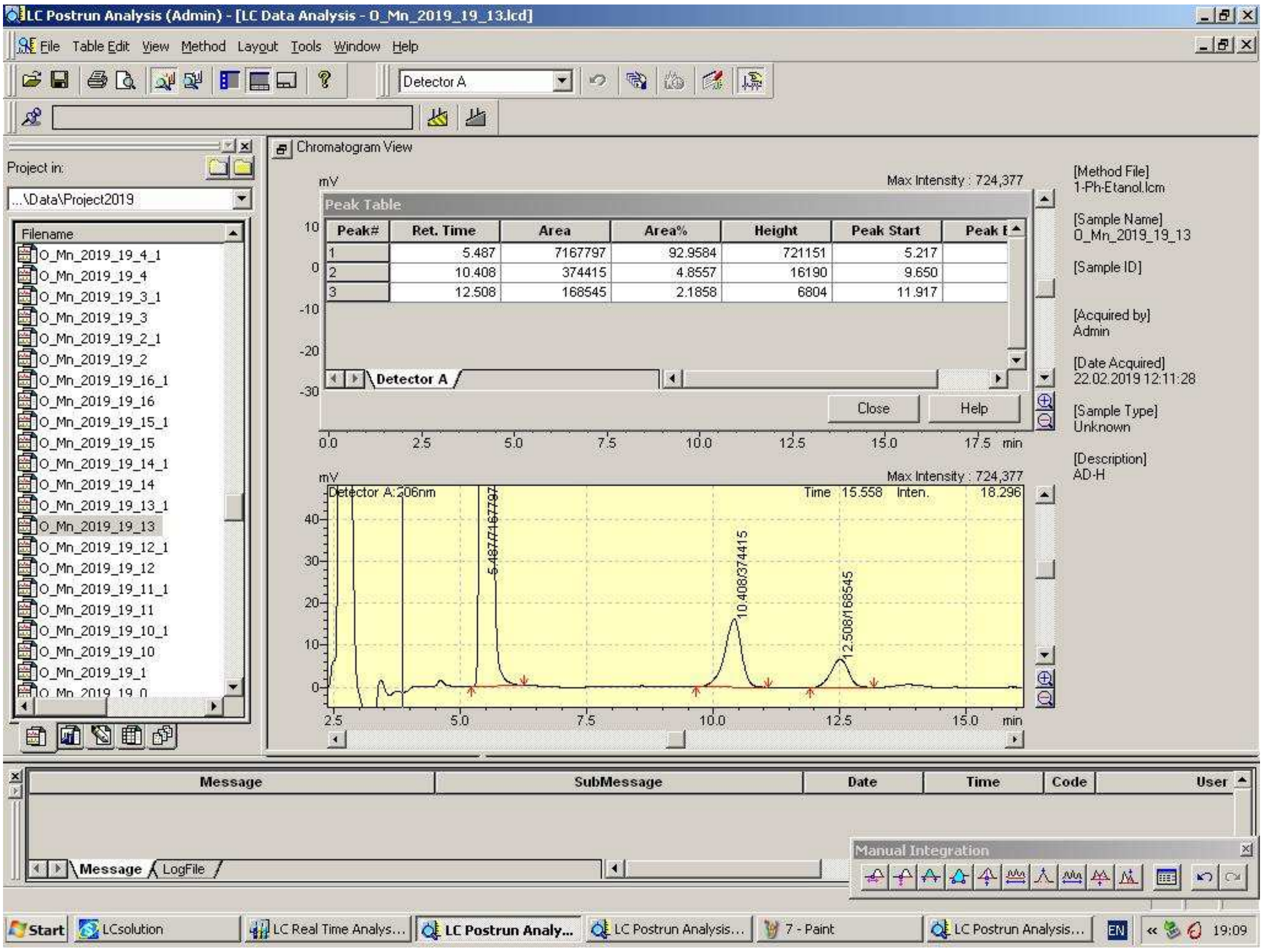

Table 1, Entry 8 / Figure 3A, violet trace, 9th point			
Time, min	Area, %	Assignment	Amount(%)
5.487	92.9584	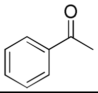	93.8 %
10.408	4.8557	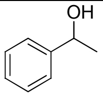 (*R*)-	6.2 %, 37.9 % *ee*
12.508	2.1858	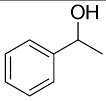 (*S*)-	

Conditions: Chiralcel OD-H, *i*-PrOH/ hexane 3:97, 1.15 mL/min, *λ* 206 nm, 20 °C.

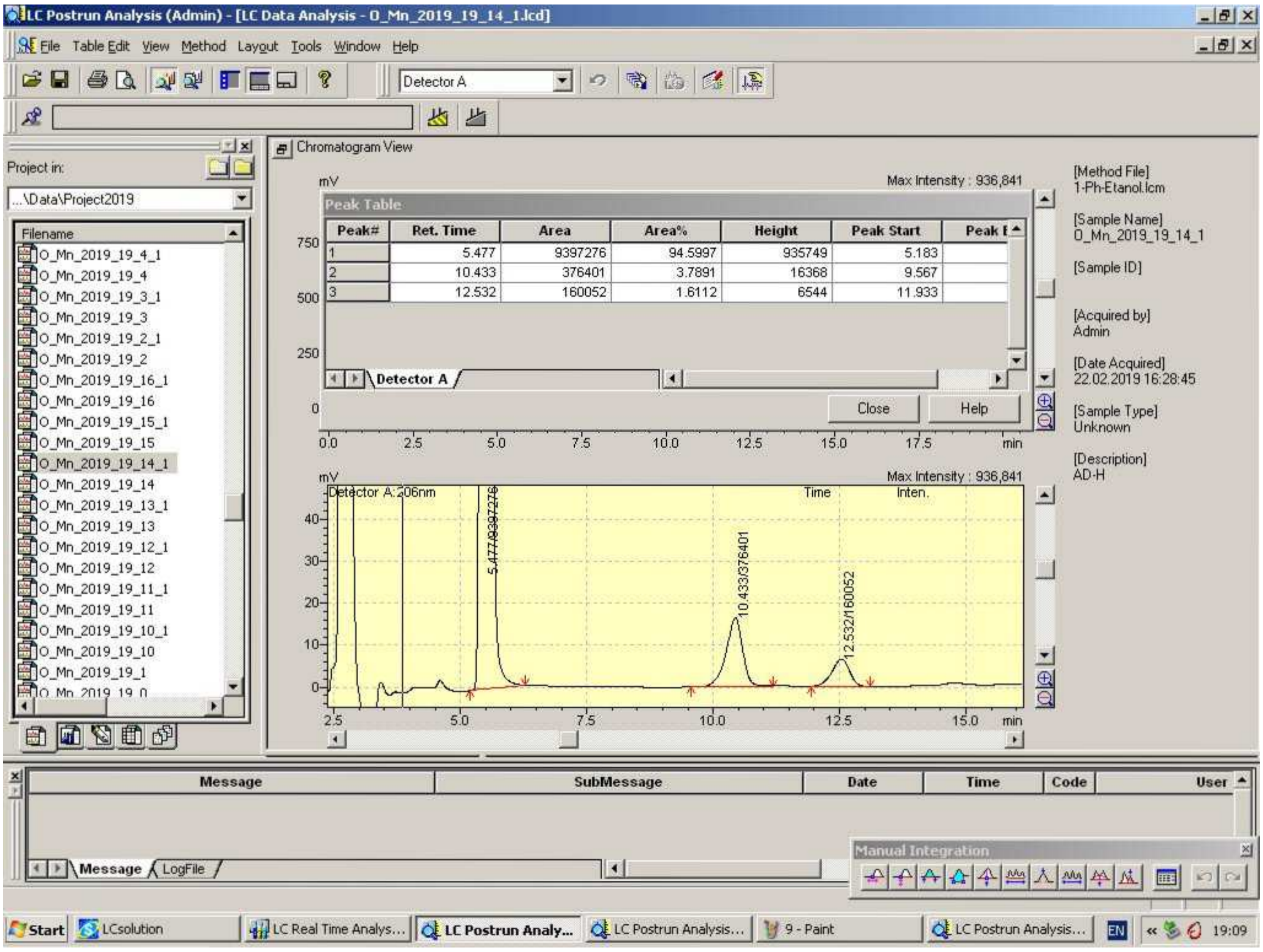

Table 1, Entry 8 / Figure 3A, violet trace, 10th point			
Time, min	Area, %	Assignment	Amount(%)
5.477	94.5997	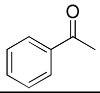	95.2 %
10.433	3.7891	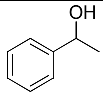 (*R*)-	4.8 %, 40.3 % *ee*
12.532	1.6112	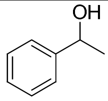 (*S*)-	

Conditions: Chiralcel OD-H, *i*-PrOH/ hexane 3:97, 1.15 mL/min, λ 206 nm, 20 °C.

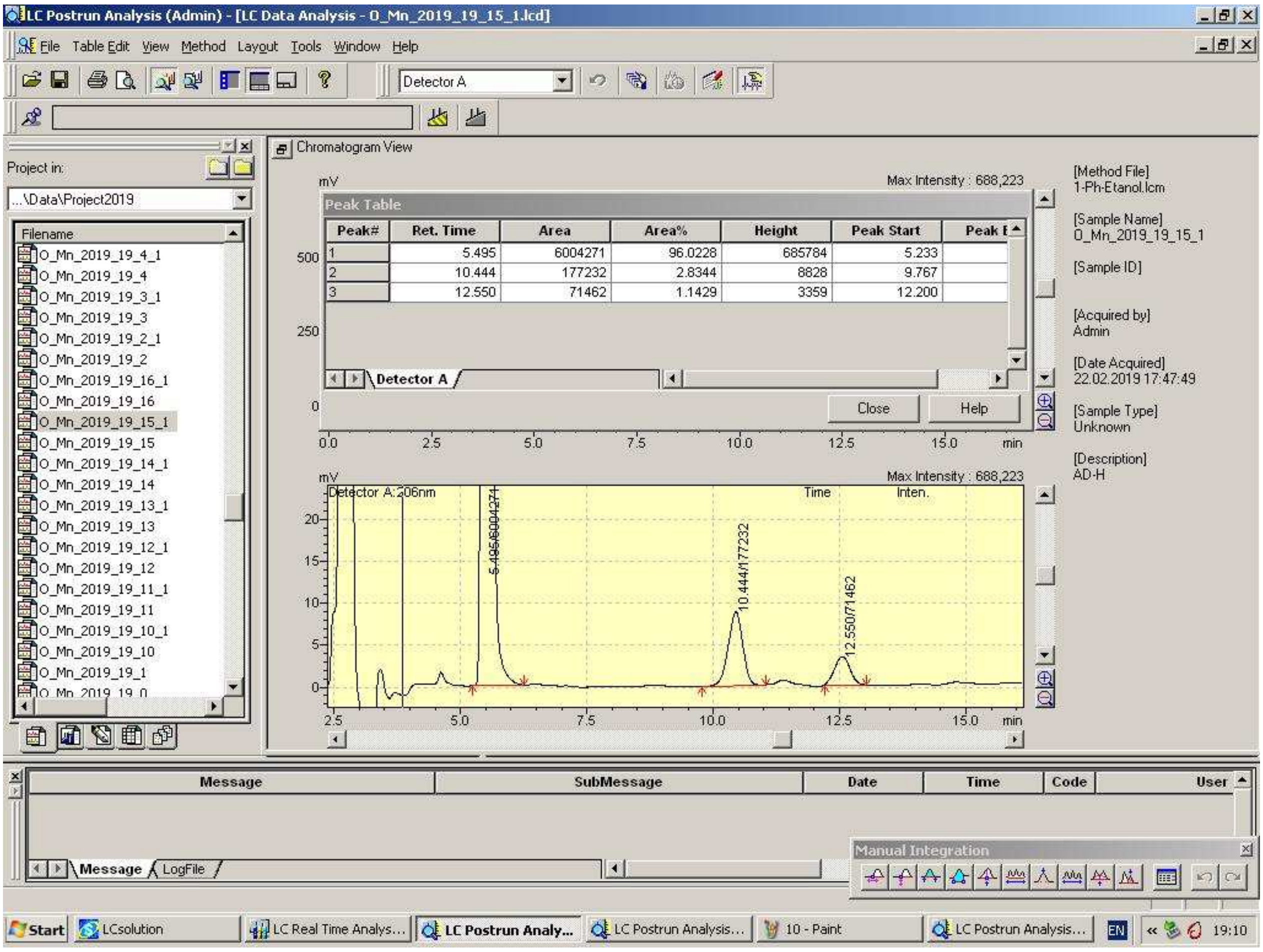

Table 1, Entry 8 / Figure 3A, violet trace, 11th point			
Time, min	Area, %	Assignment	Amount(%)
5.495	96.0228	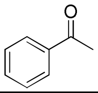	96.5 %
10.444	2.8344	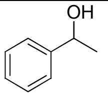 (*R*)-	3.5 %, 42.5 % *ee*
12.550	1.1429	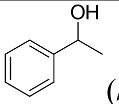 (*S*)-	

Conditions: Chiralcel OD-H, *i*-PrOH/ hexane 3:97, 1.15 mL/min, λ 206 nm, 20 °C.

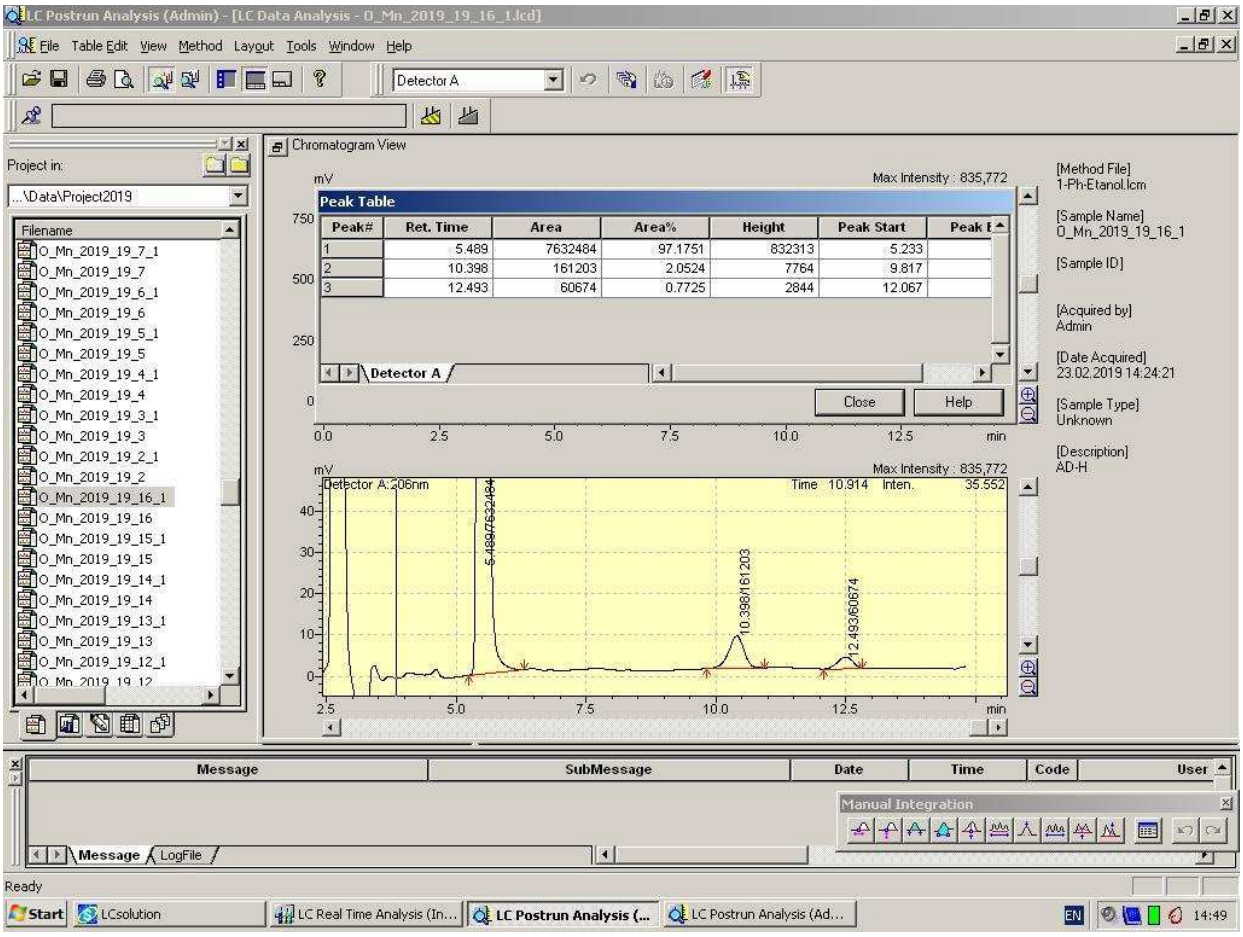

Table 1, Entry 8 / Figure 3A, violet trace, 12th point			
Time, min	Area, %	Assignment	Amount(%)
5.489	97.1751	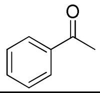	97.5 %
10.398	2.0524	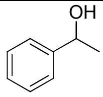 (*R*)-	2.5 %, 45.3 % *ee*
12.493	0.7725	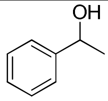 (*S*)-	

Conditions: Chiralcel OD-H, *i*-PrOH/ hexane 3:97, 1.15 mL/min, *λ* 206 nm, 20 °C.

DFT optimized Cartesian coordinates

^{5}[Λ-Cat·R] in CH_3CN

7	0.21853	0.6153	2.17984
25	-0.13139	-0.39284	0.09932
7	-2.20495	-0.44218	0.1829
7	-0.60932	-1.67033	-1.77693
7	-0.02077	-2.35169	1.0338
7	1.9067	-0.74106	-0.08491
8	-6.32001	-0.50717	0.24462
8	5.89933	-1.63682	-0.50221
6	-2.88697	0.0941	1.21426
6	-4.26802	0.11342	1.27892
6	-4.95845	-0.46748	0.20088
6	-4.26912	-1.05856	-0.86788
6	-2.86822	-1.01078	-0.84095
6	-2.06686	-1.54039	-2.01276
6	-4.99246	0.70158	2.45751
6	-7.00567	0.56473	-0.43828
6	-5.01261	-1.72161	-1.99738
6	0.13557	-1.25742	-2.98816
6	-0.21923	-3.04289	-1.37194
6	-0.62498	-3.32673	0.06204
6	-0.76119	-2.47041	2.32508
6	1.4248	-2.66854	1.29981
6	2.39254	-1.86505	0.47365
6	3.73999	-2.22297	0.365
6	4.58004	-1.33935	-0.33166
6	4.07526	-0.17061	-0.92705
6	2.7219	0.07711	-0.77369
6	4.27209	-3.50137	0.95821
6	6.77714	-1.27183	0.58428
6	4.95923	0.75723	-1.71348
8	-0.03902	1.16789	-0.81181
6	0.43593	4.37855	0.59942
6	1.37687	5.40843	0.65119
6	2.27322	5.58786	-0.40002
6	2.22008	4.73553	-1.50449
6	1.27572	3.71305	-1.55552
6	0.37306	3.52131	-0.502
6	-0.67728	2.4222	-0.56702
6	-1.72316	2.68732	-1.65373
6	0.6392	1.22268	3.06259
1	-2.2905	0.51783	2.01216
1	-2.21462	-0.84643	-2.84425
1	-2.48032	-2.49614	-2.34921
1	-5.69393	-0.02014	2.88118
1	-4.29162	1.00088	3.23741
1	-5.57233	1.58281	2.17224
1	-6.77277	0.55842	-1.50511
1	-8.0668	0.37922	-0.29115
1	-6.73602	1.53157	-0.00873
1	-6.07324	-1.79179	-1.76769
1	-4.64564	-2.73497	-2.1758
1	-4.9017	-1.16694	-2.93366
1	-0.0741	-0.21045	-3.19703
1	-0.14373	-1.86764	-3.85425
1	1.20454	-1.36834	-2.81529
1	0.86166	-3.13118	-1.48744
1	-0.67098	-3.79339	-2.03124
1	-1.70699	-3.26321	0.17274
1	-0.33798	-4.34417	0.3417
1	-1.82049	-2.28998	2.16273
1	-0.63651	-3.476	2.73607
1	-0.37888	-1.74305	3.03608
1	1.58572	-3.74135	1.17801
1	1.62262	-2.44929	2.35154
1	2.24543	0.94974	-1.20007
1	4.31808	-3.45214	2.0502
1	5.27348	-3.70689	0.58615
1	3.64225	-4.35473	0.69763
1	6.49476	-1.78507	1.50578
1	7.77261	-1.58471	0.27882
1	6.76173	-0.19196	0.74534
1	5.72882	1.20769	-1.0818
1	5.47499	0.22017	-2.51251
1	4.37738	1.5643	-2.15924
1	-0.25838	4.24546	1.42392
1	1.40922	6.0665	1.51356
1	3.00738	6.38593	-0.3619
1	2.91277	4.87101	-2.32893
1	1.23979	3.05994	-2.42132
1	-1.18407	2.38937	0.40417
1	-2.20144	3.65734	-1.49478
1	-2.49874	1.91877	-1.63986
1	-1.259	2.69171	-2.64269
6	1.16396	1.9878	4.17503
1	0.4386	2.00209	4.98991
1	1.36415	3.01159	3.85518
1	2.09157	1.5357	4.52957

^{5}[Λ-Cat·S] in CH_3CN

25	0.40435	-0.28999	0.19183
7	2.43301	0.05722	-0.06331
7	1.04617	-2.30574	-0.3097
7	0.02008	-0.18692	-2.09387
7	-1.58747	-0.87941	0.10687
8	6.44483	0.62477	-0.77056
8	-5.52239	-2.0574	-0.1721
6	2.96351	1.29234	-0.02197
6	4.30929	1.53653	-0.23557
6	5.12203	0.42227	-0.50966
6	4.58225	-0.87126	-0.58458
6	3.21192	-1.00401	-0.33952
6	2.54992	-2.35446	-0.28835
6	4.86827	2.93201	-0.2025
6	7.33702	0.49188	0.35639
6	5.443	-2.06112	-0.91863
6	0.53789	-3.36786	0.60814
6	0.50785	-2.58706	-1.68486
6	0.67317	-1.40889	-2.62582
6	0.57054	1.01577	-2.75904
6	-1.45335	-0.2077	-2.24808
6	-2.21421	-0.80383	-1.08233
6	-3.55385	-1.18812	-1.23013
6	-4.22927	-1.6351	-0.0851
6	-3.57423	-1.7284	1.15511
6	-2.24883	-1.33509	1.18907
6	-4.24683	-1.13196	-2.56659
6	-6.53467	-1.03716	-0.03251
6	-4.26817	-2.25776	2.37889
7	0.71408	-0.73114	2.47042
8	0.06965	1.46542	0.48113
6	1.05066	-0.71717	3.57115
6	1.46843	-0.70422	4.9583
6	-2.01231	2.70987	0.56956
1	-1.29672	1.32675	2.02793
1	-0.81508	3.59852	2.90975
6	-0.12698	3.11182	2.21393
6	-1.75139	3.5553	-0.51571
6	-2.7951	4.15131	-1.21961
1	2.25967	2.08748	0.1885
1	1.43591	-1.71634	5.36438
1	2.85353	-2.83934	0.64204
1	4.06935	3.67007	-0.12564
1	5.44744	3.14035	-1.10429
1	5.53786	3.07567	0.6495
1	7.29941	-0.52018	0.76518
1	8.33319	0.69761	-0.02767
1	7.08539	1.2131	1.1365
1	5.00779	-2.65276	-1.72748
1	5.56242	-2.72551	-0.05776
1	6.43162	-1.74069	-1.23963
1	-0.54863	-3.37971	0.59463
1	0.88067	-3.17369	1.62077
1	0.90377	-4.34574	0.28365
1	-0.54461	-2.84387	-1.57306
1	1.00696	-3.46878	-2.0965
1	0.27143	-1.67472	-3.61061
1	1.73052	-1.18281	-2.76819
1	1.65333	1.03232	-2.64955
1	0.1605	1.90503	-2.28611
1	0.32642	1.02494	-3.82717
1	-1.79125	0.8273	-2.34775
1	-1.72837	-0.70843	-3.18079
1	-1.68219	-1.38584	2.11019
1	-4.36902	-0.10204	-2.9139
1	-5.23094	-1.59069	-2.50715
1	-3.68016	-1.66845	-3.3313
1	-6.45229	-0.29554	-0.82964
1	-7.48825	-1.55413	-0.10692
1	-6.45642	-0.5435	0.93811
1	-4.72714	-3.22733	2.17512
1	-3.56693	-2.37359	3.20592
1	-5.06637	-1.58807	2.70873
6	-0.87227	2.08246	1.35711
6	-4.11914	3.91495	-0.84494
6	-3.34065	2.47936	0.93606
6	-4.38928	3.07937	0.23696
1	0.32528	3.88149	1.58398
1	0.66315	2.62491	2.78976
1	-2.5764	4.80321	-2.05931
1	-0.72503	3.74758	-0.80998
1	-5.41507	2.89025	0.53657
1	-4.93236	4.37995	-1.3925
1	-3.56085	1.82706	1.77618
1	2.48711	-0.32068	5.03363
1	0.80193	-0.06413	5.53834
1	2.90162	-3.00221	-1.09396

^{3}[Λ-Cat·R] in CH_3CN

7	0.24078	0.46828	1.80311
25	-0.13891	-0.41965	0.02331
7	-2.15798	-0.41655	0.19851
7	-0.53622	-1.48114	-1.73526
7	-0.04144	-2.36943	0.95896
7	1.87739	-0.74626	-0.1361
8	-6.27778	-0.55113	0.27045
8	5.90673	-1.56716	-0.4271
6	-2.84618	0.03975	1.26232
6	-4.22848	0.03423	1.33046
6	-4.91732	-0.4844	0.22087
6	-4.22436	-0.991	-0.88885
6	-2.82707	-0.92143	-0.85564
6	-1.99531	-1.32909	-2.04176
6	-4.9564	0.53609	2.54642
6	-6.98976	0.53466	-0.36102
6	-4.95905	-1.58903	-2.05974
6	0.23427	-1.01986	-2.92448
6	-0.17475	-2.90741	-1.45415
6	-0.61558	-3.30355	-0.0634
6	-0.803	-2.54424	2.22881
6	1.39975	-2.68012	1.23949
6	2.37173	-1.85551	0.43784
6	3.72993	-2.18849	0.37261
6	4.57552	-1.29069	-0.29456
6	4.06638	-0.12816	-0.89533
6	2.7028	0.09183	-0.78621
6	4.26321	-3.45729	0.98506
6	6.74522	-1.16511	0.67538
6	4.9543	0.82395	-1.64837
8	-0.08427	1.13625	-0.89018
6	0.3263	4.28598	0.72595
6	1.2679	5.3035	0.89101
6	2.2467	5.51193	-0.07815
6	2.27413	4.70175	-1.21468
6	1.32921	3.69153	-1.37822
6	0.34521	3.46843	-0.40708
6	-0.70751	2.38503	-0.59575
6	-1.68801	2.73516	-1.71992
6	0.56825	1.01327	2.76078
1	-2.25749	0.41964	2.08581
1	-2.09347	-0.54952	-2.80068
1	-2.37225	-2.24683	-2.4982
1	-5.63916	-0.22403	2.93188
1	-4.25605	0.80406	3.33812
1	-5.55698	1.41989	2.31737
1	-6.74793	0.59418	-1.42411
1	-8.04599	0.30937	-0.23542
1	-6.75401	1.48422	0.12366
1	-6.00947	-1.73481	-1.8175
1	-4.54555	-2.56155	-2.33575
1	-4.8997	-0.94742	-2.94387
1	0.05522	0.0416	-3.06824
1	-0.07694	-1.57967	-3.81066
1	1.2955	-1.18268	-2.75892
1	0.90623	-2.99637	-1.5548
1	-0.62402	-3.56677	-2.20167
1	-1.70075	-3.26164	0.02517
1	-0.31616	-4.33242	0.15429
1	-1.86117	-2.37205	2.05386
1	-0.67203	-3.56164	2.60783
1	-0.44459	-1.83901	2.97393
1	1.57299	-3.74998	1.1052
1	1.58172	-2.47409	2.29686
1	2.22837	0.96043	-1.22094
1	4.26353	-3.40985	2.07821
1	5.28315	-3.64161	0.65444
1	3.66177	-4.32302	0.69865
1	6.45667	-1.67906	1.59495
1	7.75788	-1.44887	0.39847
1	6.69346	-0.08486	0.82764
1	5.69662	1.28521	-0.99222
1	5.50372	0.30419	-2.43653
1	4.36963	1.623	-2.10497
1	-0.43379	4.1316	1.48669
1	1.23632	5.92889	1.77744
1	2.98203	6.29978	0.04831
1	3.03068	4.86056	-1.97653
1	1.35647	3.07267	-2.26919
1	-1.2722	2.30182	0.33896
1	-2.17174	3.69537	-1.52208
1	-2.46563	1.97247	-1.80255
1	-1.16793	2.80468	-2.67831
6	0.97414	1.69808	3.96941
1	0.10045	1.91166	4.587
1	1.46851	2.63551	3.70954
1	1.66844	1.07159	4.53162

^{3}[Λ-Cat·S] in CH_3CN

25	0.394	-0.30516	0.11772
7	2.39435	0.05534	-0.09857
7	1.04039	-2.31991	-0.34461
7	0.05917	-0.13235	-1.93983
7	-1.54102	-0.91652	0.13751
8	6.43657	0.6477	-0.64763
8	-5.46794	-2.13355	-0.17662
6	2.92252	1.28902	-0.01697
6	4.27592	1.54065	-0.17241
6	5.104	0.4362	-0.43357
6	4.57105	-0.85606	-0.54035
6	3.19142	-0.99587	-0.34644
6	2.54206	-2.35457	-0.31824
6	4.82435	2.93883	-0.09358
6	7.28841	0.50076	0.50668
6	5.44618	-2.0412	-0.85388
6	0.53994	-3.40761	0.54378
6	0.50914	-2.56398	-1.72446
6	0.68266	-1.33283	-2.58503
6	0.63189	1.09292	-2.56521
6	-1.4252	-0.09577	-2.13417
6	-2.19035	-0.78102	-1.03516
6	-3.52288	-1.17272	-1.20094
6	-4.1796	-1.69831	-0.07813
6	-3.50919	-1.8537	1.14697
6	-2.18704	-1.44571	1.19349
6	-4.225	-1.04601	-2.52759
6	-6.49071	-1.13158	0.00935
6	-4.18617	-2.45929	2.34503
7	0.7456	-0.61562	2.08976
8	-0.00494	1.43815	0.35957
6	1.03316	-0.69718	3.19977
6	1.39302	-0.8021	4.59774
6	-2.03856	2.73229	0.58344
1	-1.31	1.27635	1.95756
1	-0.73568	3.49945	2.90884
6	-0.08666	3.02504	2.16804
6	-1.79617	3.63602	-0.45852
6	-2.85028	4.28091	-1.10125
1	2.21191	2.08154	0.17565
1	2.16846	-1.55979	4.72158
1	2.84505	-2.84773	0.60804
1	4.01817	3.67025	-0.02824
1	5.43128	3.16956	-0.97149
1	5.46505	3.07021	0.78237
1	7.24021	-0.51715	0.90004
1	8.29748	0.71417	0.1618
1	7.00859	1.20964	1.28876
1	5.03789	-2.63204	-1.67732
1	5.54469	-2.70938	0.00698
1	6.44228	-1.71469	-1.14452
1	-0.54585	-3.43621	0.52065
1	0.87217	-3.23325	1.56374
1	0.92336	-4.37314	0.20272
1	-0.54297	-2.82955	-1.63049
1	1.0161	-3.42047	-2.17786
1	0.25407	-1.49493	-3.57752
1	1.74037	-1.11307	-2.72472
1	1.71619	1.06448	-2.50394
1	0.26683	1.96352	-2.0294
1	0.33741	1.14825	-3.61681
1	-1.72596	0.95447	-2.13734
1	-1.67753	-0.49782	-3.11741
1	-1.61463	-1.5458	2.10531
1	-4.39518	8.76E-4	-2.79478
1	-5.18802	-1.55087	-2.50087
1	-3.63942	-1.49654	-3.33231
1	-6.41585	-0.35201	-0.75151
1	-7.43911	-1.65395	-0.09009
1	-6.41724	-0.68397	1.00249
1	-4.64014	-3.41906	2.0903
1	-3.47552	-2.61706	3.15695
1	-4.98603	-1.81613	2.72066
6	-0.88607	2.04512	1.30281
6	-4.16733	4.03512	-0.70863
6	-3.3604	2.49363	0.96837
6	-4.41958	3.14126	0.32996
1	0.35886	3.80967	1.55166
1	0.71486	2.50275	2.69554
1	-2.64493	4.97806	-1.90735
1	-0.77586	3.8375	-0.76762
1	-5.43972	2.94413	0.64355
1	-4.98873	4.53779	-1.20866
1	-3.56703	1.79599	1.77485
1	1.77249	0.15788	4.95169
1	0.51874	-1.08242	5.18703
1	2.9067	-2.98916	-1.12913

^{1}[Λ-Cat·R] in CH_3CN

25	0.3587	-0.26358	0.1448
7	2.36663	0.03712	-0.1099
7	0.99444	-2.32789	-0.23565
7	0.09693	-0.1658	-1.94267
7	-1.56298	-0.90481	0.14461
8	6.42603	0.53724	-0.66589
8	-5.46285	-2.18119	-0.2742
6	2.91667	1.26607	-0.11423
6	4.27369	1.48824	-0.27811
6	5.08885	0.3577	-0.45134
6	4.53739	-0.93029	-0.47199
6	3.15427	-1.03764	-0.27879
6	2.48819	-2.38077	-0.15789
6	4.84088	2.88115	-0.30035
6	7.26621	0.44738	0.50276
6	5.39829	-2.14604	-0.69531
6	0.45531	-3.39424	0.65101
6	0.5126	-2.59549	-1.62871
6	0.72449	-1.39307	-2.52279
6	0.66926	1.02754	-2.62375
6	-1.38454	-0.14243	-2.1536
6	-2.17216	-0.8097	-1.05566
6	-3.49355	-1.22484	-1.25713
6	-4.18426	-1.72674	-0.14437
6	-3.55553	-1.84073	1.10774
6	-2.24108	-1.4171	1.1897
6	-4.14916	-1.15222	-2.61182
6	-6.50421	-1.18718	-0.16412
6	-4.26811	-2.42179	2.29725
7	0.59716	-0.55307	2.12784
8	8.61E-4	1.45542	0.28649
6	0.85775	-0.65974	3.24298
6	1.17523	-0.79582	4.64918
6	-1.93279	2.84126	0.60112
1	-1.21553	1.35639	1.95968
1	-0.46487	3.54249	2.86949
6	0.11536	3.04204	2.09065
6	-1.6834	3.75823	-0.4275
6	-2.73251	4.44181	-1.0372
1	2.22397	2.08632	0.01151
1	1.29114	-1.85129	4.90086
1	2.7558	-2.79467	0.81725
1	4.04553	3.6271	-0.28531
1	5.44727	3.0402	-1.19446
1	5.4872	3.06536	0.56181
1	7.20321	-0.5454	0.95396
1	8.28059	0.62965	0.15561
1	6.98756	1.20304	1.24022
1	4.98849	-2.7867	-1.47972
1	5.4808	-2.75509	0.20994
1	6.40101	-1.85372	-0.99927
1	-0.62929	-3.41552	0.5897
1	0.74979	-3.20279	1.67975
1	0.84119	-4.37171	0.34656
1	-0.54482	-2.84962	-1.56629
1	1.02524	-3.46791	-2.04566
1	0.32438	-1.58986	-3.52172
1	1.78819	-1.18961	-2.63996
1	1.7536	1.00259	-2.56095
1	0.30405	1.9251	-2.13323
1	0.37792	1.03614	-3.67814
1	-1.69038	0.90652	-2.18091
1	-1.62453	-0.56267	-3.13255
1	-1.70105	-1.48506	2.12321
1	-4.31883	-0.11813	-2.92476
1	-5.10798	-1.66548	-2.60036
1	-3.53204	-1.62719	-3.37789
1	-6.41429	-0.43844	-0.95378
1	-7.44171	-1.72696	-0.27347
1	-6.47028	-0.69851	0.8117
1	-4.69861	-3.39535	2.0542
1	-3.58577	-2.54554	3.13886
1	-5.09062	-1.78072	2.62456
6	-0.7907	2.1056	1.28567
6	-4.04759	4.22113	-0.62444
6	-3.25273	2.62331	1.00441
6	-4.30503	3.31209	0.39962
1	0.55683	3.80626	1.44751
1	0.9213	2.48073	2.56794
1	-2.52427	5.14911	-1.83343
1	-0.6647	3.939	-0.75353
1	-5.32411	3.13483	0.72771
1	-4.86463	4.75484	-1.09861
1	-3.46198	1.91338	1.79901
1	2.10555	-0.2705	4.87064
1	0.37121	-0.37082	5.2521
1	2.87103	-3.08717	-0.8987

^{1}[Λ-Cat·S] in CH_3CN

25	0.3587	-0.26358	0.1448
7	2.36663	0.03712	-0.1099
7	0.99444	-2.32789	-0.23565
7	0.09693	-0.1658	-1.94267
7	-1.56298	-0.90481	0.14461
8	6.42603	0.53724	-0.66589
8	-5.46285	-2.18119	-0.2742
6	2.91667	1.26607	-0.11423
6	4.27369	1.48824	-0.27811
6	5.08885	0.3577	-0.45134
6	4.53739	-0.93029	-0.47199
6	3.15427	-1.03764	-0.27879
6	2.48819	-2.38077	-0.15789
6	4.84088	2.88115	-0.30035
6	7.26621	0.44738	0.50276
6	5.39829	-2.14604	-0.69531
6	0.45531	-3.39424	0.65101
6	0.5126	-2.59549	-1.62871
6	0.72449	-1.39307	-2.52279
6	0.66926	1.02754	-2.62375
6	-1.38454	-0.14243	-2.1536
6	-2.17216	-0.8097	-1.05566
6	-3.49355	-1.22484	-1.25713
6	-4.18426	-1.72674	-0.14437
6	-3.55553	-1.84073	1.10774
6	-2.24108	-1.4171	1.1897
6	-4.14916	-1.15222	-2.61182
6	-6.50421	-1.18718	-0.16412
6	-4.26811	-2.42179	2.29725
7	0.59716	-0.55307	2.12784
8	8.61E-4	1.45542	0.28649
6	0.85775	-0.65974	3.24298
6	1.17523	-0.79582	4.64918
6	-1.93279	2.84126	0.60112
1	-1.21553	1.35639	1.95968
1	-0.46487	3.54249	2.86949
6	0.11536	3.04204	2.09065
6	-1.6834	3.75823	-0.4275
6	-2.73251	4.44181	-1.0372
1	2.22397	2.08632	0.01151
1	1.29114	-1.85129	4.90086
1	2.7558	-2.79467	0.81725
1	4.04553	3.6271	-0.28531
1	5.44727	3.0402	-1.19446
1	5.4872	3.06536	0.56181
1	7.20321	-0.5454	0.95396
1	8.28059	0.62965	0.15561
1	6.98756	1.20304	1.24022
1	4.98849	-2.7867	-1.47972
1	5.4808	-2.75509	0.20994
1	6.40101	-1.85372	-0.99927
1	-0.62929	-3.41552	0.5897
1	0.74979	-3.20279	1.67975
1	0.84119	-4.37171	0.34656
1	-0.54482	-2.84962	-1.56629
1	1.02524	-3.46791	-2.04566
1	0.32438	-1.58986	-3.52172
1	1.78819	-1.18961	-2.63996
1	1.7536	1.00259	-2.56095
1	0.30405	1.9251	-2.13323
1	0.37792	1.03614	-3.67814
1	-1.69038	0.90652	-2.18091
1	-1.62453	-0.56267	-3.13255
1	-1.70105	-1.48506	2.12321
1	-4.31883	-0.11813	-2.92476
1	-5.10798	-1.66548	-2.60036
1	-3.53204	-1.62719	-3.37789
1	-6.41429	-0.43844	-0.95378
1	-7.44171	-1.72696	-0.27347
1	-6.47028	-0.69851	0.8117
1	-4.69861	-3.39535	2.0542
1	-3.58577	-2.54554	3.13886
1	-5.09062	-1.78072	2.62456
6	-0.7907	2.1056	1.28567
6	-4.04759	4.22113	-0.62444
6	-3.25273	2.62331	1.00441
6	-4.30503	3.31209	0.39962
1	0.55683	3.80626	1.44751
1	0.9213	2.48073	2.56794
1	-2.52427	5.14911	-1.83343
1	-0.6647	3.939 -0.75353	
1	-5.32411	3.13483	0.72771
1	-4.86463	4.75484	-1.09861
1	-3.46198	1.91338	1.79901
1	2.10555	-0.2705	4.87064
1	0.37121	-0.37082	5.2521
1	2.87103	-3.08717	-0.8987

^{5}[Λ-Cat·R] in H_2O

7	0.21993	0.63109	2.1687
25	-0.1248	-0.39353	0.09842
7	-2.19818	-0.45148	0.18371
7	-0.59803	-1.68785	-1.76752
7	-0.00582	-2.34367	1.04825
7	1.91478	-0.73484	-0.08442
8	-6.31303	-0.53333	0.24737
8	5.90956	-1.62193	-0.50041
6	-2.88187	0.09012	1.21121
6	-4.26295	0.10365	1.27645
6	-4.95122	-0.48908	0.20355
6	-4.26007	-1.08634	-0.8603
6	-2.85941	-1.03199	-0.83462
6	-2.05668	-1.56819	-2.00241
6	-4.98914	0.69796	2.45087
6	-7.00048	0.53488	-0.43941
6	-5.00049	-1.76281	-1.98385
6	0.14302	-1.2808	-2.98312
6	-0.19967	-3.05502	-1.35224
6	-0.60325	-3.33034	0.0839
6	-0.74762	-2.45595	2.33938
6	1.44102	-2.64989	1.31944
6	2.40493	-1.85226	0.48336
6	3.75323	-2.20726	0.37529
6	4.58921	-1.32724	-0.33043
6	4.08004	-0.16517	-0.935
6	2.7262	0.07974	-0.78161
6	4.28955	-3.47918	0.97855
6	6.78721	-1.24244	0.58126
6	4.95992	0.75839	-1.73103
8	-0.03848	1.16205	-0.82337
6	0.35983	4.39426	0.57852
6	1.28383	5.43872	0.64183
6	2.20437	5.61917	-0.38818
6	2.19236	4.75306	-1.48312
6	1.26476	3.7159	-1.54589
6	0.33823	3.52299	-0.51355
6	-0.69543	2.40891	-0.59204
6	-1.7273	2.65806	-1.69596
6	0.64188	1.24768	3.04437
1	-2.2869	0.52271	2.00543
1	-2.20958	-0.88289	-2.84006
1	-2.46539	-2.52933	-2.32907
1	-5.68976	-0.02227	2.87847
1	-4.28916	1.00288	3.22931
1	-5.56996	1.57673	2.15987
1	-6.76994	0.52286	-1.50672
1	-8.06131	0.35014	-0.28887
1	-6.72986	1.50411	-0.01589
1	-6.06205	-1.82869	-1.75725
1	-4.63387	-2.77891	-2.14726
1	-4.88514	-1.2207	-2.92689
1	-0.07274	-0.23686	-3.20074
1	-0.1339	-1.89988	-3.84369
1	1.21283	-1.38435	-2.81068
1	0.8816	-3.13788	-1.46767
1	-0.64734	-3.81305	-2.00564
1	-1.68564	-3.27323	0.19422
1	-0.30909	-4.34347	0.37154
1	-1.8076	-2.28308	2.17371
1	-0.61769	-3.45743	2.7586
1	-0.37079	-1.72075	3.04518
1	1.60716	-3.72339	1.21222
1	1.63707	-2.41504	2.36814
1	2.24622	0.94711	-1.21469
1	4.33174	-3.42258	2.07029
1	5.29294	-3.68268	0.61079
1	3.66409	-4.33687	0.72144
1	6.50971	-1.74964	1.50754
1	7.78406	-1.55176	0.27654
1	6.76542	-0.1614	0.73352
1	5.73177	1.21355	-1.10553
1	5.47265	0.21667	-2.52894
1	4.37542	1.56196	-2.17966
1	-0.35311	4.2599	1.38674
1	1.28448	6.10694	1.49697
1	2.92578	6.42831	-0.34071
1	2.90431	4.8888	-2.29098
1	1.26093	3.05217	-2.4043
1	-1.21724	2.37239	0.37102
1	-2.22136	3.62171	-1.54662
1	-2.4925	1.87903	-1.69307
1	-1.24702	2.66686	-2.6772
6	1.16885	2.02452	4.14748
1	0.46984	2.0007	4.9848
1	1.31538	3.05861	3.83178
1	2.1255	1.60873	4.46775

^{5}[Λ-Cat·S] in H_2O

25	0.41265	-0.25909	0.18901
7	2.44321	0.06966	-0.0722
7	1.06097	-2.31011	-0.1271
7	0.04912	-0.36285	-2.09915
7	-1.57832	-0.85543	0.14293
8	6.4611	0.58645	-0.78432
8	-5.50566	-2.07227	-0.07552
6	2.97274	1.30478	-0.12947
6	4.32018	1.5316	-0.34983
6	5.13602	0.39968	-0.52197
6	4.59787	-0.89629	-0.49515
6	3.22536	-1.0099	-0.25143
6	2.56451	-2.35284	-0.09421
6	4.88044	2.92486	-0.42608
6	7.34751	0.50133	0.35187
6	5.46202	-2.10823	-0.72478
6	0.5493	-3.28567	0.88046
6	0.53381	-2.71509	-1.47461
6	0.70866	-1.62542	-2.51577
6	0.60433	0.78078	-2.85743
6	-1.42277	-0.40172	-2.26327
6	-2.19298	-0.88998	-1.05421
6	-3.53022	-1.28989	-1.17996
6	-4.21657	-1.63339	-0.00618
6	-3.57569	-1.60589	1.24461
6	-2.25104	-1.20798	1.25566
6	-4.21002	-1.35314	-2.52271
6	-6.5281	-1.05821	0.02618
6	-4.28022	-2.019	2.50679
7	0.70733	-0.48623	2.49703
8	0.06485	1.51491	0.30702
6	1.05658	-0.38422	3.58914
6	1.4909	-0.25981	4.96552
6	-2.05093	2.70576	0.4132
1	-1.17572	1.52692	1.96249
1	-0.71475	3.90089	2.5257
6	-0.06312	3.35255	1.84071
6	-1.90849	3.38272	-0.80395
6	-3.02398	3.87143	-1.48035
1	2.267	2.11403	0.00717
1	1.59807	-1.24997	5.41097
1	2.86546	-2.76189	0.87256
1	4.08363	3.66811	-0.38656
1	5.44193	3.068	-1.35167
1	5.56732	3.12643	0.39993
1	7.31138	-0.49285	0.8019
1	8.34514	0.69462	-0.03492
1	7.0887	1.25287	1.10038
1	5.02196	-2.77629	-1.46863
1	5.59515	-2.68781	0.19356
1	6.44498	-1.81497	-1.08671
1	-0.53695	-3.30541	0.85905
1	0.88201	-2.99661	1.87365
1	0.92299	-4.2875	0.6518
1	-0.51968	-2.96139	-1.35019
1	1.03727	-3.62939	-1.80086
1	0.31685	-1.97844	-3.47699
1	1.7677	-1.41276	-2.66713
1	1.68641	0.8052	-2.74346
1	0.19365	1.70578	-2.45912
1	0.36618	0.70433	-3.92421
1	-1.76317	0.61818	-2.46256
1	-1.68843	-0.98812	-3.14743
1	-1.69357	-1.17318	2.18305
1	-4.30749	-0.36028	-2.97075
1	-5.20415	-1.7834	-2.42849
1	-3.6472	-1.97337	-3.22472
1	-6.44923	-0.34504	-0.79711
1	-7.47651	-1.58668	-0.03295
1	-6.45889	-0.52946	0.97865
1	-4.78805	-2.97583	2.372
1	-3.57619	-2.11335	3.33414
1	-5.03993	-1.28911	2.79847
6	-0.83216	2.20604	1.17363
6	-4.30157	3.69553	-0.9456
6	-3.33397	2.53393	0.93931
6	-4.45355	3.02791	0.2681
1	0.3113	4.0514	1.08883
1	0.78492	2.96092	2.40683
1	-2.89703	4.39262	-2.42384
1	-0.91855	3.52845	-1.22281
1	-5.44255	2.88581	0.69171
1	-5.17046	4.07729	-1.47165
1	-3.46176	2.01209	1.8833
1	2.45155	0.25611	5.00407
1	0.75541	0.31104	5.5343
1	2.91952	-3.06224	-0.8443

^{5}[Λ-Cat·R] in CH_3CN*

7	0.21851	0.61515	2.17957
25	-0.13127	-0.39281	0.09904
7	-2.20492	-0.44198	0.18281
7	-0.60946	-1.67015	-1.77712
7	-0.02085	-2.3517	1.03356
7	1.90685	-0.74116	-0.08488
8	-6.31998	-0.50743	0.24518
8	5.89942	-1.63729	-0.50191
6	-2.88685	0.09404	1.21439
6	-4.26789	0.11317	1.27925
6	-4.9584	-0.46765	0.2012
6	-4.26919	-1.05852	-0.8677
6	-2.86828	-1.01058	-0.84098
6	-2.06698	-1.53998	-2.01291
6	-4.99224	0.7011	2.45799
6	-7.00581	0.5643	-0.43783
6	-5.01276	-1.72157	-1.99715
6	0.13543	-1.25726	-2.98837
6	-0.21949	-3.04276	-1.37221
6	-0.62517	-3.32662	0.06177
6	-0.76127	-2.47038	2.32486
6	1.42465	-2.6687	1.29964
6	2.39252	-1.86526	0.47361
6	3.73996	-2.2233	0.36503
6	4.58015	-1.33969	-0.33147
6	4.07553	-0.17083	-0.92678
6	2.72218	0.07698	-0.7735
6	4.27185	-3.50181	0.95818
6	6.77716	-1.27225	0.58461
6	4.95963	0.75696	-1.71311
8	-0.03876	1.16805	-0.81184
6	0.43556	4.37927	0.59905
6	1.37637	5.40928	0.65074
6	2.27314	5.5883	-0.40019
6	2.22054	4.73545	-1.50429
6	1.27631	3.71284	-1.55524
6	0.37326	3.52149	-0.50198
6	-0.67703	2.42236	-0.56697
6	-1.72294	2.68747	-1.65364
6	0.63889	1.22291	3.06221
1	-2.29031	0.51772	2.01225
1	-2.21463	-0.84583	-2.84426
1	-2.48052	-2.49563	-2.34959
1	-5.69329	-0.02088	2.88191
1	-4.29131	1.00073	3.2377
1	-5.57257	1.58207	2.17282
1	-6.77307	0.55785	-1.50469
1	-8.0669	0.3787	-0.29051
1	-6.73618	1.53121	-0.00845
1	-6.07331	-1.7921	-1.76722
1	-4.64555	-2.73479	-2.17589
1	-4.90227	-1.16666	-2.93335
1	-0.07408	-0.21023	-3.19711
1	-0.144	-1.86735	-3.85449
1	1.2044	-1.36837	-2.81556
1	0.8614	-3.13115	-1.48779
1	-0.67136	-3.79319	-2.03154
1	-1.70718	-3.26303	0.17251
1	-0.33823	-4.3441	0.34138
1	-1.82057	-2.29004	2.16251
1	-0.63652	-3.47592	2.7359
1	-0.37899	-1.74292	3.0358
1	1.58549	-3.74152	1.17781
1	1.62242	-2.44949	2.3514
1	2.24583	0.94969	-1.19986
1	4.31732	-3.45286	2.0502
1	5.27341	-3.70722	0.58653
1	3.64214	-4.35512	0.69706
1	6.49453	-1.78519	1.50619
1	7.77261	-1.58543	0.27939
1	6.76199	-0.19233	0.74541
1	5.72922	1.20736	-1.08138
1	5.4754	0.21989	-2.51214
1	4.37787	1.56408	-2.15889
1	-0.25909	4.24648	1.42332
1	1.40829	6.06776	1.51279
1	3.00722	6.38645	-0.36212
1	2.91358	4.8706	-2.3285
1	1.24074	3.05935	-2.42075
1	-1.18379	2.38946	0.40424
1	-2.20129	3.65747	-1.49461
1	-2.49848	1.91887	-1.63981
1	-1.2588	2.69197	-2.64259
6	1.16319	1.98857	4.17449
1	0.43961	1.99902	4.991
1	1.35881	3.01352	3.85557
1	2.0933	1.5395	4.52638

^{5}[Λ-Cat·S] in CH_3CN*

25	0.4043	-0.29025	0.19191
7	2.43296	0.05716	-0.06327
7	1.04638	-2.30598	-0.30958
7	0.02021	-0.18725	-2.09379
7	-1.58758	-0.87945	0.10696
8	6.44467	0.62523	-0.77076
8	-5.52258	-2.05707	-0.1722
6	2.96329	1.29235	-0.02209
6	4.30902	1.53672	-0.2358
6	5.1219	0.42254	-0.50981
6	4.5823	-0.87108	-0.58459
6	3.21199	-1.00399	-0.33946
6	2.55014	-2.35452	-0.28831
6	4.8678	2.93227	-0.20293
6	7.33689	0.4926	0.35621
6	5.44318	-2.06085	-0.91861
6	0.53827	-3.36817	0.60828
6	0.50801	-2.58743	-1.68469
6	0.67315	-1.40931	-2.62576
6	0.5708	1.01535	-2.75902
6	-1.45324	-0.20783	-2.24799
6	-2.21423	-0.80387	-1.08228
6	-3.5539	-1.18801	-1.23014
6	-4.22943	-1.63486	-0.08512
6	-3.57449	-1.72817	1.15513
6	-2.24904	-1.33498	1.18914
6	-4.24679	-1.13186	-2.56664
6	-6.53478	-1.03672	-0.0327
6	-4.26852	-2.25743	2.37889
7	0.714	-0.73135	2.47044
8	0.06962	1.46515	0.48117
6	1.05055	-0.71753	3.57119
6	1.46838	-0.70488	4.95831
6	-2.0122	2.70989	0.56959
1	-1.29684	1.32661	2.0279
1	-0.8149	3.59821	2.90991
6	-0.12685	3.11146	2.21406
6	-1.75113	3.55535	-0.51563
6	-2.79474	4.15154	-1.21951
1	2.25936	2.0874	0.18836
1	1.43545	-1.71705	5.36428
1	2.85383	-2.83943	0.64204
1	4.06877	3.67022	-0.12592
1	5.44671	3.14066	-1.10488
1	5.53758	3.07609	0.6489
1	7.29939	-0.51937	0.76518
1	8.33303	0.69837	-0.02791
1	7.08519	1.21395	1.13618
1	5.00794	-2.65269	-1.72729
1	5.56285	-2.72509	-0.05765
1	6.43169	-1.74031	-1.23981
1	-0.54825	-3.38024	0.59471
1	0.88093	-3.1739	1.62093
1	0.90436	-4.34599	0.28385
1	-0.54441	-2.84433	-1.57279
1	1.00716	-3.46915	-2.09628
1	0.27122	-1.67519	-3.61045
1	1.73047	-1.18324	-2.76837
1	1.6536	1.03173	-2.64959
1	0.16092	1.90468	-2.28608
1	0.32661	1.02454	-3.82713
1	-1.79099	0.82721	-2.34764
1	-1.72831	-0.70852	-3.18072
1	-1.68246	-1.38576	2.1103
1	-4.36883	-0.10195	-2.91404
1	-5.23097	-1.59045	-2.50721
1	-3.68016	-1.66849	-3.33128
1	-6.4523	-0.29516	-0.82988
1	-7.48841	-1.55362	-0.10713
1	-6.45653	-0.54301	0.93788
1	-4.72744	-3.22703	2.17515
1	-3.56735	-2.3732	3.20599
1	-5.06677	-1.58776	2.70862
6	-0.87226	2.08228	1.35715
6	-4.11882	3.91536	-0.84488
6	-3.34056	2.47951	0.93602
6	-4.3891	3.07972	0.23694
1	0.32553	3.8811	1.58418
1	0.6632	2.62439	2.78986
1	-2.57592	4.80346	-2.05917
1	-0.72473	3.7475	-0.80988
1	-5.41492	2.89072	0.53651
1	-4.93196	4.38053	-1.39241
1	-3.56088	1.82714	1.77604
1	2.48723	-0.32179	5.03364
1	0.80217	-0.06459	5.53844
1	2.90185	-3.0022	-1.09397

^{5}[Λ-Cat·R] in CH_3CN**

7	0.24013	0.7682	2.05869
25	-0.1252	-0.39737	0.10146
7	-2.17335	-0.46153	0.20792
7	-0.58292	-1.79525	-1.65532
7	0.01464	-2.24615	1.16765
7	1.89238	-0.71768	-0.08112
8	-6.26875	-0.51729	0.26934
8	5.86274	-1.61469	-0.50841
6	-2.85129	0.15012	1.1921
6	-4.22906	0.17302	1.25451
6	-4.91564	-0.48994	0.22713
6	-4.22799	-1.16125	-0.78792
6	-2.83155	-1.1077	-0.76368
6	-2.03466	-1.72939	-1.88111
6	-4.95717	0.85074	2.36993
6	-6.91766	0.47431	-0.53307
6	-4.96231	-1.91079	-1.85553
6	0.13515	-1.44335	-2.88732
6	-0.15094	-3.11266	-1.16137
6	-0.55771	-3.29776	0.27993
6	-0.72804	-2.27363	2.44799
6	1.45139	-2.50704	1.46511
6	2.39603	-1.78408	0.55589
6	3.73847	-2.14266	0.4483
6	4.55276	-1.31717	-0.33695
6	4.02918	-0.20475	-1.00999
6	2.68063	0.04585	-0.8478
6	4.28814	-3.35773	1.12955
6	6.72874	-1.1668	0.53885
6	4.88867	0.65682	-1.87833
8	-0.07176	1.08106	-0.93265
6	0.21186	4.32274	0.49054
6	1.12532	5.36825	0.59788
6	2.11799	5.52596	-0.3623
6	2.18666	4.63848	-1.43325
6	1.26958	3.60013	-1.53988
6	0.27462	3.42767	-0.57552
6	-0.73628	2.30753	-0.70228
6	-1.72967	2.56246	-1.82776
6	0.72499	1.47486	2.82656
1	-2.25339	0.63576	1.95586
1	-2.21523	-1.12806	-2.77755
1	-2.42977	-2.72547	-2.11213
1	-5.66179	0.16467	2.845
1	-4.26238	1.21464	3.12782
1	-5.5359	1.70421	2.00691
1	-6.67084	0.3465	-1.59015
1	-7.98568	0.32957	-0.38591
1	-6.63416	1.47937	-0.21098
1	-6.02469	-1.96452	-1.62446
1	-4.59285	-2.93537	-1.94752
1	-4.84765	-1.43541	-2.83452
1	-0.1067	-0.41746	-3.16179
1	-0.12973	-2.11781	-3.71008
1	1.20941	-1.51001	-2.71601
1	0.9347	-3.17205	-1.26601
1	-0.57254	-3.92403	-1.76785
1	-1.6438	-3.24515	0.37612
1	-0.25505	-4.28569	0.64116
1	-1.79222	-2.14347	2.26264
1	-0.57502	-3.23517	2.94717
1	-0.37675	-1.47322	3.0958
1	1.63227	-3.58528	1.46822
1	1.64672	-2.16264	2.48531
1	2.1848	0.87985	-1.32997
1	4.34187	-3.22361	2.21421
1	5.28925	-3.58104	0.76423
1	3.6654	-4.23552	0.94017
1	6.45358	-1.6152	1.49684
1	7.73013	-1.48677	0.25923
1	6.70038	-0.07758	0.62412
1	5.6912	1.12673	-1.30418
1	5.36226	0.06538	-2.66559
1	4.30111	1.44795	-2.34586
1	-0.56258	4.20523	1.24469
1	1.06154	6.05714	1.43475
1	2.83416	6.33751	-0.27947
1	2.9562	4.75844	-2.18992
1	1.32579	2.91698	-2.3826
1	-1.29136	2.25905	0.2446
1	-2.23974	3.51861	-1.68336
1	-2.4839	1.77188	-1.85982
1	-1.21419	2.58937	-2.7915
6	1.32979	2.36287	3.78699
1	0.63297	2.55304	4.60483
1	1.58175	3.3071	3.30043
1	2.23846	1.90934	4.1864

^{5}[Λ-Cat·S] in CH_3CN**

25	0.42941	-0.25491	0.22078
7	2.43507	0.08452	-0.0314
7	1.05031	-2.26295	-0.19765
7	0.0977	-0.21179	-2.04936
7	-1.5395	-0.82304	0.12612
8	6.415	0.61556	-0.82355
8	-5.46701	-1.94814	-0.1797
6	2.96298	1.31503	-0.04136
6	4.30247	1.54747	-0.28601
6	5.10455	0.42522	-0.53785
6	4.56437	-0.86587	-0.55648
6	3.20322	-0.98424	-0.2808
6	2.5394	-2.32049	-0.15598
6	4.86847	2.93105	-0.30756
6	7.30098	0.4875	0.29237
6	5.41004	-2.0622	-0.86459
6	0.5233	-3.27741	0.74237
6	0.53412	-2.58177	-1.55825
6	0.74677	-1.44421	-2.52658
6	0.66869	0.95986	-2.72577
6	-1.36021	-0.22882	-2.23439
6	-2.14369	-0.77257	-1.06924
6	-3.48164	-1.14216	-1.22768
6	-4.17668	-1.54756	-0.0846
6	-3.54461	-1.61277	1.16506
6	-2.21807	-1.2362	1.2078
6	-4.14765	-1.111	-2.56836
6	-6.4368	-0.90026	-0.08652
6	-4.26512	-2.09203	2.38353
7	0.71976	-0.60552	2.48661
8	0.09984	1.50657	0.45233
6	1.05235	-0.52935	3.58538
6	1.46266	-0.43868	4.96421
6	-2.08631	2.52985	0.50895
1	-1.2041	1.34703	2.0438
1	-0.94242	3.72036	2.71291
6	-0.22406	3.2439	2.04075
6	-1.93083	3.26139	-0.66997
6	-3.03911	3.69586	-1.38679
1	2.26009	2.11787	0.15315
1	1.46876	-1.43307	5.41342
1	2.83076	-2.74426	0.80944
1	4.07693	3.67799	-0.23648
1	5.43072	3.10564	-1.22742
1	5.55766	3.09362	0.52563
1	7.25446	-0.51906	0.71633
1	8.30057	0.67693	-0.09276
1	7.05995	1.22076	1.06603
1	4.96114	-2.67262	-1.65251
1	5.53495	-2.70366	0.01293
1	6.39697	-1.75642	-1.20745
1	-0.56445	-3.28354	0.71023
1	0.84964	-3.04781	1.75442
1	0.88766	-4.27059	0.46208
1	-0.52881	-2.80808	-1.45905
1	1.01803	-3.49139	-1.92803
1	0.37435	-1.73327	-3.51745
1	1.81386	-1.23737	-2.63979
1	1.75174	0.96324	-2.60552
1	0.26895	1.86486	-2.27157
1	0.43636	0.95431	-3.79722
1	-1.69362	0.80424	-2.37947
1	-1.62256	-0.76299	-3.15436
1	-1.66363	-1.26751	2.13945
1	-4.22916	-0.08998	-2.95335
1	-5.14788	-1.5361	-2.51011
1	-3.58595	-1.69308	-3.3039
1	-6.29995	-0.16786	-0.88632
1	-7.40813	-1.37914	-0.1892
1	-6.37505	-0.39743	0.8818
1	-4.74546	-3.05456	2.1949
1	-3.57984	-2.20427	3.22468
1	-5.05156	-1.39401	2.68291
6	-0.87399	2.08283	1.29723
6	-4.32318	3.4107	-0.92882
6	-3.37473	2.25075	0.95999
6	-4.48805	2.69067	0.2489
1	0.13518	3.99481	1.33191
1	0.62371	2.887	2.63093
1	-2.90189	4.26244	-2.30285
1	-0.93135	3.49374	-1.02516
1	-5.48543	2.46585	0.61441
1	-5.18984	3.75116	-1.4869
1	-3.51211	1.68545	1.87845
1	2.46495	-0.01121	5.02231
1	0.76798	0.19953	5.51261
1	2.89875	-3.02252	-0.91351
